# Tuning the photoactivated anticancer activity of Pt(iv) compounds *via* distant ferrocene conjugation†

**DOI:** 10.1039/d3sc03092j

**Published:** 2024-02-14

**Authors:** Huayun Shi, Fortuna Ponte, Jaspreet S. Grewal, Guy J. Clarkson, Cinzia Imberti, Ian Hands-Portman, Robert Dallmann, Emilia Sicilia, Peter J. Sadler

**Affiliations:** a Department of Chemistry, University of Warwick Coventry CV4 7AL UK p.j.sadler@warwick.ac.uk; b Department of Chemistry and Chemical Technologies, University of Calabria via Pietro Bucci, 87036 Arcavacata di Rende Cs Italy; c Division of Biomedical Sciences, Warwick Medical School CV4 7AL Coventry UK; d School of Life Science, University of Warwick Coventry CV4 7AL UK

## Abstract

Photoactive prodrugs offer potential for spatially-selective antitumour activity with minimal effects on normal tissues. Excited-state chemistry can induce novel effects on biochemical pathways and combat resistance to conventional drugs. Photoactive metal complexes in particular, have a rich and relatively unexplored photochemistry, especially an ability to undergo facile intersystem crossing and populate triplet states. We have conjugated the photoactive octahedral Pt(iv) complex *trans*, *trans*, *trans*-[Pt(N_3_)_2_(OH)_2_(py)_2_] to ferrocene to introduce novel features into a candidate photochemotherapeutic drug. The X-ray crystal structure of the conjugate Pt-Fe confirmed the axial coordination of a ferrocene carboxylate, with Pt(iv) and Fe(ii) 6.07 Å apart. The conjugation of ferrocene red-shifted the absorption spectrum and ferrocene behaves as a light antenna allowing charge transfer from iron to platinum, promoting the photoactivation of Pt-Fe with light of longer wavelength. Cancer cellular accumulation is enhanced, and generation of reactive species is catalysed after photoirradiation, introducing ferroptosis as a contribution towards the cell-death mechanism. TDDFT calculations were performed to shed light on the behaviour of Pt-Fe when it is irradiated. Intersystem spin-crossing allows the formation of triplet states centred on both metal atoms. The dissociative nature of triplet states confirms that they can be involved in ligand detachment due to irradiation. The Pt(ii) photoproducts mainly retain the *trans*-{Pt(py)_2_}^2+^fragment. Visible light irradiation gives rise to micromolar activity for Pt-Fe towards ovarian, lung, prostate and bladder cancer cells under both normoxia and hypoxia, and some photoproducts appear to retain Pt(iv)–Fe(ii) conjugation.

## Abstract 2

DFT calculations on a conjugate of ferrocene to an octahedral Pt(iv) complex reveal Fe-to-Pt charge transfer, promoting longer-wavelength photoactivation, enhanced cellular accumulation, potent anticancer activity, and cell death *via* ferroptosis.

## Graphical abstract

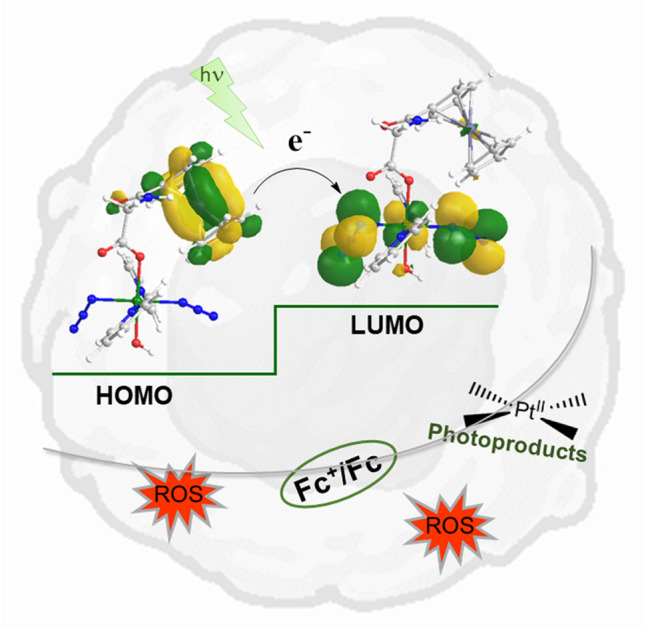

## 1. Introduction

Phototherapy is attractive for the treatment of cancer using compounds with low dark toxicity which are activated selectively in tumours using spatially-directed light.^1–3^ Current clinical applications, as photodynamic therapy (PDT), use so-called Type-II photosensitisers, which rely on conversion of ground-state ^3^O_2_ to highly reactive excited state ^1^O_2_ to kill cancer cells.^2,4^ There is current interest in the design of the next generation of metal-based phototherapeutic agents on account of their unique photophysical and photochemical properties, especially potential for introducing novel mechanisms of anticancer activity to combat resistance to therapy with current drugs.^5–8^ Metal complexes of Al(iii), Zn(ii), Ru(iii), Pd(ii), Sn(iv), and Lu(iii) are either approved for clinical use or in clinical trials as photosensitisers for PDT.^2,5^ Particularly notable is the current phase II clinical trial of the bipyridyl/phenanthroline Ru(iii) complex TLD1433 for non-muscle-invasive bladder cancer.^9^

The most widely used anticancer drug is cisplatin, *cis*-[PtCl_2_(NH_3_)_2_].^10,11^ Notably, its *trans* isomer, transplatin, is inactive. The development of resistance to treatment and the occurrence of side effects have stimulated the search for alternative platinum drugs which overcome these problems.^12,13^ Photoactivated Pt(iv) complexes offer this possibility on account of their kinetic inertness, stability in the dark, and introduction of novel, excited state, mechanisms of action.^14,15^ First-generation photoactive di-iodido Pt(iv) complexes suffered from high dark cytotoxicity owing to their facile bio-reduction.^16,17^ Replacing iodido with azido ligands has led to photoactive diazido Pt(iv) complexes with enhanced dark stability and promising photocytotoxicity.^15^ Such complexes can generate anticancer-active Pt(ii) species, and azidyl and other radicals upon irradiation. Importantly, their action does not rely on the presence of oxygen in the target tissues, suggesting their potential to retain photocytotoxicity under hypoxia.^18^ The activation wavelengths for diazido Pt(iv) complexes are tuneable by modification of either equatorial or axial ligands.^15^ For example, *trans*, *trans*, *trans*-[Pt(N_3_)_2_(OH)_2_(NH_3_)(py)] exhibits promising photocytotoxicity upon irradiation with UVA (365 nm),^19^ while the two-pyridine complex *trans*, *trans*, *trans*-[Pt(N_3_)_2_(OH)_2_(py)_2_] (1) can kill cancer cells when irradiated with blue light (420 nm).^20^ Other than diazido Pt(iv) complexes, the Pt(iv) derivatives of cisplatin^21^ and oxaliplatin^22,23^ also exhibit promising photocytotoxicity with blue, red or NIR irradiation when combined with appropriate light antennas. Hydrogen bond formation or charge transfer between the Pt(iv) centre and the light antennas can facilitate the photoactivation of these Pt(iv) complexes.

Here we report the conjugation of ferrocene ([Fe(η^5^-C_5_H_5_)_2_], Fc) to the octahedral Pt(iv) complex 1 for tuning its photo-reactivity. The usefulness of ferrocene fragments for improving drug design was initially established by its conjugation to the anticancer drug tamoxifen (as ferrocifen),^24^ and to the antimalarial drug chloroquine (as ferroquine).^25^ Since then, many other examples of the success of this approach have been reported.^26–30^

The characteristic electrochemical and photochemical properties, together with UV-vis stability of ferrocene and its derivatives, are well suited to their use in photochemical studies. In particular, ferrocenyl compounds, when linked to platinum and other transition metal complexes, can act as one-electron donor species, tuning the redox chemistry of metal compounds.^31–36^ Several heterobimetallic Pt(ii)-ferrocene conjugates have been reported.^37^ For ferrocene-appended [Pt(L_2_)diamine] complexes, charge-transfer from the ferrocene to the diamine ligand was observed.^31^ Pt(ii) complexes bearing bis-aminoethyl ferrocene ligands can be more active towards cancer cells than cisplatin with a different mechanism of action.^33^ Bridging ferrocene in di-Pt(ii)-ferrocene dyads gives rise to longer-lived charge-separated states upon excitation, but quenches Pt(ii) luminescence.^38^ Introduction of a ferrocene as a substituent on a chelated acetylacetonate Pt(ii)(diam(m)ine) fragment, gave a reported multimodal mechanism of anticancer activity.^39^ Biotinylated Pt(ii) ferocenylterpyridine complexes show significant photoinduced anticancer activity on irradiation with visible light.^40^ Chakravarty *et al.* also reported that ferrocenyl-terpyridine Pt(ii) complexes are highly photo-cytotoxic when activated by visible light and generate hydroxyl radicals.^41^ Two one-electron transfers from Fc to Pt(iv) to generate anticancer-active Pt(II) complexes were observed for Pt(iv)-Fc conjugates, which can elevate ROS level in cancer cells and exhibit promising cancer selectivity.^42,43^ However, the use of Fc conjugates in which the Fc moiety is linked to diazido Pt(iv) complexes for tuning their photoactivity has yet to be reported.

Such conjugates can possess interesting electronic, optical and magnetic features and introduce synergistic biological effects between the two active metals. After photoexcitation, intramolecular electron transfer (ET) processes between the electron donor (D) and the electron acceptor (A) units, can play important roles in formation of the key final charge separation (CS) products. Generally, an effective photoinduced ET in the donor–acceptor dyads and consequently an efficient formation of the CS states with a long lifetime, depends on the nature of the D and A groups, on the spatial arrangement related to the distance between the donor and acceptor units, intermolecular interactions, and charge-recombination (back electron transfer).^44^ Although these processes can be in direct competition with many other radiative and non-radiative deactivation phenomena,^45^ it has been shown that photoexcited platinum complexes can act as good electron acceptors in the presence of the electron donor ferrocene and that Pt(ii) fluorescence can be quenched due to efficient electron-transfer processes.^38,46^

**Scheme 1:**
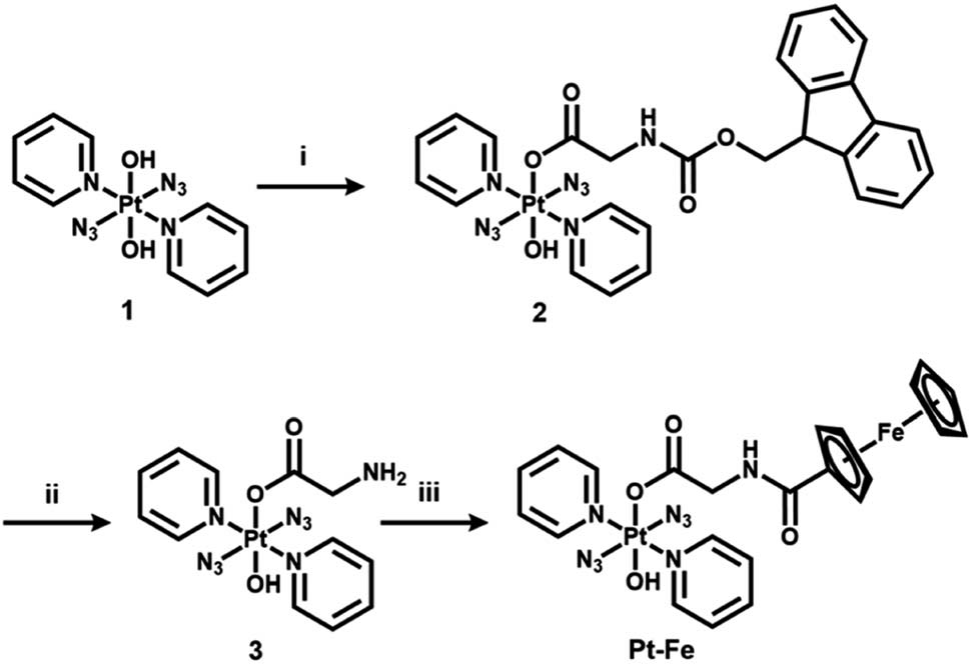
Synthetic route for photoactive heteronuclear complex *trans*, *trans*, *trans*-[Pt(N_3_)_2_(OH)(gly-Fc)(py)_2_] (Pt-Fe). (i) Fmoc-gly-OH, TBTU, DIPEA, DMF, N_2_, 298 K, overnight; (ii) piperidine, DMF, N_2_, 298 K, overnight; (iii) ferrocenoyl chloride, DIPEA, DCM, N_2_, 298 K, overnight.

Here, we report a novel Pt-containing photoactive heterometallic complex *trans*, *trans*, *trans*-[Pt(N_3_)_2_(OH)(gly-Fc)(py)_2_] (Pt-Fe, Scheme 1) in which ferrocene is connected to a diazido Pt(iv) complex through a flexible bridging ligand. This complex was structurally characterised by X-ray analysis, and cyclic voltammetry was used to study its redox behaviour. The dark stability and photoactivation with visible light, photoreaction with guanosine 5′-monophosphate (5′-GMP), photocytotoxicity under various oxygen concentrations and cellular accumulation, generation of ROS and mechanisms of action were also investigated. Density functional theory (DFT) and, its time-dependent extension (TDDFT), were used to investigate photophysical properties of Pt-Fe by the computation of the ground state structure, excited-state energies and orbital character of the spectral transitions, as well as optimisation of the excited-state structure and identification of the dissociative states prior to ligand dissociation. We envisaged that the ferrocene fragment might act both as a photo-initiator to allow photoactivation of the Pt(iv) centre at longer wavelength, and as a catalyst to promote ROS generation.

## 2. Results

### 2.1. Synthesis and characterisation of *trans*, *trans*, *trans*-[Pt(N_3_)_2_(OH)(gly-Fc)(py)_2_] (Pt–Fe)

**Fig. 1:**
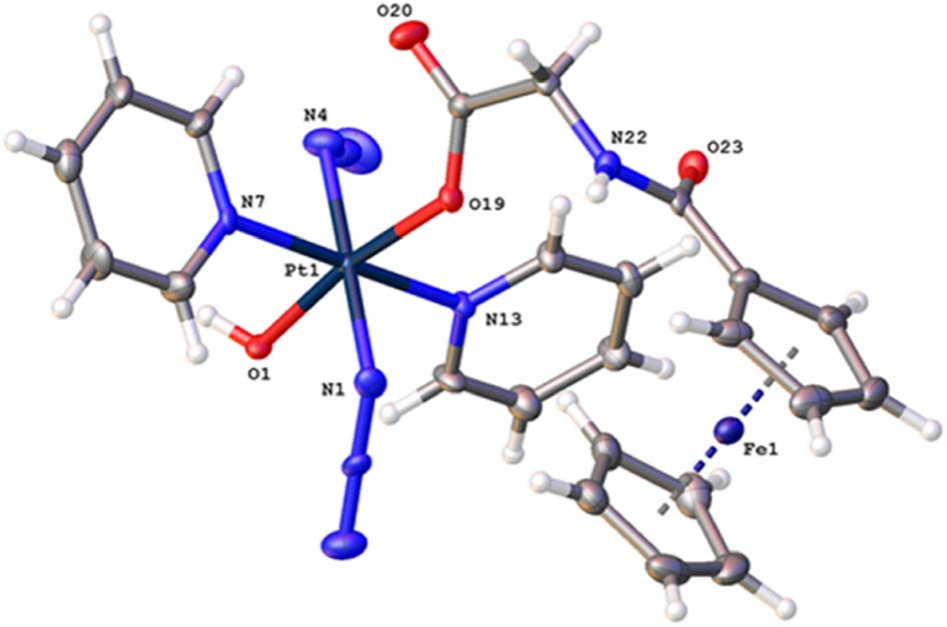
X-ray crystal structure of Pt-Fe with key atoms labeled and thermal ellipsoids drawn at 50% probability level. Crystal packing and H-bonds are shown in Fig. S6 (ESI).†

The synthetic route for Pt(IV) complex Pt-Fe is summarised in Scheme 1. *Trans*, *trans*, *trans*-[Pt(N_3_)_2_(OH)(gly)(py)_2_] (3) with a free terminal NH_2_ group was prepared from parent complex *trans*, *trans*, *trans*-[Pt(N_3_)_2_(OH)_2_(py)_2_] (1) following modified literature procedures.^20,47^ This amine reacts with ferrocenoyl chloride in the presence of DIPEA to give the heteronuclear complex Pt-Fe. Pt-Fe was characterised by elemental analysis, HPLC (Fig. S1, ESI†), ESI-HRMS (Fig. S2, ESI†), NMR (Fig. S3 and S4, ESI†), UV-vis spectroscopy (Table S1 and Fig. S5, ESI†) and X-ray crystallography (Fig. 1).

The ^1^H and ^13^C NMR spectra are consistent with the proposed structure of Pt-Fe. The doublet with relatively sharp ^195^Pt satellites assignable to the ortho protons of Pt(iv)-coordinated pyridine is shifted to high field from 9.09 in 1 to 8.86 ppm for Pt-Fe. ^13^C NMR resonances of pyridine in Pt-Fe have shifts of 149.85, 142.46 and 126.72 ppm, similar to those for 1. The ^1^H NMR singlets at 4.79, 4.32 and 4.13 ppm and ^13^C NMR resonances at 76.80, 70.27, 69.79 and 68.57 ppm are assignable to the ferrocene fragment.

Uv-vis absorption spectra of Pt-Fe in H_2_O, PBS and DMSO were recorded for comparison with 1, Fc and its derivative ferrocene carboxylic acid (Fc-COOH) (Table S1 and Fig. S5, ESI†). Bands at 260 nm (*ε* = 22 364 M^−1^ cm^−1^), 299 nm (*ε* = 21 714 M^−1^ cm^−1^) and 435 nm (broad, *ε* = 528 M^−1^ cm^−1^) with a tail extends to 530 nm were observed for Pt-Fe in water. In comparison with 1, the band of Pt-Fe at 260 nm shows a *ca.* 2× enhancement in intensity, while that at *ca.* 300 nm is redshifted from 293 to 299 nm after conjugation with ferrocene. Notably, the weak broad band for Fc and Fc-COOH at *ca.* 440 is blue-shifted to 435 nm when appended to the Pt complex, and its intensity is enhanced by *ca.* 2×. The extinction coefficients of bands for Pt-Fe in PBS and DMSO are *ca.* 20–40% higher than H_2_O, while those of 1 and Fc-COOH are similar.

### 2.2. X-ray crystallography

Crystals of Pt-Fe suitable for X-ray diffraction were obtained through the diffusion of diethyl ether into a solution of this complex in DCM/methanol. A perspective drawing of complex Pt-Fe is shown in Fig. 1. The crystallographic data are summarised in Table S2 (ESI)† and selected bond distances and angles are listed in Table S3 (ESI).†

Complex Pt-Fe crystallised in the monoclinic space group *P*2_1_/*c* with four molecules within the unit cell. Pt-Fe is a neutral hetero-dinuclear complex consisting of Pt(iv) and Fe(ii) fragments. The platinum moiety is similar to its parent complex 1,^20^ with octahedral [N_4_O_2_] geometry for Pt(IV) slightly distorted due to the presence of the axial gly-Fc ligand. The cyclopentadienyl (Cp) rings are η^5^-bonded to Fe(ii) with average Fe–C distances of 2.04 Å (range 2.036(3)–2.059(3) Å). The distance between two metal centres (Pt⋯Fe) is 6.070 Å (Fig. S6a, ESI†). An ortho pyridine proton is relatively close to the ferrocene, which might be responsible for the induced ^1^H NMR shift (Fig. S6b, ESI†). Hydrogen bonds in Pt-Fe are observed between an axial Pt–O–H and the carbonyl group on the Cp ring (O1–H1⋯O23), and between the amide N–H and an oxygen of the Pt-coordinated carboxylate group (N22–H22⋯O20), as listed in Fig. S6c and Table S4 (ESI).†

### 2.3. Cyclic voltammetry

A cyclic voltammogram for Pt–Fe was acquired over the range −1.8–1.0 V in DMF at 298 K, using 0.1 M NBu_4_PF_6_ as supporting electrolyte (Fig. S7, ESI† and Table 1). This complex exhibited a reversible oxidation wave assigned to Fc^+^/Fc with *E*_pa_ = 0.247 V and *E*_1/2_ = 0.210 V, and an irreversible reduction wave assigned to Pt^IV^/Pt^II^ with *E*_pc_ = −1.466 V. For ferrocene and parent complex 1 alone, the reversible oxidation of the former Fc^+^/Fc was determined as *E*_pa_ = 0.083 V and *E*_1/2_ = 0.042 V, and the irreversible reduction wave of 1 assigned to Pt^IV^/Pt^II^ was determined as *E*_pc_ = −1.699 V.^48^ Significant shifts to higher potential of 168 and 233 mV for Fc^+^/Fc and Pt^IV^/Pt^II^, respectively, were observed for Pt-Fe, compared with free ferrocene and parent Pt(IV) complex 1.

**Table:** Cyclic voltammogram data for Pt-Fe, 1 and Fc

Compound	Fc^+^/Fc			Pt^IV^/Pt^II^
	*E*_pa_	*E*_pc_	*E*_1/2_ [Δ*E*]^a^	*E*_pc_
Pt-Fe	0.247 V	0.172 V	0.210 V [75 mV]	−1.466 V
1	—	—	—	−1.699 V
Fc	0.083	−0.01	0.042 V [84 mV]	—

a *E*_1/2_ = 0.5(*E*_pa_ + *E*_pc_); Δ*E*_p_ = (*E*_pa_ − *E*_pc_), where *E*_pa_ and *E*_pc_ are the anodic and the cathodic peak potentials, respectively. Scan rate = 100 mV s^−1^.

### 2.4. Dark stability and photodecomposition

**Fig. 2:**
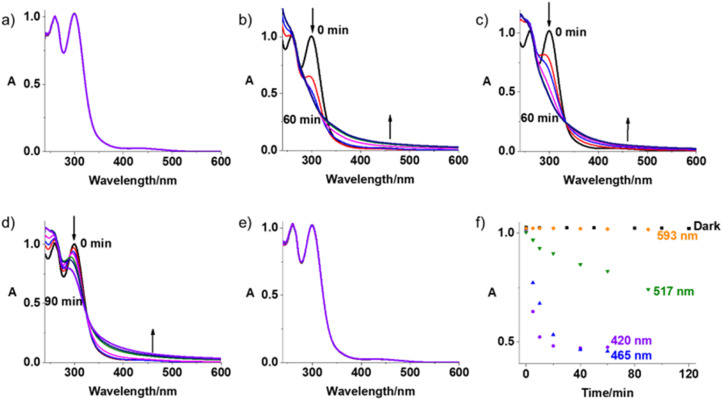
UV-vis spectral changes for Pt-Fe (50 μM, phenol red-free RPMI-1640 with 5% DMSO) in the dark (a, 120 min) or with indigo (b, 420 nm, 60 min), blue (c, 463 nm, 60 min), green (d, 517 nm, 90 min) and orange (e, 593 nm, 90 min) light irradiation at 298 K; (f) time dependent absorbance changes of Pt-Fe at 300 nm upon irradiation with light of different wavelengths.

Complex Pt-Fe exhibits good dark stability in phenol red-free RPMI-1640/5% DMSO (v/v) as monitored by UV-vis spectroscopy (Fig. 2a). Good dark stability of Pt-Fe in aqueous solution at 310 K for 24 h was determined by LC-MS (Fig. S8, ESI†). Dark stability of Pt-Fe towards the bio-reductant ascorbic acid, in air and N_2_ saturated PBS was observed at 310 K over 2 h (Fig. S9, ESI†). Reduction of only 12% of Pt-Fe was observed with release of the axial ligands after 2 h incubation, as monitored by LC-MS (Fig. S10, ESI†). However, when the incubation time increased to 24 h, 43% of Pt-Fe underwent reduction (Fig. S10, ESI†). In addition, when incubated with a high concentration of GSH in the dark for 24 h, all Pt-Fe underwent reduction (Fig. S11, ESI†).

The photodecomposition of complex Pt-Fe (50 μM) in both air- and N_2_-saturated cell culture medium RPMI-1640 was also monitored by UV-vis spectroscopy at different time intervals after irradiation with indigo (420 nm), blue (463 nm), green (517 nm), and orange (593 nm) light at 298 K (Fig. 2 and S12, ESI†). Irradiation with indigo, blue and green light decreased the intensity at the absorbance maximum of Pt-Fe in air saturated RPMI-1640 at 300 nm. An apparent increase in intensity of the high-energy band at 260 nm and the absorbance at > 350 nm was observed. Notably, the decrease in absorbance of Pt-Fe at 300 nm with green light irradiation, though not as large as that with blue light, is > 2× as high as that detected for complex 1 under the same irradiation conditions (Fig. S13, ESI†). However, no apparent decrease at 300 nm was observed after 120 min irradiation with orange light. For the N_2_-saturated solution, similar spectral changes were observed as those in air-saturated solution for the same irradiation conditions with blue (463 nm), green (517 nm) and orange (593 nm) light (Fig. S12, ESI†).

To investigate the species formed upon irradiation with indigo (420 nm), green (517 nm) and orange (593 nm) light, LC-MS was employed to monitor the photoactivation of Pt-Fe (30 μM) in aqueous solution. The HPLC peak assigned to complex Pt-Fe (retention time 16.5 min) decreased gradually with indigo light exposure time, and disappeared within 15 min, more than 2× as fast as for parent complex 1 (Fig. S14 and S15, ESI†). To determine the nature of these photodecomposition products, photoproducts were collected from the HPLC eluent and analysed by HR-MS. The major photodecomposition products detected after 1 h irradiation included {Pt^II^(CH_3_CN)_2_(py)_2_]^2+^ (217.5507 *m*/*z*), [{Pt^II^(py)_2_(N_3_)_2_}+K]^+^ (476.0246 *m*/*z*), {Pt^II^(py)_2_(CH_3_CN)(HCOO)}^+^ (439.0676 *m*/*z*), {Pt^IV^(gly-Cp-Fe)(py)_2_(N_3_)_2_(OH)}^+^ (675.1004 *m*/*z*), and {Pt^II^(py)_2_(N_3_)(CH_3_CN)}^+^ (436.0846 *m*/*z*). Intermediates [{Pt^II^(gly-Fc)(py)_2_(OH)}+H]^+^ (657.1353 *m*/*z*), {Pt^IV^(gly-Fc)(py)_2_(N_3_)(OH)}^+^ (698.0674 *m*/*z*), and {Pt^IV^(gly-Fc)(py)_2_(N_3_)_2_}^+^ (723.1282 *m*/*z*) formed, but then decomposed after 1 h irradiation (Fig. S14 and Table S5, ESI†). CH_3_CN and HCOOH are from the mobile phase. For comparison, the number of photoproducts from 1 is much less than Pt-Fe due to the absence of the ferrocene-containing axial ligand (Fig. S15 and Table S6, ESI†). {Pt^III^(py)_2_(OH)_2_}^+^ (386.0540 *m*/*z*), {Pt^III^(py)_2_(N_3_)(HCOO)}^+^ (440.0687 *m*/*z*), and {Pt^II^(py)_2_(N_3_)(CH_3_CN)}^+^ (436.0846 *m*/*z*) are assigned as the main photoproducts of 1 after 1 h irradiation at 420 nm, indicating the release of one or two azido ligands. The Pt(III) species might be formed in the ionisation process. More than 80% of Pt-Fe decomposed after 1 h irradiation with green light (517 nm), yielding three main peaks (Fig. S16 and Table S7, ESI†). These peaks can be assigned to the Pt(II) species {Pt^IV^(gly-Cp-Fe)(py)_2_(N_3_)_2_(OH)}^+^ (675.1004 *m*/*z*), {Pt^II^(py)_2_(N_3_)(CH_3_CN)}^+^ (436.0846 *m*/*z*) and {Pt^II^(gly-Fc)(N_3_)(py)(CH_3_CN)}^+^ (643.1118 *m*/*z*) resulting from the release of one or two azido ligands or axial ligands. In contrast, complex 1 showed negligible decomposition after the same irradiation, and no apparent photodecomposition products could be detected by LC-MS.^47^ Upon irradiation with orange light (593 nm), only about 2% of complex Pt-Fe decomposed after 2 h irradiation (Fig. S17, ESI†). However, same Pt photoproducts were detectable despite their low intensity (Table S7, ESI†).

### 2.5. DFT and TDDFT calculations

The optimised molecular structure of Pt-Fe obtained at the B3LYP-D3/SDD/6-31G* level of theory using its X-ray crystal structure as a starting point is shown in Fig. S18 (ESI).† The most relevant geometrical parameters, compared with the experimental counterparts, are listed. The good agreement confirms the suitability of the computational protocol used. The slight differences in the ferrocene Cp rings and in the Pt region are attributable to the difference in crystal *vs.* solvent, environments.

**Fig. 3:**
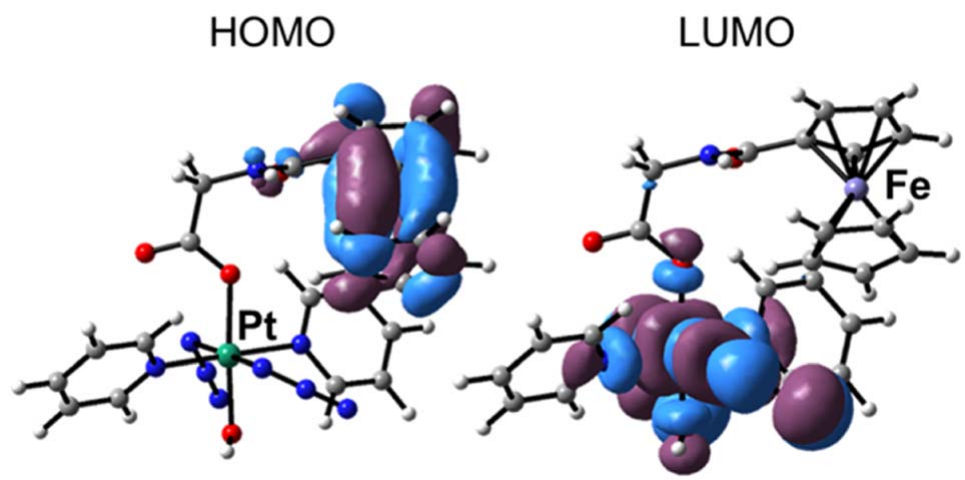
The HOMO orbital of Pt-Fe is centred on ferrocene, while the LUMO is located on the Pt unit.

The picture of frontier molecular orbitals shown in Fig. 3, allows the exploration of the possible communication between the Pt and Fe fragments. The HOMO (H) orbital is centred on ferrocene, while LUMO (L) is located on the Pt unit. Such a characteristic distribution of orbitals is a theoretical indication that, as it will be shown in the next paragraphs, an electronic communication occurs between the two units upon excitation.

#### 2.5.1. Absorption spectra

**Fig. 4:**
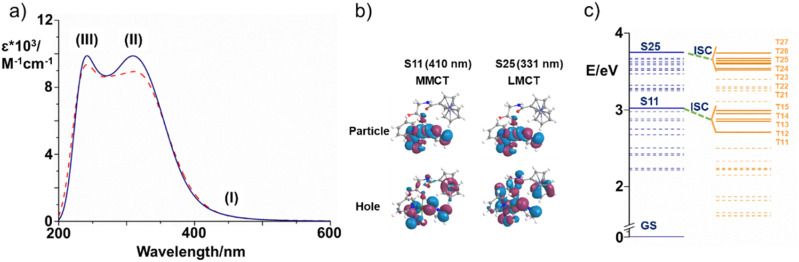
(a) B3LYP UV-vis absorption spectrum of Pt-Fe simulated in both DMSO (blue solid) and water (red dashed). The regions of interest are labelled as (I), (II) and (III); (b) NTO plots for S11 and S25 singlet states and relative theoretical assignments for hole-to-particle transitions. MMCT: Fe(ii) to Pt(IV), and LMCT: ligand-to-Pt (IV), contributions are shown on Fig. 4c, and listed in Table S9†); (c) energy diagram of the low-lying excited singlet (blue) and triplet (orange) states of Pt-Fe computed with respect to the ground-state zero energy. Solid lines identify the most important singlets and triplet states. Only the most probable couplings (ISC) of each singlet state with the triplet states lying below are highlighted (green dashed lines).

The calculated UV-vis spectra of Pt-Fe in both DMSO and in water, that better mimics the physiological environment, are shown in Fig. 4a. The B3LYP-D3 functional was chosen to carry out calculations as a result of a benchmark comparing several functionals (Table S8, ESI†). In Fig. 4a, the most important regions of the calculated spectra are labelled as (I), (II), (III). Both calculated spectra are essentially characterised by a long low-energy tail in the 400–500 nm range (region I) and two further absorption bands falling in the region of 200–400 nm (II and III), consistent with the photophysical features detected experimentally. Due to interest in possible use of Pt-Fe as a photoactive drug, attention was focused on the absorption spectrum simulated in water. The tail of the lowest energy band (I), with a weak absorption in the visible region, originates from different electronic transitions which are described in detail in Table S9 (ESI).† A helpful way of providing a qualitative description of electronic excitations is the use of Natural Transition Orbitals (NTOs). NTOs allow ordinary orbital representations to be transformed into a more compact form in which each excitation is expressed as only a pair of orbitals: the NTO transition occurs from excited hole (occupied orbital) to the empty particle (unoccupied orbital).^49^ Overall, the transition can be described as a metal-to-metal charge-transfer (MMCT), where the CT occurs from the ferrocene Fe atom to the Pt centre, as evidenced by the NTO plots collected in Fig. S19 (ESI).† TDDFT assigns the most intense transition (Tr7), with the largest oscillator strength, to the singlet state at 410 nm originated by the H-5 (Pt 3%, Fe 3%) → L (Pt 40%, Fe 0%), H-2 (Pt 0%, Fe 82%) → L and H-6 (Pt 2%, Fe 3%) → L transitions.

The Pt-Fe spectrum displays an intense absorption band (II) centred at 331 nm (Tr = 8) ascribed to LMCT excitation and originating in H-8 (Pt 6%, Fe 2%) → L (Pt 40%, Fe 0%) MOs. Overall, such a band is described by transitions with different features (Table S9, ESI†) and the low-energy NTOs (Fig. S19, ESI†) for 8–16 transitions show that it is a mixed band with ligand-to-metal (LMCT), ligand-to-ligand (LLCT), and metal-to-metal (MMCT) charge-transfer characters, as expected experimentally. Finally, the absorption band observed in the high energy 200–250 nm range (III) can be assigned to LLCT transitions.

#### 2.5.2. Excited states properties

A detailed TDDFT analysis of the excited states was carried out based on both spin–orbit coupling constants (SOCs) and singlet–triplet (S–T) adiabatic energy differences (Δ*E*) to investigate the behaviour of Pt-Fe under irradiation. When Pt-Fe is irradiated, several singlet states can be, in principle, populated. In agreement with the experimental data, attention was focused on two excitation wavelengths, associated with the S11 and S25 singlet states (Fig. 4b). The excitation wavelength for S11, that falls in the low energy region of the spectrum, was calculated to be 410 nm and corresponds to the transition Tr7. The MMCT nature of the transition involves exclusively charge transfer from the ferrocene Fe atom to the Pt centre. In addition, the spectrum of the complex shows a maximum absorption at 331 nm, which corresponds to the experimentally observed value of 300 nm, associated to the S25 singlet state (Tr8). Charge transfer between the axial gly-Fc ligand and platinum, contributes to the generation of a state with LMCT character. Only the singlet deactivation channels considered more plausible are highlighted in Fig. 4c. In this work, all the energy transfer occurring among states separated by a gap greater than 0.30 eV were discarded. Thus, the couplings between the excited singlet state S25 and the triplets named as T*n* (with *n* = 20–27), which lie below it, were explored together with the couplings between S11 and the underlying triplet states T*m* (with *m* = 11–15) and reported in Table 2. The triplet state character and the MO contributions are described in Table S10 (ESI).† NTO plots are collected in Fig. S20 (ESI).†

Spin orbit coupling constants, SOC, and adiabatic energy gap (in brackets), Δ*E*(S-T), between the S25 and T*n* (with *n* = 20–27) triplet states and S11 and T*m* (with *m* = 11–15) triplet states

**Table:** 

	S11-T15	S11-T14	S11-T13	S11-T12	S11-T11
SOC (cm^−1^)	42	14	310	96	3
Δ*E* (eV)	0.03	0.07	0.14	0.17	0.30

**Table:** 

	S25-T27	S25-T26	S25-T25	S25-T24	S25-T23	S25-T22	S25-T21
SOC (cm^−1^)	10	10	334	65	50	2	6
Δ*E* (eV)	0.01	0.08	0.11	0.14	0.15	0.21	0.23

For all the coupling of the states separated by a small energy difference, Δ*E*, starting from the state S11 and bearing in mind that even internal conversion (IC) processes between states with the same multiplicity can occur, the ISC results are favourable. The small SOC value calculated for S11→T11 (3 cm^−1^) suggests a lower probability that an intersystem crossing can occur, when compared with those calculated for the T13 triplet state, two orders of magnitude larger. On the basis of the analysis of MOs and NTO plots, it is possible to assign to the triplet states T11 and T14 the same ^3^ILCT/^3^MC character. Nevertheless, T14 state (SOC = 14 cm^−1^) has a higher probability to be populated than T11 because of the smaller energy gap between the coupled states, in spite of the similar character. The ^3^MMCT nature of T12, T13 and T15 involves for all of them a charge transfer from the Fe atom to the platinum centre. The states T12 and T13, however, mainly originate from the excitations H (Pt 0%) → L+1 (Pt 47%) and H-1 (Pt 0%) → L+1 (Pt 47%), respectively, involving a higher participation of the metal than the T15 state H-2 (Pt 0%) → L (Pt 40%). It can be assumed that the two couplings, S11-T12 and S11-T13 are driven by large computed SOC values of 96 and 310 cm^−1^, respectively, as a consequence of the involvement of the heavy platinum atom.

In contrast, the low SOC value of the T15 state can be accounted for according to El-Sayed rules.^50^ A significant SOC value is, indeed, calculated when a change of the orbital type accompanies the coupling, whereas the ^3^MMCT character of the T15 state is very similar to that of the S11 state.

There are several possible ISC channels starting from the S25 excited singlet state. It is possible to collect the triplet states below S25 (with ^1^LMCT character) into three groups. In particular, inspection shows for T21 and T22 triplet states, a pure ^3^MLCT character, and the charge transfer in both states involves the Fe centre and the py ligand. As a result, there is no participation of the Pt centre in both triplet states, and the computed SOC values for S25→T21 and S25→T22 couplings, remain in the order of a few cm^−1^, 6 and 2 cm^−1^, respectively.

Charge transfer, with ^3^LMCT character, between several ligands and the Pt atom contributes to the generation of the T23, T24, T26 and T27 triplet states. In all the four states, as shown in the MOs contribution analysis, the percentage of iron participation, instead, is less than 3%. Although the nature of the MOs involved in the transition is very similar to that of the singlet state, the heavy atom effect plays a role in promoting the ISC. In fact, the SOC values are 50, 65, 10 and 10 cm^−1^ for the coupling with T23, T24, T26 and T27, respectively.

The largest coupling value computed for the S25 state involves the T25 triplet state. This triplet state originates mainly from H-2 (Fe 82%, Pt 0%) → L+1 (Pt 40%, Fe 0%) excitation and, inspecting the NTO plot, a considerable participation of the Fe atom in the hole and of the Pt atom in the particle can be observed, determining the ^3^MMCT character of such state. This means that the transition from S25 state to this triplet state, can involve a change of orbital type, thus resulting in a large rate of ISC, as postulated by El-Sayed rules, and the charge transfer that involves the two metal centres, seems to cause a significant enhancement of the SOC values.

#### 2.5.3. Triplet state features

The geometries of the states T*n* and T*m*, found within the vertical approximation and considered in the SOC calculations, were primarily optimised at the TD-B3LYP level of theory within the unrestricted Kohn–Sham formalism (UKS).^51,52^ The optimised structures obtained by the TDDFT calculations and the spin density distributions are shown in Fig. S21 and S22 (ESI).† All the examined triplet excited states collapsed into three different optimised triplets, named *T*_a_, *T*_b_ and *T*_c_, for which the optimised structures are shown in Table 3 together with the relative spin density distributions used to establish their character, and the relative energies calculated with respect the ground state S0. The main bond distances for the calculated triplet states are compared with those of the S0 ground state in Table 3.

**Table:** Optimised structures and spin density distributions of the calculated triplet states and their most relevant bond lengths (in Å) compared with GS corresponding values, spin densities values (SD in a.u.) for Pt and Fe atoms and adiabatic energy gap (Δ*E* in eV) calculated respect to the GS. Orange colour identifies the atoms where unpaired electrons are localised

		*S*_0_	*T*_a_	*T*_b_	*T*_c_
Optimised structures			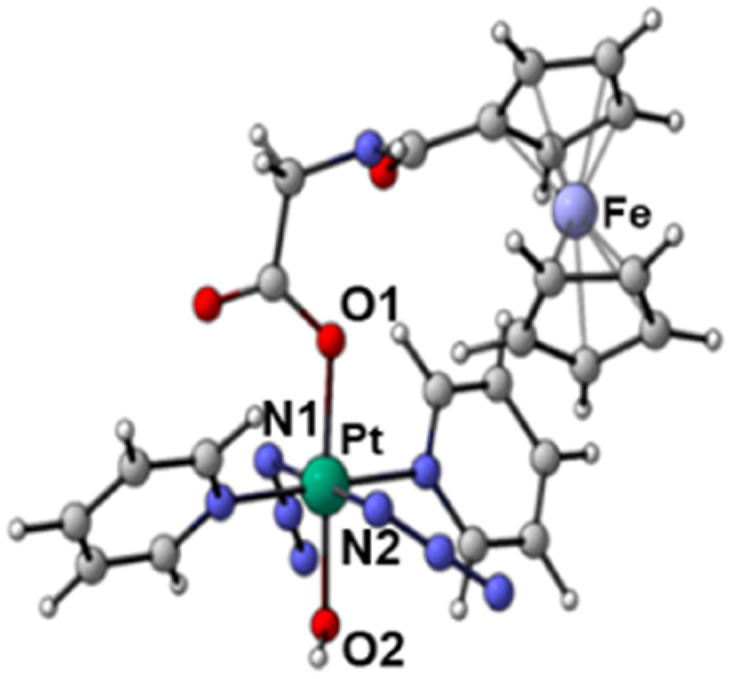	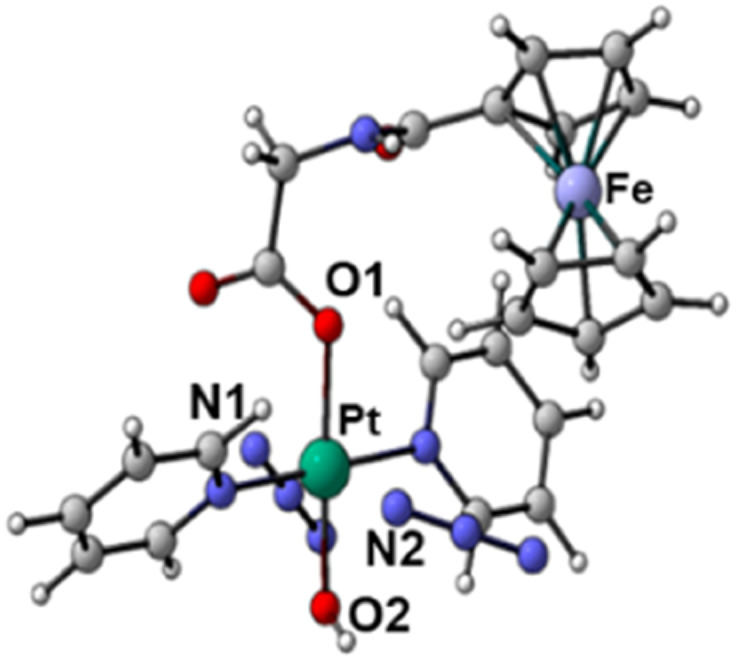	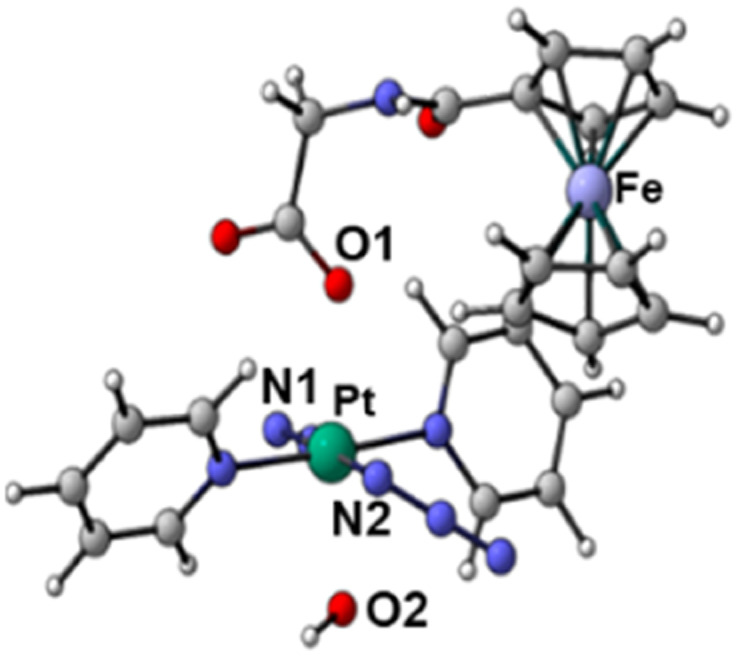
Spin density distributions			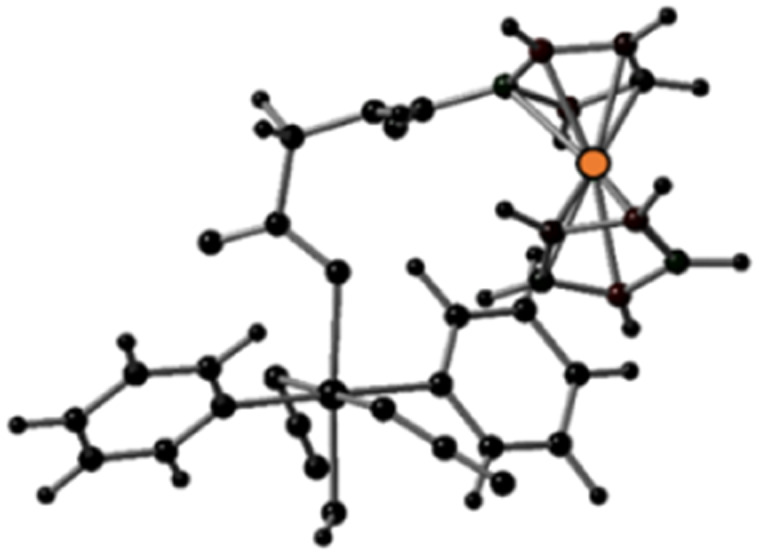	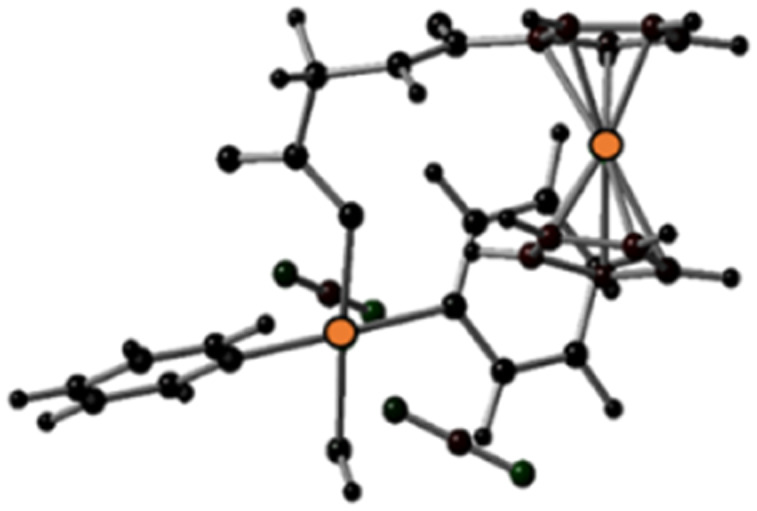	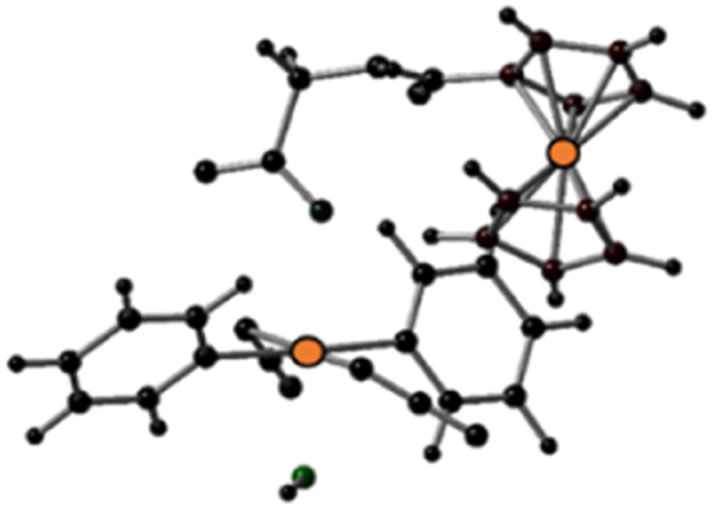
Bond length/Å	Pt–N1	2.104	2.098	2.544	2.112
	Pt–N2	2.097	2.104	2.584	2.105
	Pt–O1	2.079	2.081	2.085	2.588
	Pt–O2	2.011	2.012	2.023	2.280
Spin density SD	Pt	—	0	0.48	0.54
	Fe	—	1.96	1.22	1.28
Adiabatic energy gap Δ*E*/eV		0	0.85	0.48	1.84

**Fig. 5:**
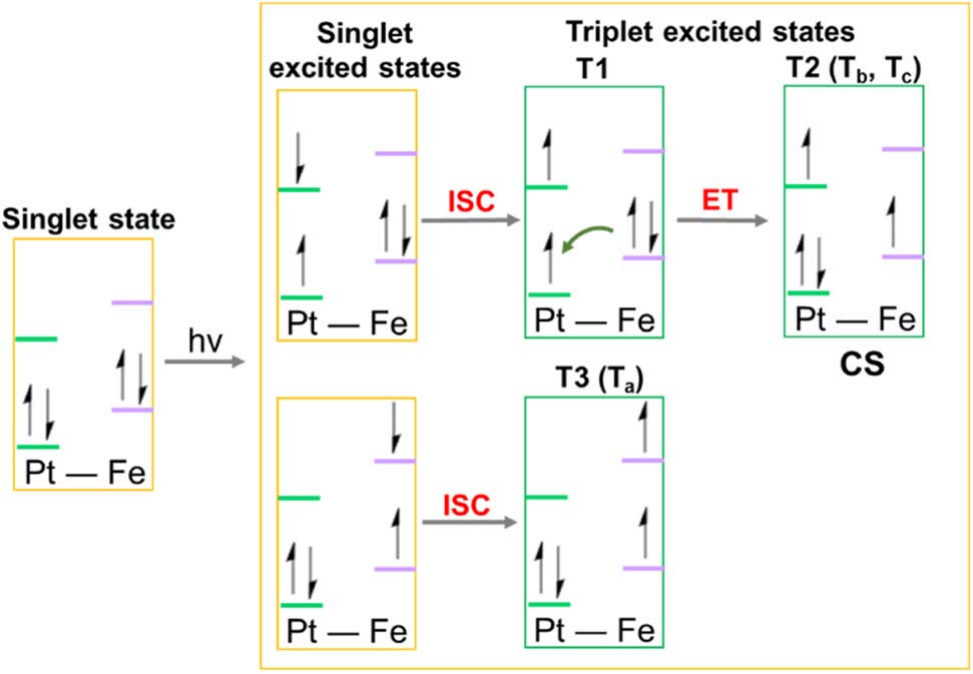
Schematic depiction of the steps, including an Electron Transfer (ET), leading to the formation of the triplet states potentially involved in the photoreactivity of Pt-Fe. ISC identifies the Intersystem Spin Crossing allowing the formation of triplet states, ET describes the Electron Transfer process that generates the Charge Separation (CS) state.

This analysis shows that both *T*_b_ and *T*_c_ optimised triplet states, that correspond to the triplet state indexed as T2 and representing a charge separation (CS) state in Fig. 5, could potentially be involved in ligand detachment. Upon irradiation, intersystem spin crossing (ISC) and population of such states, dissociation of both equatorial N_3_ and axial ligands can occur. The mechanism proposed to rationalise the formation of the triplet states showing CS is shown in Fig. 5. When Pt-Fe is irradiated, excited singlet states are generated. ISC allows the formation of triplet states centred on Fe (T1) and Pt (T3), while an Electron Transfer (ET) from iron to platinum occurs leading to the formation of Fe^3+^ and Pt^3+^ (T2). Ferrocene acts as a donor unit, while the platinum acts as an acceptor. The electronic transfer justifies the formation of the *T*_b_ and *T*_c_ triplet states. Of the described triplets, only that centred on Pt is involved in the dissociative process leading to the release of the axial ligands. TDDFT calculations confirm both the presence of weak transitions in the visible region of the spectrum originating from the presence of the ferrocene moiety, the electronic communication between the two subunits and the dissociative character of the excited states.

### 2.6. Photoreaction with 5′-GMP

The DNA base guanine is often a preferred target for platinum am(m)ine anticancer complexes. Therefore, the photoactivated interaction between Pt-Fe and 5′-GMP was investigated. An aqueous solution of Pt-Fe (30 μM) and 2 mol equiv. of 5′-GMP was irradiated with indigo (420 nm) or green (517 nm) light at 298 K for 1 h without prior incubation (Fig. S23, ESI†). The major Pt-GMP adducts detected by LC-MS were assigned as {Pt^II^(CH_3_CN)(py)_2_(GMP-H)}^+^ (755.69 *m*/*z*, A1), {Pt^II^(HCOO)(py)_2_(GMP)}^+^ (761.65 *m*/*z*, A2) and {Pt^II^(N_3_)(py)_2_(GMP)}^+^ (757.67 *m*/*z*, A3) after irradiation with indigo light (420 nm). However, with green light (517 nm) irradiation, the major adduct detected was {Pt^II^(N_3_)(py)_2_(GMP)}^+^ (757.66 *m*/*z*, A3), the other two adducts were too weak to be detected.

These products suggest that both azido and hydroxide ligands can act as electron donors in the photoreduction of Pt(IV) to Pt(II), with likely retention of the *trans*-{Pt(py)_2_}^2+^ fragment, and ready formation of the guanine adduct with the labile *trans* position being occupied by either CH_3_CN or formate from the HPLC eluent.

### 2.7. Photocytotoxicity

The photocytotoxicity of Pt-Fe towards human A2780 ovarian, A549 lung, PC3 prostate, T24 and SW780 bladder cancer cells, was investigated upon irradiation with blue (465 nm) and green light (520 nm) in comparison with parent complex 1 and cisplatin. The change in dose-dependent cell viability was determined by the sulforhodamine B (SRB) colorimetric assay and the results are summarised in Table 4.

**Table:** IC_50_ values and photocytotoxic indices (PI) for complex Pt-Fe in A2780 ovarian, A549 lung, PC3 prostate, T24 and SW-780 bladder cancer, and MRC-5 lung fibroblast cells after 1 h incubation, 1 h irradiation (blue 465 nm, green 520 nm) and 24 h recovery. CDDP (cisplatin) and complex 1 were studied for comparison

Cell			IC_50_^a^ (μM)		
			Pt–Fe	1 (ref. 53)	CDDP
A2780	Dark		>60	>100	>100
	465 nm (0.5 h)		15.4 ± 1.2	> 60	n.d.
	465 nm		1.5 ± 0.1	7.1 ± 0.4	>100
	520 nm		3.8 ± 0.3	>100	n.d.
	PI	Blue	>33	>14	—
		Green	>13	—	—
A549	Dark		>100	>100	>100
	465 nm		26.9 ± 0.9	51.9 ± 2.5	>100
	520 nm		41.3 ± 1.8	>100	n.d.
	PI	Blue	>3.7	>1.9	—
		Green	>2.4	—	—
PC3	Dark		>100	>100	>100
	465 nm		4.2 ± 0.4	55.6 ± 0.9	>100
	520 nm		9.0 ± 0.1	>100	n.d.
	PI	Blue	>24	>1.7	—
		Green	>11	—	—
T24	Dark		>100	>100	>100
	465 nm		7.5 ± 0.4	>100	>100
	520 nm		32.8 ± 1.6	n.d.	n.d.
	PI	Blue	>13	—	—
		Green	>3.0	—	—
SW780	Dark		>100	>100	>100
	465 nm		25.9 ± 2.5	>100	>100
	520 nm		41.9 ± 0.9	>100	>100
	PI	Blue	>3.8	—	—
		Green	>2.4	—	—
MRC5	Dark		>100	>100	>100

a Each value is mean of two independent experiments. n.d. = not determined.

Complex Pt-Fe had little effect on the viability of all cell lines in the dark (IC_50_ values > 60 μM), including the normal lung fibroblasts MRC5 cells. Upon irradiation with blue light (465 nm, 4.8 mW cm^−2^) for 1 h, the cytotoxicity of Pt-Fe increased significantly (IC_50_ = 1.5 μM for A2780, 26.9 μM for A549, 4.2 μM for PC3, 7.5 μM for T24, 25.9 μM for SW780), with photocytotoxic indices (PI = IC_50dark_/IC_50light_) towards cancer cells > 33 for A2780 ovarian, > 3.7 for A549 lung, > 24 for PC3 prostate > 13 for T24 bladder and > 3.8 for SW780 bladder (Table 4). In addition, the photocytotoxicity of Pt-Fe is *ca*. 5× higher than that of 1 in A2780 cells, *ca.* 2× more potent in A549 and *ca*. 13× in PC3 cells. Notably, parent complex 1 showed no photocytotoxicity towards bladder cancer T24 and SW780 cells, whereas significant micromolar activity was determined for Pt-Fe. For comparison, the clinically used drug cisplatin showed no apparent cytotoxicity (IC_50_ > 100 μM) towards all cell lines, attributable to the short incubation time. Even with a shorter irradiation time (30 min) in A2780 cells, Pt-Fe (IC_50_ 15.4 μM) was much more potent than 1 (IC_50_ > 60 μM).

The photocytotoxicity of Pt-Fe and 1 was also investigated using green light irradiation (520 nm, 11.7 mW cm^−2^, Table 4). Pt-Fe displayed remarkable photocytotoxicity (IC_50_ = 3.8 μM for A2780, 41.3 μM for A549, 9.0 μM for PC3, 32.8 μM for T24, 41.9 μM for SW780) with the photocytotoxicity indices (PI) towards cancer cells: > 13 for ovarian A2780, > 2.4 for lung A549, > 11 for prostate PC3, > 3.0 for bladder T24, and > 2.4 for SW780 cells. The photocytotoxicity of Pt-Fe with green light was *ca*. 2× lower than with blue light, but higher than those of 1 upon irradiation with blue light. In contrast, 1 exhibited no apparent cytotoxicity with green light irradiation (IC_50_ > 100 μM).

To mimic clinical conditions, the efficacy of Pt-Fe (Table S11, ESI†) towards human ovarian cancer A2780 and cisplatin-resistant A2780cis was also determined with longer post-treatment incubation (72 h) without drug removal under both normoxia (21% O_2_) and hypoxia (1% O_2_). Due to the long incubation time, Pt-Fe showed moderate dark cytotoxicity under normoxia (IC_50_ = 6.0 μM for A2780, and 8.7 μM for A2780cis) with *ca.* 7× and 1.5× enhanced photocytotoxicity in the presence of blue and green light, respectively. Although 1 and Pt-Fe showed similar blue light cytotoxicity towards A2780 cells, its blue light cytotoxicity towards A2780cis and green light cytotoxicity towards both cell lines were > 2× lower than that of Pt-Fe. No significant difference in the cytotoxicity of Pt-Fe was observed between A2780 and cisplatin-resistant A2780cis, whereas cisplatin showed *ca.* 10× decreased cytotoxicity towards A2780cis. Notably, the light itself had no apparent effect on the cytotoxicity of cisplatin.

Under hypoxia, Pt-Fe retained its photocytotoxicity towards A2780ovarian cancer cells and had *ca.* 1.5× enhanced photocytotoxicity towards resistant A2780cis, while its dark cytotoxicity decreased significantly. Complex 1 showed similar properties, but the cytotoxicity of cisplatin decreased by 10× and 1.6× towards A2780 and A2780cis, respectively.

### 2.8. Cellular accumulation

The cellular accumulation of Pt from Pt-Fe was determined for A2780 ovarian, A549 lung, PC3 prostate and T24 bladder cancer cells in comparison with that of complex 1. Cancer cells were treated with Pt(IV) complexes (10 μM) in the dark for 1 h (Table 5). Pt-Fe gives rise to 3–5× increased Pt accumulation compared with parent complex 1. However, when cells were treated with complexes at their blue light photo IC_50_ concentrations (1.5 μM for A2780, 26.9 μM for A549 and 4.2 μM for PC3) in the dark for 1 h, the cellular accumulation of Pt from 1 was similar to Pt-Fe (in A2780 and A549) or *ca.* 2× higher than Pt-Fe (in PC3, Table S12, ESI†). The cellular fraction of Pt-Fe (10 μM) in A549 cells after 1 h incubation indicated that the majority of Pt (88.9%) accumulated in the cell nuclei, 2.6% of the Pt localised in mitochondria, while 8.5% was in the cytoplasm (Fig. S24, ESI†).

**Table:** Accumulation of Pt (ng/10^6^ cells) in cancer cells after exposure to complexes 1 or Pt-Fe (10 μM, 1 h, in the dark)^a^

Complex	Platinum accumulation (ng/10^6^ cells)			
	A2780	A549	PC3	T24
Pt-Fe	3.4 ± 0.6**	4.9 ± 1.0*	6.7 ± 1.8*	3.8 ± 0.4***
1	1.19 ± 0.04***	1.2 ± 0.4*	1.3 ± 0.5*	n.d.

a All data were determined from triplicate samples and compared with values obtained for untreated cells using a two-tail *t*-test with unequal variances. *p < 0.05, **p < 0.01, ***p < 0.005.

Cellular accumulation of Pt-Fe and 1 in A2780 ovarian and T24 bladder cancer cells when treated at their IC_50_ concentrations were compared in the absence and presence of blue light (465 nm, Table S13, ESI†). Incubation with Pt(IV) complexes in the dark for 2 h resulted in accumulation of 2.0 and 0.9 ng Pt/10^6^ A2780 cells for complexes Pt-Fe and 1, respectively. The IC_50_ of Pt-Fe is *ca*. 5× lower than that for 1, and its Pt accumulation is *ca.* 2× higher than 1. After 1 h irradiation following 1 h incubation, Pt-Fe exhibited *ca.* 2× enhanced Pt accumulation of 3.7 ng Pt/10^6^ and 15.7 ng Pt/10^6^ for A2780 and T24 cells, respectively. Complex 1 also showed increased cellular Pt accumulation in A2780 cells after irradiation.

### 2.9. Singlet oxygen, hydrogen peroxide and radical generation in solution

**Fig. 6:**
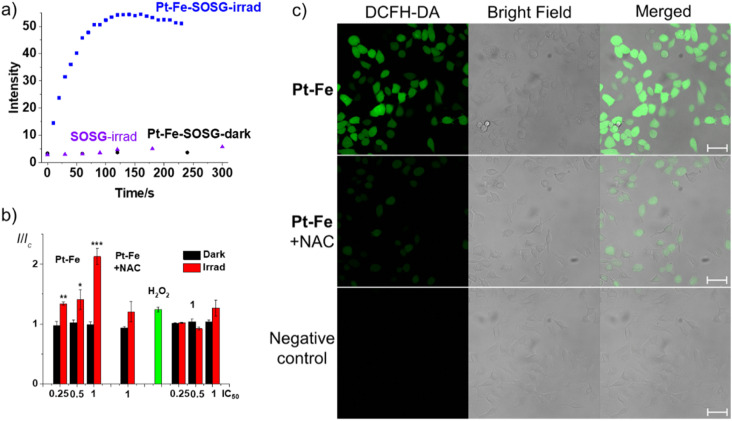
(a) Time dependent fluorescence (*λ*_ex_ = 504 nm, *λ*_em_ = 525 nm) of SOSG (1 μM) in an aqueous solution (with 5% DMSO and 1% MeOH v/v) containing Pt-Fe (50 μM) upon irradiation at 463 nm. The same solutions in the dark and SOSG alone upon irradiation were investigated for comparison; (b) relative fluorescence intensity of A549 cells treated with Pt-Fe (2 h or 1 h in the dark and 1 h irradiation, 465 nm) then probed with DCFH-DA (20 μM, *λ*_ex_ = 490 nm, *λ*_em_ = 510–570 nm). H_2_O_2_ (5 mM) was used as a positive control. The statistical significance between dark and irradiated samples were evaluated by a two-tail *t*-test with unequal variances. *p < 0.05, **p < 0.01, ***p < 0.005; (c) confocal fluorescence microscopy images of ROS generation of A549 cells treated with Pt-Fe (IC_50_ concentration, 1 h in dark and 1 h irradiation, 465 nm) then probed by DCFH-DA (20 μM, *λ*_ex_ = 488 nm, *λ*_em_ = 493–580 nm) in the absence and presence of antioxidant *N*-acetyl-l-cysteine (NAC, 10 mM). A549 cells irradiated without Pt-Fe were used as a negative control. Scale bar = 50 μm.

To determine the effect of a possible Fenton reaction from ferrocene, ^1^O_2_ was detected by monitoring the fluorescence of the ^1^O_2_ probe Singlet Oxygen Sensor Green (SOSG^®^), a probe which emits strong green fluorescence in the presence of ^1^O_2_ (*λ*_ex_ = 504 nm, *λ*_em_ = 525 nm). An aqueous solution of Pt-Fe (50 μM) and SOSG (1 μM) was irradiated with blue light (463 nm) and its fluorescence was measured every 10 s. The emission at 525 nm increased rapidly and reached a plateau after 90 s irradiation (Fig. 6a). No apparent change in the intensity of fluorescence was detected for the solution without Pt-Fe or irradiation.

To investigate possible hydrogen peroxide generation, 10 μL of each Pt(iv) complex (5 mM) in aqueous solution was dropped on a peroxide test stick (Quantofix^®^ Peroxide 25, Fig. S25, ESI†).

No colour change was observed for both complexes in the dark, but the area stained with 1 turned blue after 30 s irradiation with blue light (463 nm), and the H_2_O_2_ concentration was semi-quantitatively measured as 5 mg L^−1^ by comparison with the colour scale. However, no formation of hydrogen peroxide was determined when the test stick was treated with Pt-Fe; the stick remained colourless even after irradiation, suggesting that the complex was able to catalyse decomposition of hydrogen peroxide.

To further investigate the ROS generated in the photodecomposition of Pt-Fe, EPR spectra were recorded using DMPO as a spin trap (Fig. S26, ESI†). A mixture of 2.5 mM Pt-Fe and 10 mM DMPO in RPMI-1640 without phenol red was irradiated continuously by blue light (463 nm) and monitored by EPR. Spin-trap adducts with azidyl radicals DMPO–N_3˙_ and hydroxyl radicals DMPO–OH˙ were detected as a 1 : 2 : 2 : 1 quartet of triplets and a 1 : 2 : 2 : 1 quartet, respectively. Parameters used for simulation were in good agreement with data reported previously.^54^

### 2.10. Cellular ROS generation

Cumulative ROS can result in cellular damage (oxidative stress), which can lead to cell death.^55,56^ The ROS level of A549 cells was determined using the DCFH-DA assay with and without irradiation with blue light (465 nm). A549 cells were seeded in 96-well plates and exposed to Pt-Fe or 1 for 1 h at 310 K in the dark at concentrations of 26.9 (IC_50_), 13.5 (0.5 × IC_50_), and 6.8 (0.25 × IC_50_) μM, where IC_50_ refers to the half inhibitory concentration of Pt-Fe with 1 h of blue light irradiation. One plate was kept in the dark, while the other was irradiated with blue light for 1 h. Complexes were removed and DCFH-DA (20 μM) was added and incubated with cells for 40 min. Cells were then washed with HBBS and the fluorescence intensity was measured in colourless serum-free RPMI medium. A significant increase in fluorescence was observed for irradiated cells treated with Pt-Fe, which increased with drug concentration (Fig. 6b). For cells treated with Pt-Fe at IC_50_ value, a 2× enhanced fluorescence was observed compared to untreated cells, which was partially quenched to 1.2× by the ROS scavenger *N*-acetyl-l-cysteine (NAC, 10 mM). In contrast, for the dark plates, the fluorescence intensity of cells treated with Pt-Fe was similar to the control cells. A less significant fluorescence increase (1.3×) was observed for cells treated with 1 at same concentration upon irradiation (Fig. 6b).

The oxidation-induced DCFH-DA fluorescence was investigated using confocal microscopy (LSM 880, AxioObserver). A549 cells were seeded in cell culture dishes with glass bottoms. Cells were incubated with Pt-Fe (26.9 μM, IC_50_) for 1 h, irradiated with blue light for 1 h, then exposed to DCFH-DA (20 μM) for 40 min. Green fluorescence (*λ*_ex_ = 488 nm, *λ*_em_ = 493–580 nm), was evident in treated cells, indicating the presence of ROS (Fig. 6c). In addition, cells treated with Pt-Fe (26.9 μM, 1× IC_50_) and light in the presence of the ROS inhibitor NAC (10 mM) only exhibited weak green fluorescence under the same excitation conditions.

In comparison, no apparent fluorescence was observed in cells treated with light in the absence of Pt-Fe, suggesting a lower ROS level. Notably, cells treated with Pt-Fe and light exhibited cellular morphological changes, including cellular shrinkage, loss of adhesion, and membrane disintegration, while cells treated with light only maintained a good cellular morphology and remained well attached to the culture dish.

### 2.11. Cancer cell death mechanism

Ferroptosis is an iron-dependent cell death mechanism involving accumulation of lethal ROS, which leads to lipid peroxidation.^57^ A549 cells were seeded in 6-well plates and exposed to Pt-Fe at 0.5× or 1× IC_50_ for 1 h at 310 K in the dark, followed by blue light (465 nm) irradiation for 1 h. After staining with BODIPY™ 581/591C11 (5 μM, a lipid peroxidation sensor) for 30 min, A549 cells were collected and analysed using a BD LSR II flow cytometer (Fig. S27, ESI†). Significant enhancement in the fluorescence at 500–560 nm (*λ*_ex_ = 488 nm) was observed for cells treated with Pt-Fe and irradiation, indicating lipid peroxidation. In contrast, cells treated with Pt-Fe in the dark showed no difference from the untreated control and irradiation alone which showed only a slight increase in the fluorescence. The GSH and GXP4 levels in A549 cells were also investigated to confirm ferroptosis induced by treatment with irradiated Pt-Fe (Fig. S28, ESI†). A549 cells exposed to Pt-Fe at 1× IC_50_ for 1 h at 310 K in the dark, followed by blue light (465 nm) irradiation for 1 h displayed a reduced GSH level (*ca.* 50%) compared with cells exposed to Pt-Fe without irradiation and the untreated control (Fig. S28a, ESI†). In addition, a decreased level of GPX4 (*ca.* 60%) was observed A549 cells treated with irradiated Pt-Fe (1× IC_50_) using blue light (465 nm), compared with untreated control and A549 cells treated with Pt-Fe (1× IC_50_) in the dark (Fig. S28b, ESI†).

The possible contribution of apoptosis to the death of A549 cells induced by Pt-Fe was also explored, since apoptosis is generally accepted as the mechanism of cell death for cisplatin. Annexin V labelled with green fluorescent FITC binds to phosphatidylserine exposed on the outer membrane of apoptotic cells. However, when A549 cells were treated with Pt-Fe for 1 h then 465 nm irradiation for 1 h, and stained with Annexin V-FITC/PI, no apparent difference was observed between untreated control and cells treated with Pt-Fe and irradiation (Fig. S29, ESI†).

To exclude the effect of incubation time, apoptosis in A2780 and cisplatin-resistant A2780cis cells induced by Pt-Fe (IC_50_ concentration) was also explored (Fig. S30 and S31, ESI†). When these two cell lines treated with Pt-Fe with 1 h incubation, 1 h irradiation (465 nm), and followed by 72 h further incubation under both normoxia and hypoxia, then stained with Annexin V-FITC/PI, they showed no apparent difference compared with the untreated control.

## 3. Discussion

Phototherapy using metal complexes is attractive for the design of novel anticancer agents with new mechanisms of action and fewer side effects. The choice of the metal, its oxidation state, types and number of coordinated ligands and coordination geometry can all be used to tune photochemical and photobiological activity, an area of study still in its infancy.^5^ Here we have modified a parent photochemotherapeutic agent, the octahedral diazido Pt(iv) complex 1, by conjugation of an axial ligand to ferrocene as a light antenna with the aim of increasing the wavelength of activation (and increased light penetration into tissues) and introducing an additional mechanism of biological action based on redox reactions of ferrocene. In particular, we have carried out detailed DFT and TDDFT calculations on the conjugate Pt-Fe to investigate the effect of the heterobimetallic system on electronic transitions, and intersystem crossing to produce triplet states, and possible Pt(iv)–Fe(ii) communication.

### 3.1. Synthesis and structure

Complex Pt-Fe was prepared *via* a 3-step synthetic route from parent complex 1 with a satisfactory yield. Its structure was confirmed by X-ray crystallography, NMR, HR-MS and elemental analysis. A distance of 6.070 Å between the Pt and Fe centres was observed in the X-ray crystal structure. Any communication between Pt and Fe must occur through an incompletely conjugated π system.

### 3.2. Absorption spectrum and cyclic voltammetry

A long low-energy tail in the 400–500 nm range was observed in both experimental and calculated absorption spectra of Pt-Fe. The transition can be described as metal-to-metal charge-transfer (MMCT), where the CT occurs from the ferrocene Fe atom to the Pt centre. Compared with Fc and its derivative Fc-COOH, the absorption of Pt-Fe in this range displays a 2-3× enhanced intensity. The absorbance at 293 nm for 1 red-shifted to 299 nm for Pt-Fe. This transition has a preponderant LMCT character from the axial ferrocene ligand to Pt. A significantly enhanced absorbance at 260 nm was detected and computations assign LLCT character, involving ferrocene, to the excitation in this region.

The covalently attached electron-withdrawing Pt(iv) group shifts the reversible Fc^+^/Fc oxidation wave of Pt-Fe to higher potential. The less negative irreversible reduction wave assigned to Pt^IV^/Pt^II^ of Pt-Fe suggests more facile reduction to Pt(ii) compared with the parent complex. Communication between the two metal centres is therefore evident even with their 6.070 Å separation and lack of complete electronic conjugation. Similar increases in oxidation potential for the ferrocene fragment have been observed for complexes with an appended Pt(ii) fragment containing an electron-withdrawing terpyridyl ligand.^41^

### 3.3. Photoactivation and photoreaction

Upon irradiation, ISC is driven by large computed SOC values between the singlet state S11 excited by 410 nm light and T12, T13 and T15, with the ^3^MMCT nature all involving charge transfer from the Fe to the Pt. The large SOC values are attributable to the presence of the heavy platinum atom. In contrast, the S25 excited state generated by 331 nm irradiation shows LMCT character and can undergo ISC to T23, T24, T26 and T27 triplet states with ^3^LMCT character, involving charge transfer between several ligands and the Pt atom. This is ascribed as the origin of the photodissociation of ligands from Pt. TDDFT calculations on triplet states confirmed that the ISC allows the formation of triplet states centred on Pt and Fe, while an electron transfer from iron to platinum can occur with the formation of Fe^3+^ and Pt^3+^.

Pt-Fe undergoes photoactivation at *a* >2× higher rate compared with parent complex 1, especially with green light (517 nm), due to the presence of the electron donating Fc moiety. The photodecomposition mechanism of Pt-Fe is very complicated, and includes the release of one and two azide ligands and axial ligands (Fig. S32, ESI†). Photo-reduced Pt(ii) species were detected as products by LC-MS (Table S5, ESI†). Azidyl and hydroxyl radicals and gly-Fc ligand are released during photoactivation, owing to the LMCT charge transfer involving Pt and ligands. The release of cyclopenta-1,3-diene from Fc moiety can also be observed in LC-MS despite the low ratio. The enhanced absorption in the range of 400–600 nm matches well with the UV-vis spectral change during photo-aquation of ferrocenyl derivatives.^58^ The Pt(ii) photoproducts can form adducts with 5′-GMP, indicating they are likely to bind strongly to DNA. The combined mechanisms strongly suggest that Pt-Fe can kill cancer cells in a different way from cisplatin. The high dark stability of Pt-Fe in cell culture media and in the presence of bio-reductants, suggest likely high photo-selectivity for this complex. Notably, the photoactivation of Pt-Fe is little affected by oxygen concentration, which suggests an ability to retain photocytotoxicity under hypoxia.

### 3.4. Photocytotoxicity and cellular accumulation

Complex Pt-Fe displays low dark cytotoxicity and promising photocytotoxicity with visible light (465 and 520 nm) in a wide range of cancer cells after 1 h incubation, 1 h irradiation and 24 h recovery. Since the Pt-Fe solution was removed and cells were washed after 2 h incubation for the dark cytotoxicity test, the small amount of reduced Pt-Fe exerted no apparent damage to the cells. Notably, no apparent photocytotoxicity under green light exposure was detected for 1, while Pt-Fe shows low IC_50_ values under the same conditions. This matches well with the red-shifted absorption induced by ferrocene conjugation, with enhanced photoactivation driven by MMCT from Fe to Pt, and the stronger photooxidant activity of Pt-Fe.

Other than behaving as a light antenna, conjugation with ferrocene improves the lipophilicity of Pt-Fe compared to 1. Irradiation can significantly enhance the Pt uptake within cancer cells. Azidyl radicals generated from Pt-Fe upon irradiation can attack cell membranes, so facilitating uptake. In addition, reduction of Pt(iv) produces more reactive Pt(ii) species which can bind to intracellular sites and enhance retention.

Notably, Pt-Fe exhibits similar cytotoxicity towards A2780 and cisplatin resistant A2780cis ovarian cancer cells under both normoxia and hypoxia when cells are treated under the clinically relevant protocol of 1 h incubation, 1 h irradiation, and 72 h further incubation without drug removal. Due to the longer incubation time, dark cytotoxicity was observed for Pt-Fe, which matches well with the reduction of Pt(iv)in the reductive intracellular environment after long incubation times. However, enhancement in cytotoxicity was still observed upon photoactivation. In addition, the ability to overcome cisplatin and hypoxia resistance suggests a novel mechanism of action compared with clinically drug cisplatin and PDT photosensitisers.

Enhanced cellular accumulation of Pt was observed for Pt-Fe compared with parent complex 1 when treated with low concentrations. Surprisingly, 88.9% of the Pt accumulated in nuclei before irradiation, in contrast to 1 that mainly localised in the cytoplasm in the dark.^59^ Since DNA appears to be a major target for these diazido Pt complexes, high accumulation of Pt in the nuclei can contribute the high photocytotoxicity of Pt-Fe.

### 3.5. ROS generation and mechanism of action

Generation of ROS, *e.g.*, hydroxyl radicals and singlet oxygen from Pt-Fe upon irradiation was detected in both aqueous solution and cancer cells. No H_2_O_2_ generation was observed for irradiated Pt-Fe, while it can be produced by 1. The role of ferrocene involves a Fenton-type pathway as evidenced by the lack of detection of H_2_O_2_ formation, the decomposition of H_2_O_2_ being catalysed by Fe(ii). This is consistent with the *ca.* 2× higher ROS level determined for cancer cells treated with Pt-Fe compared to 1.

The large amount ROS produced by Pt-Fe seems to play an important role in its mechanism of action. Ferroptosis, characterised by lipid peroxidation, involving the key roles of iron and ROS, appeared to be the main mechanism of cell death induced by Pt-Fe. Decreased GSH and GPX4 levels were observed. In contrast, apoptosis that is traditionally the mechanism of cell death for cisplatin was not observed. Thus, Pt-Fe exhibits similar photocytotoxicity towards wild type and cisplatin-resistant ovarian cancer cells. In addition, since ROS kills cancer cells more rapidly than Pt(ii) species, the improvement in photocytotoxicity by introduction of ferrocene using a short 24 h recovery was more significant than using the longer 72 h further incubation.

## 4. Conclusions

A novel hetero dinuclear complex, Pt-Fe, containing both diazido Pt(iv) and Fe(ii) ferrocene fragments has been synthesised and characterised, with its crystal structure determined by X-ray diffraction. The conjugation of ferrocene with the Pt(iv) complex contributes to the improvement of several properties of Pt-Fe. Specifically, (a) the absorption spectrum is red-shifted and the ferrocene moiety behaves as a light antenna to allow charge transfer from iron to platinum, thereby promoting the photoactivation of Pt-Fe with light at longer wavelength; (b) cellular accumulation is enhanced; (c) ROS generation is catalysed after irradiation inducing ferroptosis as a cell-death mechanism.

Compared to the parent Pt complex 1, Pt-Fe shows a broad absorbance band at 435 nm and a less negative irreversible reduction wave for Pt^IV^/Pt^II^, together with high dark stability and photoactivation when irradiated with blue, green, and orange light generating cytotoxic oxygen independent species, in contrast to conventional PDT photosensitisers. Upon irradiation, Pt-Fe can bind to the DNA base guanine. Significantly improved photocytotoxicity towards a number of cancer cell lines was observed for Pt-Fe when irradiated with blue and green light with high photocytotoxicity indices. Notably, similar photocytotoxicity towards A2780 and cisplatin-resistant A2780cis was found for Pt-Fe under both normoxia and hypoxia. Pt-Fe also shows increased Pt cellular accumulation compared with 1, which appears to be enhanced by irradiation. The ROS level in cancer cells treated with Pt-Fe and irradiation increased markedly, driven by Fenton-type catalysis due to the appended ferrocene. As a result, ferroptosis rather than apoptosis was the major mechanism of cell death induced by Pt-Fe after irradiation.

DFT computations demonstrated that not only do the iron and platinum subunits in Pt-Fe communicate, but the ferrocene is able to act as donor towards the acceptor Pt(iv) fragment leading to the formation of charge-transfer triplets. This finding will guide future work to produce more efficient prodrugs and novel dyad systems by conjugating Pt(iv) photoactivatable drugs and ferrocene donors. In particular, the nature and the distance of the spacer, the electron mediator, between the donor–acceptor units, will be varied to modulate the photochemical properties and the photoinduced ET process will be investigated in depth.

In conclusion, the novel platinum–iron complex Pt-Fe with its charge transfer between two metal centres, visible light activation, promising photocytotoxicity, ability to overcome cisplatin and hypoxia resistance, and unique mechanism of action, is a promising photoactive prodrug for cancer therapy and will inspire the design of next-generation phototherapeutic agents.

## Data availability

Data are available in the ESI† and from the authors on request.

## Author contributions

HS designed, synthesised and characterised the complex, performed photochemical and photobiological experiments. PJS supervised the research. FP carried out DFT and TDDFT calculations. JSG and RD determined the expression of GPX4. GC carried out X-ray crystallography. CI and IHP contributed to confocal microscopy. HS, FP, ES and PJS analysed and interpreted the results and drafted the script. All authors contributed to the final submission.

## Conflicts of interest

There are no conflicts to declare.

## Supplementary Material

SC-015-D3SC03092J-s001

SC-015-D3SC03092J-s002
