# Supplementary material for: Tuning the photoactivated anticancer activity of Pt(iv) compounds via distant ferrocene conjugation
Source: Chem Sci. 2024 Feb 14;15(11):4121–34. doi: 10.1039/d3sc03092j (PMC10935708; doi:10.1039/d3sc03092j)
Supplement: SC-015-D3SC03092J-s001 [file SC-015-D3SC03092J-s001.pdf]

Electronic Supplementary Material (ESI) for Chemical Science.
This journal is © The Royal Society of Chemistry 2024

Tuning the photoactivated anticancer activity of Pt(IV) compounds via distant ferrocene conjugation

Huayun Shi,[†,a] Fortuna Ponte,[†,b] Jaspreet S Grewal,[c] Guy J Clarkson,[a] Cinzia Imberti,[a] Ian Hands-Portman,[d] Robert Dallmann,[c] Emilia Sicilia,*[b] Peter J Sadler*[a]

[a] Department of Chemistry, University of Warwick, Coventry CV4 7AL, UK

[b] Department of Chemistry and Chemical Technologies, University of Calabria, via Pietro Bucci, 87036 Arcavacata di Rende, Cs, Italy

[c] Division of Biomedical Sciences, Warwick Medical School, University of Warwick, Coventry CV4 7AL, UK.

[d] School of Life Sciences, University of Warwick, Coventry CV4 7AL, UK.

Electronic Supporting Information

Contents

Experimental Section 4

Table S1. UV-vis absorption spectra in various solvents at 298 K. 14

Table S2. Crystallographic data and structure refinement for **Pt-Fe**. 15

Table S3. Selected bond lengths (Å) and bond angles (°) for **Pt-Fe**. 16

Table S4. Selected hydrogen bond parameters for **Pt-Fe**. 16

Table S5. Photochemical decomposition products (420 nm irradiation) from complex **Pt-Fe** detected by LC-MS. 17

Table S6. Photochemical decomposition products (420 nm irradiation) from complex **1** detected by LC-MS. 17

Table S7. Photochemical decomposition products (517 or 593 nm irradiation) from complex **Pt-Fe** detected by LC-MS. 18

Table S8. TD-DFT benchmark for **Pt-Fe** on the structure optimised at B3LYP-D3/6-31G* level in DMSO implicit solvent. 19

Table S9. Excitation energies (ΔE, eV), absorption wavelength (λ, nm), oscillator strength (f), MO contribution (%) for selected transitions (Tr) for **Pt-Fe**. 20

Table S10. Excitation energies (ΔE, eV), absorption wavelength (λ, nm) and MO contribution (%) for **Pt-Fe** selected triplet states. 21

Table S11. IC_{50} values and photocytotoxicity indices (PI) for complex **Pt-Fe** in A2780 and cisplatin-resistant A2780cis ovarian cancer cells after 1 h incubation, 1 h irradiation (blue 465 nm, green 520 nm) and 72 h incubation after treatment. 22

Table S12. Accumulation of Pt (ng/10^6 cells) in cancer cells after exposure to complexes **1** and **Pt-Fe** (equipotent IC_{50} concentrations, 1 h, in the dark).................23

Table S13. Cellular accumulation of Pt (ng/10^6 cells) in cancer cell after exposure to complexes **1** and **Pt-Fe** (equipotent IC_{50} concentrations)..23

Figure S1. HPLC UV chromatogram for **Pt-Fe** confirming its purity.......................24

Figure S2. HR-MS of **Pt-Fe** at 298 K...24

Figure S3. 400 Hz ^{1}H NMR of **Pt-Fe** in DMSO-d_6 at 298 K. 25

Figure S4. 125 Hz ^{13}C NMR ATP spectrum of **Pt-Fe** in DMSO-d_6 at 298 K. 25

Figure S5. UV-vis spectra of complexes in a) H_2O, b) PBS, c) DMSO at 298 K. 26

Figure S6. a) The intramolecular distance between Pt and Fe, b) the shortest distance between pyridine and ferrocene protons, c) hydrogen bonds, and d) crystal packing for **Pt-Fe**. 26

Figure S7. Cyclic voltammogram of **Pt-Fe** and ferrocene..26

Figure S8. HPLC UV chromatogram for **Pt-Fe** (50 μM) in aqueous solution after incubation in the dark at 310 K for 0, 2 and 24 h. 27

Figure S9. UV-vis spectral changes for **Pt-Fe** (50 μM) in a) air b) N_2-saturated PBS with 5% DMSO in the presence of 50 μM ascorbic acid in the dark at 310 K for 120 min. 27

Figure S10. HPLC UV chromatogram for **Pt-Fe** (50 μM) in aqueous solution after incubation in the presence of 50 μM ascorbic acid in the dark at 310 K for 0, 2 and 24 h...27

Figure S11. HPLC UV chromatogram for **Pt-Fe** (50 μM) in aqueous solution after incubation in the presence of 2 mM GSH in the dark at 310 K for 0 and 24 h. 28

Figure S12. UV-vis spectral changes for **Pt-Fe** (50 μM, N_2-saturated phenol red-free RPMI-1640 with 5% DMSO) upon blue (a, 463 nm, 60 min), green (b, 517 nm, 90 min) or orange (c, 593 nm, 90 min) light irradiation...28

Figure S13. a) UV-vis spectral changes for **1** in phenol red-free cell culture medium RPMI-1640 with 5% DMSO with green light (517 nm); and b) time-dependent absorbance changes of **Pt-Fe** and **1** at 300 nm upon irradiation with green light (517 nm). 28

Figure S14. Photochemical decomposition of **Pt-Fe** determined by HPLC after irradiation with indigo light (420 nm). 29

Figure S15. Photochemical decomposition of **1** determined by HPLC after irradiation with indigo light (420 nm). 29

Figure S16. Photochemical decomposition of **Pt-Fe** determined by HPLC after 0 and 60 min irradiation with green light (517 nm)..30

Figure S17. Photochemical decomposition of **Pt-Fe** determined by HPLC after 0 and 60 min irradiation with orange light (593 nm)...30

Figure S18. B3LYP optimised geometrical structure of **Pt-Fe**. 31

Figure S19. NTOs for transitions labelled 1-21 for **Pt-Fe**. 33

Figure S20. NTOs for **Pt-Fe** triplet states lying below the bright singlet state and the Tr7 transition. 35

Figure S21. Optimised structures of excited triplet states of **Pt-Fe** obtained at the TD-DFT B3LYP/SDD/6-31G* level of theory. 35

Figure S22. Spin density distributions of the optimised structures of excited triplet states of **Pt-Fe** obtained at the TD-DFT B3LYP/SDD/6-31G* level of theory. 36

Figure S23. Photoreaction between **Pt-Fe** and 5′-GMP in aqueous solution at 298 K after irradiation monitored by HPLC. 36

Figure S24. ICP-MS quantification of Pt distribution in A549 cells. Cells were treated with 10 μM **Pt-Fe** at 310 K for 1 h in the dark. 37

Figure S25. Colour change for aqueous solutions of complex **1** or **Pt-Fe** (5 mM) in the stained area on the paper zone of the Peroxide 25 Test Sticks (Quantofix®) in the absence or presence of irradiation. 37

Figure S26. Observed (black) and simulated (red) EPR spectra of complex **Pt-Fe** (2.5 mM) in RPMI-1640 cell culture solution (containing 5% DMSO) showing the formation of DMPO−N_3• and DMPO−OH• adducts after irradiation (465 nm). 38

Figure S27. Lipid peroxidation assay of A549 cells stained by BODIPY™ 581/591 C11 ($\lambda_{ex}/\lambda_{em}$ = 488/500–560 nm) and analysed by flow cytometry. 38

Figure S28. (a) Cellular GSH level determined in A549 cells after treatment with **Pt-Fe** (IC_{50}) in the dark or irradiated with blue light; (b) expression of GPX4 in A549 cells after treatment with **Pt-Fe** (IC_{50}) in the dark or irradiated with blue light. 39

Figure S29. Cell apoptosis assay for A549 cells double stained by Annexin V-FITC/PI ($\lambda_{ex}/\lambda_{em}$ = 488/500–560 nm for Annexin V-FITC, $\lambda_{ex}/\lambda_{em}$ = 488/645–735 nm for PI) and analysed by flow cytometry. 39

Figure S30. Cell apoptosis assay of A2780 and A2780cis cells under normoxia double stained by Annexin V-FITC/PI ($\lambda_{ex}/\lambda_{em}$ = 488/500–560 nm for Annexin V-FITC, $\lambda_{ex}/\lambda_{em}$ = 488/645–735 nm for PI) and analysed by flow cytometry. 40

Figure S31. Cell apoptosis assay of A2780 and A2780cis cells under hypoxia double stained by Annexin V-FITC/PI ($\lambda_{ex}/\lambda_{em}$ = 488/500–560 nm for Annexin V-FITC, $\lambda_{ex}/\lambda_{em}$ = 488/645–735 nm for PI) and analysed by flow cytometry. 40

Figure S32. Possible photodecomposition pathways for **Pt-Fe** in aqueous solution on irradiation with indigo light (420 nm), as indicated by fragments observed in mass spectra. 41

Experimental Section

Materials and Instruments. *O*-(Benzotriazol-1-yl)-*N*, *N*, *N*’, *N*’-tetramethyluronium tetrafluoroborate (TBTU) was purchased from Merck, pyridine from Fischer Scientific UK, K_2PtCl_4, NaN_3, H_2O_2 (30%), Fmoc-gly-OH, *N*, *N*-diisopropylethylamine (DIPEA), piperdine, ferrocenoyl chloride and other chemicals from Sigma Aldrich and used without further purification.

NMR spectra were recorded on Bruker Avance III HD 400 MHz and Bruker Avance III HD 500 MHz spectrometers; the residual signal of the solvent was used as a reference.

ESI-MS spectra were recorded on an Agilent 6130B single quadrupole detector instrument at 298 K with a scan range of m/z 50-2000 for positive ions and HRMS data were collected on a Bruker microTOF instrument at 298 K.

Electronic absorption spectra were recorded on a Varian Cary 300 UV-vis spectrophotometer in a quartz cuvette and solvent used as reference. The spectral width was 200–600 nm, bandwidth 1.0 nm, scan rate 600 nm/min. A Jasco FP-6500 Spectrofluorometer was used to record fluorescence spectra.

LC-MS was carried out on a Bruker Amazon X mass spectrometer connected online to an Agilent 1260 HPLC with an Agilent ZORBAX Eclipse XDB-C18 column (250×4.6 mm, 5 μm, flow rate: 1 mL/min), using linear gradients of 0.1% FA in H_2O (solvent A) and 0.1% FA in CH_3CN (solvent B, 10%–80% in 30 min).

The light sources used for photoactivation were an LZC-ICH2 photoreactor (Luzchem Research Inc.) equipped with a temperature controller and 8 Luzchem LZC-420 lamps without light filtration, and LED light sources (BASETech model no. SP-GU10 230 V~50 Hz 1.3-2.1 W) with λ_{max} = 463, 517 or 593 nm. A 96-array of LEDs with λ_{max} = 465 (4.8 mW cm^{-2} per LED) or 520 (11.7 mW cm^{-2} per LED) nm was used for *in vitro* growth inhibition. A Whitley miniMACS Anaerobic Workstation in combination with a BOC gas cylinder (1% O_2/5% CO_2/94% N_2, 200 bar) was used to provide an hypoxia environment for *in vitro* cell growth studies.

Platinum contents were analysed on an ICP-MS 7900 Series (Agilent) or ICP-OES 5300DV (Perkin Elmer). The emission wavelength used for detection of Pt in ICP-OES was 265.945 nm.

Synthesis and characterisation. *Caution!* Due care and attention with appropriate precautions should be taken in the synthesis and handling of shock-sensitive heavy metal azides in the dark even though no problem was encountered during the work reported here.

***Trans, trans, trans*-[Pt(py)$_2$(N$_3$)$_2$(OH)(gly-Fc)]** (**Pt-Fe**). ***Trans, trans, trans*-[Pt(py)$_2$(N$_3$)$_2$(OH)(gly)]** (**2**, 50 mg, 95 μmol) and ferrocenoyl chloride (28.2 mg, 113 μmol) were suspended in DCM and DIPEA (100 μL) was added. The reaction mixture was stirred overnight at 298 K under a nitrogen atmosphere. After evaporation to dryness, the oily residue was collected and purified by column chromatography on silica gel (6% methanol + 94% DCM). Yield: 24%. ^{1}H NMR (DMSO-d_6, 400 MHz): 8.86 (d with Pt satellites, J = 5.34 Hz, J ^{195}Pt-^{1}H = 26.15 Hz, 4H, H_α *py*), 8.29 (t, J = 7.63 Hz, 2H, H_γ *py*), 8.02 (s, 1 H, *CONH*), 7.85 (t, J = 7.12 Hz, 4H, H_β *py*), 4.79 (t, J = 1.89 Hz, 2H, *CH*), 4.32 (t, J = 1.87 Hz, 2H, *CH*), 4.13 (s, 5H, *CH*), 3.89 (s, 1H, *OH*), 3.86 (d, J = 5.97 Hz, 2H, CH_2). ^{13}C NMR (DMSO-d_6, 125 MHz): 173.27 (*COO*), 169.68 (*CONH*), 149.85 (C_α *py*), 142.46 (C_γ *py*), 126.72 (C_β *py*), 76.80 (*C*), 70.27 (*CH*), 69.79 (*CH*), 68.57 (*CH*), 43.24 (CH_2). ESI-HR-MS: [M + Na]$^+$ (*m/z*) Calcd., 763.0764; Found, 763.0758. Anal. Calcd.: $C_{23}H_{23}FeN_9O_4Pt$: C 37.31, H 3.13, N 17.03. Found: C 37.28, H 3.04, N 17.00.

X-Ray crystallography. Single crystals of **Pt-Fe** were grown from DCM+MeOH/Et$_2$O. A suitable crystal was selected and mounted on a glass fibre with Fomblin oil and placed on a Rigaku Oxford Diffraction SuperNova diffractometer with a dual source (Cu at zero) equipped with an AtlasS2 CCD area detector. The crystal was kept at 150(2) K during data collection. Using Olex2[1], the structure was solved with the ShelXT[2] structure solution program using Intrinsic Phasing and refined with the ShelXL[3] refinement package using Least Squares minimisation.

Dark stability and photodecomposition in solution. The dark stability and photodecomposition of complex **Pt-Fe** (50 μM) in air or N$_2$ saturated RPMI-1640 without phenol red was monitored by UV-vis spectroscopy. The dark stability in aqueous solution was monitored by LC-MS at different time intervals alone, or in the presence of 2 mM GSH or 50 μM after ascorbic acid at 310 K. The photoactivation in aqueous solution was determined by LC-MS at different time intervals upon irradiation with indigo (420 nm), blue (463 nm), green (517 nm), and yellow (598 nm) light at 298

K. The air or N_2 saturated phosphate-buffered saline (PBS) solution of complex **Pt-Fe** (50 μM) and ascorbic acid (50 μM) were kept in the dark at 310 K and monitored by UV-vis to test the dark stability.

Electrochemistry. All cyclic voltammogram (CV) experiments were carried out using a CH Instrument model 600D Electrochemical Analyzer/Workstation (Austin, TX). The **Pt-Fe** solution was prepared in DMF containing tetrabutylammonium hexafluorophosphate (0.1 M) as supporting electrolyte and degassed under nitrogen. A typical three-electrode system was used to scan the cyclic voltammograms: a glassy carbon electrode as the working electrode, Ag/$AgNO_3$ (10 mM in DMF) as the reference electrode, and platinum wire as the counter electrode. The scan rate was set as 100 mV/s.

DFT Calculations. All the calculations were carried out with Gaussian 16[4] suite of programs employing DFT and time-dependent DFT methods.

Ground singlet state optimization for **Pt-Fe** was performed by using the B3LYP functional[5,6] with Grimme's D3 dispersion correction[7] and adopting the SMD implicit solvatation model[8] to simulate water as solvent ($\varepsilon = 80$). For the Pt atom, the relativistic compact Stuttgart/Dresden effective core potential was used[9] and the corresponding valence basis set was selected. The standard 6-31G* basis set was used for all other atoms.

The time-dependent DFT approach was applied at the same level of theory on the ground state structure of the system under investigation to determine vertical excitation energies and simulate the optical absorption spectra in dimethyl sulfoxide and water solvents.

This protocol was selected for testing the performance of different DFT functionals through the comparison of some important calculated absorption wavelengths with the corresponding experimentally detected for the **Pt-Fe** system in DMSO solvent. B3LYP-D3,[6,7] B3PW91,[5,10,11] CAM-B3LYP,[12] TPSS,[13] PBE,[14] PBE0,[15] M05,[16] M06,[17] M06L,[18] M062X,[18] M11,[19] MN12L,[20] were used for this purpose and B3LYP-D3 was, accordingly, selected.

To establish the probability that a triplet state could be populated and ISC processes occur, the spin orbit matrix elements for the coupling of the states potentially involved

were calculated at the ground state optimized geometry of the investigated systems using ORCA quantum chemistry program[21,22] and SOC values were calculated according to Equation (1):

$$SOC_{nm} = \sqrt{\sum_i \left| \langle \psi_{S_n} | \hat{H}_{SO} | \psi_{T_{i,m}} \rangle \right|^2}; \qquad i = x, y, z$$

Where $\hat{H}_{SO}$ is the spin-orbit Hamiltonian with effective nuclear charge. Relativistic corrections were obtained by the zeroth order regular approximation (ZORA). Thus, ZORA-DEF2-SVP and SARC-ZORA-SVP for the main and metal atoms, respectively were used. The RIJCOSX approximations was introduced to speed up the calculations' time, as suggested in the ORCA manual. Very tight SCF convergence and a very large grid (Lebedev 770 points) were set for such calculations.

The optimized structures of all the triplet excited states potentially involved into the photodecomposition mechanism of **Pt-Fe** were searched using the TDDFT method in implicit water solvent. The obtained guesses were then used as starting points for the optimization within the unrestricted Kohn–Sham formalism (UKS) with UB3LYP.[23,24]

DFT calculations were carried out using the X-ray crystal structure of **Pt-Fe** as a starting point. The optimised molecular structure is obtained at the B3LYP-D3/SDD/6-31G* level of theory. **Absorption spectra.** To accurately describe the photophysical features of **Pt-Fe**, a preliminary benchmark study was carried out on the absorption spectrum of the complex and compared with that experimentally detected. For the selection of the most appropriate protocol, the focus was on the reproduction of the experimental spectrum, concentrating on both high and low energy regions. The performance of several exchange and correlation functionals was examined. From this data, the B3LYP-D3 functional emerges as the better choice to reproduce the electronic absorption spectrum. **Excited states properties.** With the aim to shed light on the behaviour of **Pt-Fe** under irradiation, a detailed TDDFT analysis of the excited states was carried out. As it is generally accepted that the lifetime of singlet excited states is five times shorter than that of a triplet states (10^{-8} s *vs* 10^{-3} s), it seems reasonable to assume that the photochemical process, which **Pt-Fe** should be exposed to, involves predominantly excited triplet states populated by an efficient intersystem spin crossing (ISC) process. According to the Fermi Golden Rule,[25] the ISC kinetics is directly related

to the spin–orbit coupling matrix elements and the adiabatic energy difference between singlets and triplets. Thus, in this work, both spin-orbit coupling constants (SOCs) and S–T adiabatic energy differences (ΔE) were calculated. However, in order to establish which triplet state is most likely to be involved into the photodecomposition process of **Pt-Fe**, a careful analysis of the TDDFT outcomes was performed.

Triplet state features. In order to properly identify and characterise the plausible triplet states involved in the photoactivity of **Pt-Fe**, the geometries of the states, Tn and Tm, found within the vertical approximation and considered in the SOC calculations, were primarily optimised at the TD-B3LYP level of theory and, then, within the unrestricted Kohn–Sham formalism (UKS).[23,24] The optimised structures obtained by the TDDFT calculations and the spin density distributions are reported. Such geometries were used as initial guess for the final UKS optimisations. Applying this approach, all the examined triplet excited states collapsed in three different triplets, named Ta, Tb and Tc. Their optimised structures together with the relative spin density distribution, were used to establish their character, and the relative energies calculated with respect the ground state S0. The main bond distances of the calculated triplet states are compared with those of the S0 ground state. In particular, the triplet state labelled Ta is characterised by an elongation of the distance between the two planar Cp rings and the Fe centre of the ferrocenyl moiety, making the state essentially centred on the metal, as evidenced by the spin density exclusively localised on Fe (1.96). No notable geometric variation occurs around the platinum centre. The second triplet state Tb appears to be characterised by a significant reorganisation in the coordination sphere of the Pt centre. The bond with both equatorial N_3 ligands is weakened and the Pt-N bond distances increase by up to approximately 0.4 Å. This state is centred on both platinum and iron metals with a localised spin density of 0.48 and 1.22, respectively. Finally, a considerable elongation of the Pt–O bonds with both the oxygen atoms of the ligands in axial positions, features the highest triplet state, Tc, obtained by the distribution of the spin density on both metals of Pt-Fe complex, with a behaviour similar to that of the Tb state.

Photoreaction with 5’-GMP. 2 mol. equiv. of guanosine 5′-monophosphate disodium salt hydrate (5′-GMP-Na_2) was mixed with 30 μM complex in aqueous solution. The solution was irradiated for 1 h (420 or 520 nm) and analysed immediately on a Bruker Amazon X mass spectrometer connected online with HPLC.

Cell culture. A number of human cell lines, ovarian carcinoma A2780, lung adenocarcinoma A549, prostate cancer PC3 cells and lung fibroblasts MRC-5 cell were obtained from the European Collection of Animal Cell Culture (ECACC), Salisbury, UK. Bladder cancer T24 and SW-780 cells were provided by Dr Richard T. Bryan from University of Birmingham. A2780, A549, PC3 and MRC-5 cell lines used in this work were grown in Roswell Park Memorial Institute media (RPMI-1640), while T24 and SW-780 were grown in Dulbecco's Modified Eagle Medium (DMEM). All media was supplemented with 10% v/v of foetal calf serum (FCS) and 1% v/v penicillin/streptomycin. The adherent monolayers of cells were grown at 310 K in a humidified atmosphere containing 5% CO_2 and passaged regularly at *ca*. 80% confluence.

Photo-dark cytotoxicity. Approximate 1.5×10^4 cells were seeded per well in 96-well plates. Independent duplicate plates were used, one for the dark experiment, the other for the irradiation experiment. The cells were pre-incubated in drug-free medium with phenol red at 310 K for 24 h. Complexes were dissolved first in DMSO and then diluted in phenol red-free medium to make the stock solution of the drug. These stock solutions were further diluted using phenol-red free cell culture medium until working concentrations were achieved, the maximum DMSO concentration was < 0.5% v/v in these solutions. Cells were exposed to the drugs with different concentrations for 1 h. Then one plate was irradiated for 1 h using blue light (4.8 mW cm^{-2} per LED at 465 nm) or green light (11.7 mW cm^{-2} per LED at 520 nm) while the dark plate was kept in the incubator. After irradiation, supernatants of both plates were removed by suction and the cells were washed with PBS. Photocytotoxicity was determined after another 24 h recovery at 310 K in drug-free phenol red-containing medium by comparison to untreated controls which were only exposed to vehicle. Untreated controls were also compared between the irradiated and the non-irradiated plates to ensure that the differences in cell survival were not statistically relevant, hence guaranteeing that the differences in cell viability observed were not due to the light source. The SRB assay was used to determine cell viability.[26] Absorbance measurements of the solubilised dye (on a Promega microplate reader) allowed the determination of viable treated cells compared to untreated controls. IC_{50} values (concentrations which caused 50% of cell death) were determined as the average of triplicates and their standard deviations were

calculated. Stock concentrations for all metal complexes used in these biological assays were adjusted/verified after ICP-OES metal quantification.

Time-dependence of photocytotoxicity. These experiments were carried out as described under **Photo-dark cytotoxicity** with variable irradiation times: 30 min and 1 h.

Photo-dark cytotoxicity under normoxia and hypoxia. About 1×10^4 cells were seeded per well in 96-well plates. Independent duplicate plates were used, one for dark while the other for irradiation experiment. The cells were pre-incubated in drug-free medium with phenol red at 310 K for 24 h. For normoxia experiments, drugs were added after pre-incubation, while for hypoxia experiments, plates were transferred to hypoxia chamber and allowed another 24 h incubation under hypoxia environment, and all the rest experiments were carried out under hypoxia. Complexes were dissolved first in DMSO and then diluted in phenol red-free RPMI-1640 until working concentrations were achieved, the DMSO concentration was adjusted to 0.05% v/v in these solutions. Cells were exposed to drugs with different concentrations for 1 h. Then one plate was irradiated for 1 h using blue light (4.8 mW cm^{-2} per LED at 465 nm) or green light (11.7 mW cm^{-2} per LED at 520 nm) while the dark plate was kept in the incubator. After irradiation, cells were incubated for another 72 h at 310 K under corresponding oxygen concentration without drug removal. Photocytotoxicity was determined by the SRB assay as described in the **Photo-dark cytotoxicity** section above.

Platinum accumulation in cancer cells in the dark. For Pt cellular accumulation studies, *ca*. 5×10^6 A2780, A549, PC3 and T24 cells were plated in 100 mm Petri dishes and allowed to attach for 24 h. Then the plates were exposed to complexes at 10 μM or equipotent concentrations equal to the photoactive IC_{50} values (465 nm, 1 h) in the corresponding cell line. Additional plates were incubated with medium alone as a negative control. After 1 h of incubation in the dark at 310 K, the cells were rinsed three times with cold PBS and harvested by trypsinisation. The number of cells in each sample was counted manually using a haemocytometer. Then the cells were centrifuged to obtain the whole cell pellet for ICP-MS analysis. All experiments were conducted in triplicate.

Photo/dark platinum accumulation. These experiments were carried out as described in **Platinum accumulation in cancer cells in the dark** with modification. Cells were

exposed to complexes at equipotent concentrations equal to the photoactive IC_{50} values (for irradiation at 465 nm) in the A2780 or T24 cell line, incubated in the dark for 1 h then irradiated with blue light (465 nm) for 1 h. Dark controls were obtained after 2 h incubation for comparison.

Intracellular distribution. *ca*. 5×10^6 or 1×10^7 A549 cells were plated in 100 mm Petri dishes and allowed to attach for 24 h. Then the plates were exposed to complexes at 10 μM in the dark for 1 h at 310 K. Cells were rinsed three times with cold PBS and harvested by trypsinisation. The number of cells in each sample was counted manually using a haemocytometer. Nucleus/CytosolFractionation Kit and Mitochondria/Cytosol Fractionation Kit were used extract nuclei and mitochondria, respectively.

ICP-MS sample preparation. Whole cell pellets in Eppendorf tubes were dissolved in concentrated 72% v/v nitric acid (200 μL), and heated in an oven at 343 K overnight. The samples were then allowed to cool, and each cellular sample solution was transferred into a Falcon tube and diluted with Milli-Q water (3.8 mL), to obtain a final HNO_3 concentration of *ca*. 3.6% v/v.

Singlet oxygen ($^1O_2$) detection. Singlet oxygen generation was detected using the fluorescent probe Singlet Oxygen Sensor Green by SOSG. An aqueous solution of **Pt-Fe** (50 μM) and SOSG (1 μM) was irradiated with blue light (463 nm) and its fluorescence spectrum were measured every 10 s with λ_{ex} = 504 nm, λ_{em} = 525 nm.

Detection of H_2O_2. 10 μL of Pt(IV) complexes (5 mM) in aqueous solution was dropped on a peroxide test stick (Quantofix® Peroxide 25). The stick treated with aqueous solution of Pt(IV) complexes was irradiated with blue light (463 nm) for 30 s. The concentration of H_2O_2 was semi-quantitatively measured by comparing to the colour scale.

Electron paramagnetic resonance (EPR) spectroscopy. The EPR spectra were recorded on a Bruker EMX (X-band) spectrometer at 298 K. Samples (*ca*. 100 μL) in aqueous solution were prepared and transferred using a plastic syringe with metal needle to a standard quality quartz tube with inner diameter of 1.0 mm and outer diameter of 2.0 mm (Wilmad LabGlass) and sealed with parafilm. Using the y-incremental sweep mode of 2 with an accumulation of 100 scans in the x dimension. Typical key EPR spectrometer settings were modulation amplitude 1.0 G, microwave

power 6.32 mW, receiver gain 1.0 $\times 10^5$, conversion time 5.12 ms, time constant 5.12 ms, sweep width 200 G. The blue LED (465 nm) was mounted within the EPR magnet, supported by a foam sponge, to maintain its position throughout the EPR measurements. The distance from the tip of the irradiation light bulb to the EPR cavity was *ca*. 3 cm. Data were processed by Matlab R2016b with easyspin 5.1.12.

Intracellular ROS measurement. 2',7'-Dichlorofluorescin diacetate (DCFH-DA, Sigma-Aldrich) was used to determine the intracellular ROS production in the dark and after irradiation when A549 cells were exposed to Pt(IV) complexes. DCFH-DA is cell permeable non-fluorescent probe that turn to the highly fluorescent dye DCF upon oxidation by ROS in cells. A549 cells were seeded in 96-well plates (*ca*. 10^4 cells per well, 24 h for attachment) then exposed to complexes at various concentration for 1 h. One plate was irradiated for 1 h with blue light (465 nm), while the other one was kept in the dark. Drugs were removed and DCFH-DA (20 μM) was added to cells and incubated for 40 min. Cells were washed by Hank's Balanced Salt Solution (HBSS). The fluorescence intensity was measured on a Promega microplate reader with comparison to negative control.

Confocal fluorescence microscopy. The fluorescence images were recorded on a fluorescence confocal microscope (LSM 880, AxioObserver). A549 cells (*ca*. 1×10^5) were seeded in glass bottom cell culture dishes (CELLview) and cultured 24 h for attachment, then exposed to complexes in the absence and presence of NAC for 1 h in dark then 1 h irradiation with blue light (465 nm). Drugs were removed and cells were incubated with DCFH-DA (20 μM) for 40 min. Cells were washed by HBSS before measurement.

Intracellular GSH measurement. A549 cells (*ca*. 5×10^6) were seeded in 100 mm Petri dishes and cultured 24 h for attachment. Cells were exposed to complex at $1\times IC_{50}$ concentration (465 nm) for 1 h in dark then 1 h irradiation with blue light (465 nm). Drugs were removed and cells were treated using Glutathione Colorimetric Detection Kit (Invitrogen™).

Intracellular GPX4 expression. A549 cells (*ca*. 5×10^6) were seeded in 100 mm Petri dishes and cultured 24 h for attachment. Cells were exposed to complex at $1\times IC_{50}$ concentration (465 nm) for 1 h in dark then 1 h irradiation with blue light (465 nm), allowed recovery in fresh medium for 24 h. Cells were harvested and washed once in

PBS by centrifugation at 100 RMP and kept on ice. The cells were resuspended in RIPA buffer and homogenized by 20 strokes of a syringe. The cells were incubated on ice for 10 min. The cells were then centrifuged at 14000 RPM for 10min. Bradford assay was performed using BSA as the standard. 20 µg of protein was loaded on a 4-20% Gradient Gel (BioRad) and run at 120V in a Mini Protean Tetra System. The gel was transferred on nitrocellulose membrane (Amersham 10600001) using a Trans Blot Semi Dry apparatus (BioRad). The blots were blocked in 5% milk for 1h at RT. The blots were exposed to anti-GPX4 antibody (ab125066), 1:1000, treated overnight at 277 K and anti-beta Actin antibody (ab8227), 1:5000, exposed for 1h at 298 K. The blots were washed 3× in TBST. Rabbit polyclonal HRP secondary antibody (ab205718) was used after the primary antibody and incubated at 1:10000 dilution for 1 h at 298 K. The blots were washed 3× with TBST and then exposed to the Thermo West Pico Substrate (34580). The blots were imaged in a Chemi Doc (BioRad).

Mechanism of action. Experiments were carried out using a BD LSR II flow cytometer.

Lipid peroxidation assay. A549 cells (*ca*. 5×10^5) were seeded in 6-well plates and cultured 24 h for attachment. Cells were exposed to complex at 0.5× or $1\times IC_{50}$ concentration (465 nm) for 1 h in dark then 1 h irradiation with blue light (465 nm). Drugs were removed and cells were incubated with BODIPY™ 581/591 C11 (5 μM) for 30 min. Cells were washed by HBSS before measurement.

Apoptosis assay. A549 cells (*ca*. 5×10^5) were seeded in 6-well plates and cultured 24 h for attachment. Cells were exposed to complex at 0.5× or $1\times IC_{50}$ concentration for 1 h in dark then 1 h irradiation with blue light (465 nm), then washed with PBS and treated with Annexin V-FITC/PI kit (Abcam). A2780 and A2780cis cells (*ca*. 5×10^5) were seeded in 6-well plates and cultured 24 h for attachment. Half of the plates were moved to the hypoxia chambers and left for 24 h and all subsequent experimental steps were carried out under hypoxia and normoxia. Drug treatments under these two conditions were exactly the same except for the oxygen concentration. Cells were exposed to complex for 1 h in dark then 1 h irradiation with blue light (465 nm), and further incubated with drugs for 72 h after irradiation, then washed with PBS and treated with Annexin V-FITC/PI kit (Abcam).

Table S1. Uv-vis absorption spectra of **Pt-Fe**, **1**, ferrocene (Fc) and ferrocene carboxylic acid (Fc-COOH) in various solvents at 298 K.

Compound	λ_{abs}/nm (ε_{max}/M^{-1}cm^{-1})		
	H_2O[a]	PBS[a]	DMSO
**Pt-Fe**	260 (22364); 299 (21714); 435 (528)	260 (27522); 298 (26527); 434 (626)	265 (28744); 303 (29607); 432 (622)
**1**	259 (10721); 293 (17412)	259 (10890); 293 (17704)	264 (11128); 297 (16130)
Fc[b]	-	-	442 (118)
Fc-COOH	260 (5873); 305 (1332); 440 (289)	258 (5359); 301 (1215); 441 (228)	305 (1165); 348 (441); 442 (241)

[a] 0.5% DMSO is added to facilitate dissolution; [b] data on H_2O and PBS are not obtained due to poor solubility.

Table S2. Crystallographic data and structure refinement for **Pt-Fe**.

Complex	**Pt-Fe**
CCDC code	2234482
Empirical formula	$C_{23}H_{23}FeN_9O_4Pt$
Formula weight	740.44
Temperature/K	150(2)
Crystal system	monoclinic
Space group	$P2_1/c$
a/Å	15.1269(3)
b/Å	10.50172(17)
c/Å	16.1559(3)
α/°	90
β/°	101.4247(19)
γ/°	90
Volume/Å^3	2515.64(8)
Z	4
ρ_{calc}g/cm^3	1.955
μ/mm^{-1}	6.181
F(000)	1440.0
Crystal size/mm^3	0.32 × 0.3 × 0.22 orange block
Radiation	MoKα (λ = 0.71073)
2Θ range for data collection/°	4.754 to 60.628
Index ranges	$-19 \le h \le 19$, $-14 \le k \le 14$, $-22 \le l \le 21$
Reflections collected	24793
Independent reflections	6758 [R_{int} = 0.0362, R_{sigma} = 0.0399]
Data/restraints/parameters	6758/0/344
Goodness-of-fit on F^2	1.047
Final R indexes [I>=2σ (I)]	R_1 = 0.0251, wR_2 = 0.0461
Final R indexes [all data]	R_1 = 0.0320, wR_2 = 0.0487
Largest diff. peak/hole/e Å^{-3}	0.71/-1.06

Table S3. Selected bond lengths (Å) and bond angles (°) for **Pt-Fe**.

Pt1–O1	1.9660(19)	O1–Pt1–O19	170.63(7)
Pt1–O19	2.0622(18)	N13–Pt1–N7	177.32(8)
Pt1–N13	2.047(2)	N4–Pt1–N1	178.53(10)
Pt1–N1	2.059(2)	C24–Fe1–C30	107.69(12)
Pt1–N7	2.051(2)	C24–Fe1–C28	40.91(12)
Pt1–N4	2.042(2)	C24–Fe1–C33	156.53(15)
Fe1–C30	2.042(3)	C24–Fe1–C26	68.83(12)
Fe1–C24	2.037(3)	C24–Fe1–C29	120.93(13)
Fe1–C28	2.040(3)	C24–Fe1–C31	124.90(13)
Fe1–C33	2.041(3)	C24–Fe1–C32	161.51(14)
Fe1–C26	2.046(3)	C24–Fe1–C27	68.27(12)
Fe1–C29	2.039(3)	C25–Fe1–C24	41.55(11)
Fe1–C25	2.036(3)	C26–C25–C24	107.2(3)
Fe1–C31	2.043(3)	O19–C20–C21	115.3(2)
Fe1–C32	2.045(3)	O20–C20–O19	127.2(3)
Fe1–C27	2.059(3)	N22–C23–C24	117.1(2)
N1–N2	1.215(3)	C23–N22–C21	121.1(2)
N2–N3	1.148(3)		
N4–N5	1.203(3)		
N5–N6	1.141(4)		

Table S4. Selected hydrogen bond parameters for **Pt-Fe**.

**D**	**H**	**A**	**d(D-H)/Å**	**d(H-A)/Å**	**d(D-A)/Å**	**D-H-A/°**
O1	H1	O23[1]	0.84	1.87	2.704(3)	169.6
N22	H22	O20[2]	0.88	2.05	2.819(3)	145.7

[1]+X, 3/2-Y, -1/2+Z; [2]1-X, -1/2+Y, 3/2-Z

Table S5. Photochemical decomposition products (420 nm irradiation) from complex **Pt-Fe** detected by LC-MS (positive ion mode).

Peak[a]	Formula	Structure	Calcd (m/z)	Found[b] (m/z)
a	$C_{14}H_{16}N_4Pt$	$\{Pt^{II}(CH_3CN)_2(py)_2]^{2+}$	217.5512	217.5507
	$C_{12}H_{14}N_3OPt$	$\{Pt^{II}(CH_3CN)(OH)(py)_2]^{+}$	411.0785	411.0784
	$C_{14}H_{17}N_3O_4Pt$	Unknown	486.0867	486.0926
b	$C_{10}H_{10}N_8PtK$	$[\{Pt^{II}(py)_2(N_3)_2\}+K]^{+}$	476.0313	476.0246
c	$C_{13}H_{14}N_3O_2Pt$	$\{Pt^{II}(py)_2(CH_3CN)(HCOO)\}^{+}$	439.0734	439.0676
d	$C_{23}H_{23}N_3O_5FePt$	Unknown	672.0635	672.1314
e	$C_{18}H_{18}N_9O_4FePt$	$\{Pt^{IV}$(gly-Cp-Fe)$(py)_2(N_3)_2(OH)\}^{+}$	675.0479	675.1004
f	$C_{12}H_{13}N_6Pt$	$\{Pt^{II}(py)_2(N_3)(CH_3CN)\}^{+}$	436.0849	436.0846
g	$C_{23}H_{24}N_3O_4FePt$	$[\{Pt^{II}$(gly-Fc)$(py)_2(OH)\}+H]^{+}$	657.0764	657.1353
h	$C_{25}H_{25}N_4O_3FePt$	$\{Pt^{II}$(gly-Fc)$(py)_2(CH_3CN)\}^{+}$	680.0924	680.0836
i	$C_{27}H_{30}N_5O_5FePt$	$[\{Pt^{IV}$(gly-Fc)$(py)_2(OH)_2\}+2\ CH_3CN]^{+}$	755.1244	755.1171
j	$C_{13}H_{14}NO_3Fe$	[gly(COOH)-Fc+H]$^{+}$	288.0323	288.0226
k	$C_{23}H_{23}N_6O_4FePt$	$\{Pt^{IV}$(gly-Fc)$(py)_2(N_3)(OH)\}^{+}$	698.0778	698.0674
l	$C_{23}H_{22}N_9O_3FePt$	$\{Pt^{IV}$(gly-Fc)$(py)_2(N_3)_2\}^{+}$	723.0843	723.1282

[a] See **Figure S14**; [b] HRMS for samples separated by HPLC.

Table S6. Photochemical decomposition products (420 nm irradiation) from complex **1** detected by LC-MS (positive ion mode).

Peak[a]	Formula	Structure	Calcd (m/z)	Found[b] (m/z)
a1	$C_{10}H_{12}N_2O_2Pt$	$\{Pt^{III}(py)_2(OH)_2\}^{+}$	387.0547	387.0540
b1	$C_{11}H_{11}N_5O_2Pt$	$\{Pt^{III}(py)_2(N_3)(HCOO)\}^{+}$	440.0560	440.0687
c1	$C_{12}H_{13}N_6Pt$	$\{Pt^{II}(py)_2(N_3)(CH_3CN)\}^{+}$	436.0849	436.0846

[a] See **Figure S15**; [b] HRMS for samples separated by HPLC.

Table S7. Photochemical decomposition products (517 or 593 nm irradiation) from complex **Pt-Fe** detected by LC-MS (positive ion mode).

**Peak**[a]	**Formula**	**Structure**	**Calcd (m/z)**	**Found**[b] **(m/z)**
α	$C_{18}H_{18}N_9O_4FePt$	$\{Pt^{IV}(gly\text{-}Cp\text{-}Fe)(py)_2(N_3)_2(OH)\}^+$	675.0479	675.1004
β	$C_{12}H_{13}N_6Pt$	$\{Pt^{II}(py)_2(N_3)(CH_3CN)\}^+$	436.0849	436.0846
γ	$C_{20}H_{20}N_6O_3FePt$	$\{Pt^{II}(gly\text{-}Fc)(N_3)(py)(CH_3CN)\}^+$	643.0594	643.1118

[a] See **Figures S16** and **S17**; [b] HR-MS for samples separated by HPLC.

Table S8. TD-DFT benchmark for **Pt-Fe** on the structure optimised at B3LYP-D3/6-31G* level in DMSO implicit solvent.

**Functional**	**State**	**λ**	**ΔE**	***f***[a]	**MO Contribution**[b]	λ_{Exp}[a]
**B3LYP-D3**	S10	415	2.99	0.0021	H→L+1 88%	436
	S23	340	3.68	0.0569	H-7→L 22%,	I_{band}
					H-3→L+1 17%	300
**B3PW91**	S11	413	3.00	0.0043	H-3→L 36%,	II_{band}
					H-6→L 29%,	
					H-5→L 18%	
	S24	338	3.67	0.0787	H-7→L 32%,	
					H-2→L+1 15%	
**camB3LYP**	S3	469	2.64	0.0001	H-4→L+7 21%,	
					H-4→L+11 15%	
	S11	319	3.88	0.0854	H-12→L+1 19%	
**TPSS**	S20	450	2.76	0.0117	H-3→L+1 94%	
	S53	331	3.74	0.1619	H-12→L 33%	
**PBE**	S37	402	3.08	0.0130	H-12→L 37%,	
					H-8→L 25%	
	S57	342	3.63	0.0783	H-11→L+1 41%,	
					H-12→L 18%	
**PBE0**	S8	400	3.10	0.0036	H-5→L 62%	
	S20	330	3.76	0.1284	H-7→L 45%	
**M05**	S12	407	3.04	0.0044	H-3→L 54%,	
					H-10→L 18%	
	S21	346	3.58	0.1124	H-7→L 44%	
**M06**	S11	418	2.97	0.0028	H-8→L 41%,	
					H-6→L 32%	
	S21	348	3.56	0.1152	H-7→L 31%	
**M06L**	S20	439	2.83	0.0132	H-3→L+1 99%	
	S34	377	3.28	0.0197	H-12→L 45%,	
					H-11→L 25%	
**M062X**	S5	475	2.61	0.0001	H→L 72%,	
					H-1→L 17%	

	S10	328	3.78	0.0955	H-4→L15%
**M11**	S5	375	2.61	0.0001	H-2→L 81%
	S11	315	3.93	0.1191	H-14→L 24%, H-16→L 19%
**MN12L**	S10	401	3.09	0.0047	H-2→L 77%, H-3→L 20%
	S30	321	3.87	0.0298	H-10→L 48%, H-7→L 37%

[a] The most intense vertical transition in the 400-500 nm range with oscillator strength greater than 0.0001, together with the bright state, are reported. [b] Only contributions larger than 15% are reported.

Table S9. Excitation energies (ΔE, eV), absorption wavelength (λ, nm), oscillator strength (f), MO contribution (%) for selected transitions (Tr) for **Pt-Fe**.

**Tr[a]**	**Band**	**ΔE**	**λ**	***f***	**MO Contribution[b]**	**Theoretical Assignment**
1	I	2.50	495	0.0001	H-3→L 96%	**MC**
2		2.68	463	0.0002	H-2→L+6 35%, H→L+8 23%	**ILCT**
3		2.75	451	0.0001	H-2→L+8 50%, H-1→L+8 32%	**ILCT**
4		2.86	433	0.0002	H→L+1 91%	**MMCT**
5		2.88	430	0.0001	H-1→L+1 92%	**MMCT**
6		2.98	416	0.0010	H-2→L 71%	**MMCT**
7		3.02	410	0.0022	H-5→L 31%, H-2→L 27%, H-6→L 25%	**MMCT**
8	II	3.75	331	0.1280	H-8→L 48%	**LMCT**
9		3.86	321	0.0128	H-4→L+1 56%, H-6→L+1 25%	**LMCT**
10		4.18	297	0.0143	H-11→L 69%	**MMCT**
11		4.20	295	0.0250	H-11→L 23%	**MMCT**
12		4.24	293	0.0246	H-3→L+3 42%,	**LLCT**

					H-14→L 27%	
13		4.27	290	0.0116	H-14→L 34%,<br>H-13→L 16%,<br>H-12→L 16%	**MMCT**
14		4.47	278	0.0279	H-1→L+9 25%,<br>H-1→L+6 19%,<br>H-1→L+10 17%	**ILCT**
15		4.60	270	0.0641	H-17→L 57%	**LLCT**
16		4.83	257	0.0178	H-18→L 55%	**LLCT**
17	III	5.09	243	0.0184	H-3→L+5 30%,<br>H-10→L+3 21%	**LLCT**
18		5.27	235	0.0166	H-4→L+4 60%	**LLCT**
19		5.28	235	0.0443	H-4→L+6 19%,<br>H-12→L+2 18%,<br>H-18→L+1 17%	**LLCT**
20		5.31	234	0.0244	H-18→L+1 25%	**LLCT**
21		5.36	231	0.0174	H-9→L+6 38%,<br>H-4→L+6 26%	**ILCT**
22		5.45	228	0.0145	H-7→L+4 24%,<br>H-14→L+2 24%	**LLCT**

Tr = transition number. [a] Only vertical transitions with oscillator strength greater than 0.01 are reported, with the exception of the vertical transition in the 400-500 nm range with oscillator strength greater than 0.0001. [b] Only contributions larger than 15% are reported.

Table S10. Excitation energies (ΔE, eV), absorption wavelength (λ, nm) and MO contribution (%) for **Pt-Fe** selected triplet states.

**State**	**ΔE**	**λ**	**MO Contribution**[a]	**Theoretical Assignment**
T11	2.71	457	H-6→L 31%,<br>H-8→L 29%,<br>H-5→L 29%	**MC**
T12	2.85	434	H→L+1 86%	**MMCT**

T13	2.88	431	H-1→L+1 90%	**MMCT**
T14	2.95	420	H-3→L+1 29%, H-10→L+1 17%, H-10→L 16%	**MC**
T15	2.99	414	H-2→L 97%	**MMCT**
T21	3.51	352	H→L+3 83%, H→L+2 15%	**MLCT**
T22	3.54	350	H-1→L+3 83%, H-1→L+2 14%	**MLCT**
T23	3.60	344	H-6→L 37%, H-5→L 23%	**LMCT**
T24	3.61	343	H-7→L+1 17%, H-5→L 15%, H-6→L 15%	**LMCT**
T25	3.64	341	H-2→L+1 63%, H-7→L+1 12%	**MMCT**
T26	3.67	338	H-7→L 39%, H-8→L 26%	**LMCT**
T27	3.74	332	H-6→L+1 16%	**LMCT**

[a] Only contributions larger than 10% are reported.

Table S11. IC_{50} values and photocytotoxicity indices (PI) for complex **Pt-Fe** in A2780 and cisplatin-resistant A2780cis ovarian cancer cells after 1 h incubation, 1 h irradiation (blue 465 nm, green 520 nm) and 72 h incubation after treatment. CDDP (cisplatin) and complex **1** were studied for comparison.

Cell		IC_{50} (μM)[a]					
		Normoxia			Hypoxia		
		**Pt-Fe**	**1**	**CDDP**	**Pt-Fe**	**1**	**CDDP**
A2780	Dark	6.0±2.3	11.6±1.8	0.7±0.1	>100	>100	7.6±0.5
	465 nm	0.8±0.2	1.1±0.1	0.9±0.5	1.3±0.3	1.0±0.2	8.8±1.

								7
	520 nm		4.0±0.9	9.9±1.6	0.82±0.02	4.3±0.2	18.0±6.3	9.6±2.3
	P	Blue	7.5	10.5	-	>76.9	>100	-
	I	Green	1.5	1.2	-	>23.2	>5.5	-
A2780cis	Dark		8.7±0.7	21.7±0.1	9.7±1.4	29.8±14.3	>100	14.9±3.2
	465 nm		1.4±0.5	2.8±0.7	7.67±0.04	1.0±0.1	1.4±0.3	16.2±1.0
	520 nm		6.0±1.0	19.3±1.8	10.2±1.2	3.8±1.0	12.2±1.3	16.5±2.9
	P	Blue	6.2	7.8	-	29.8	>71.4	-
	I	Green	1.5	1.1	-	7.8	>8.1	-

[a] Each value is mean of two or three independent experiments.

Table S12. Accumulation of Pt (ng/10^6 cells) in cancer cells after exposure to complexes **1** and **Pt-Fe** (equipotent IC_{50} concentrations, 1 h, in the dark).

Complex	Platinum accumulation (ng/10^6 cells)		
	A2780	A549	PC3
**Pt-Fe**	1.1±0.1**	11.7±3.0*	6.0±0.8**
**1**	0.8±0.2*	10.8±1.4***	13.2±1.1*

[a] All data were determined from triplicate samples and compared with values obtained for untreated cells using a two-tail t-test with unequal variances. *p < 0.05, **p < 0.01, ***p < 0.005.

Table S13. Cellular accumulation of Pt (ng/10^6 cells) in cancer cell after exposure to complexes **1** and **Pt-Fe** (equipotent IC_{50} concentrations).

Complex		Platinum accumulation (ng/10^6 cells)		
		Dark (1 h)	Irrad[a]	PI
**Pt-Fe**	A2780	2.0±0.4**	3.7±0.3***	2
	T24	7.9±0.6***	15.7±2.8*	2
**1**[27]	A2780	0.9±0.2*	2.7±0.3***	3

[a] 1 h incubation, 1 h irradiation (465 nm). All data were determined from triplicate samples and their statistical significance evaluated by a two-tail t-test with unequal variances. *p < 0.05, **p < 0.01, ***p < 0.005.

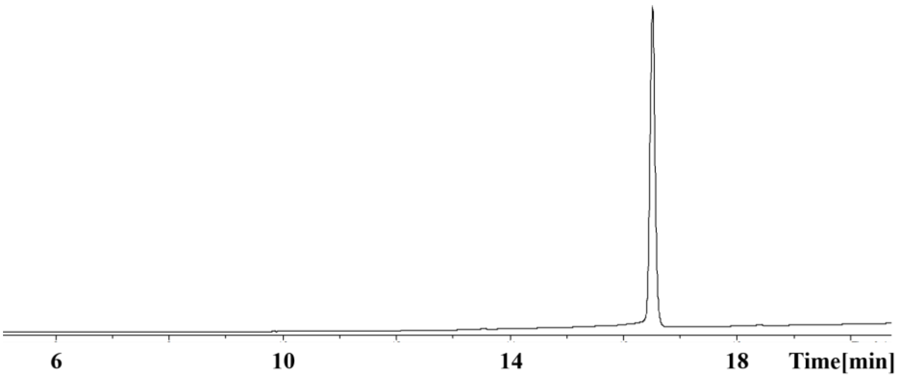

Figure S1. HPLC UV chromatogram for complex **Pt-Fe** confirming its purity (detection wavelength: 254 nm).

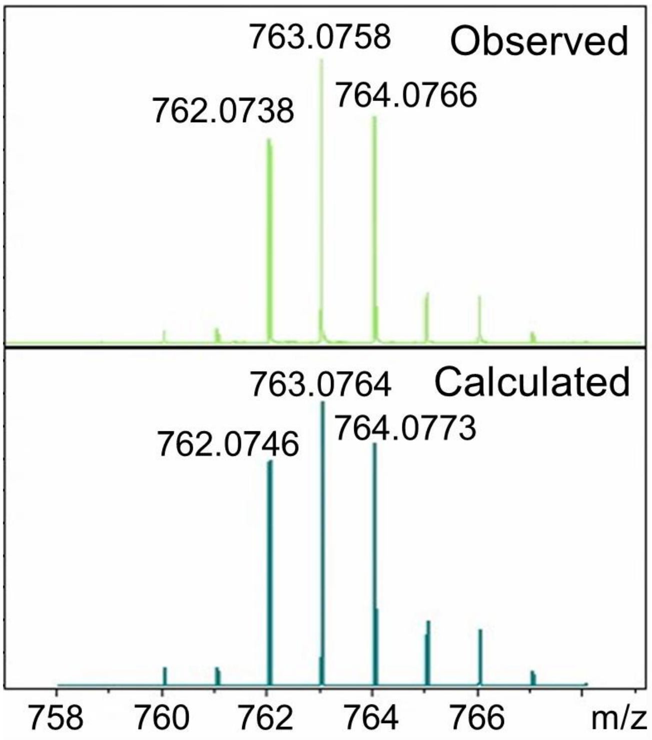

Figure S2. HR-MS of complex **Pt-Fe** at 298 K.

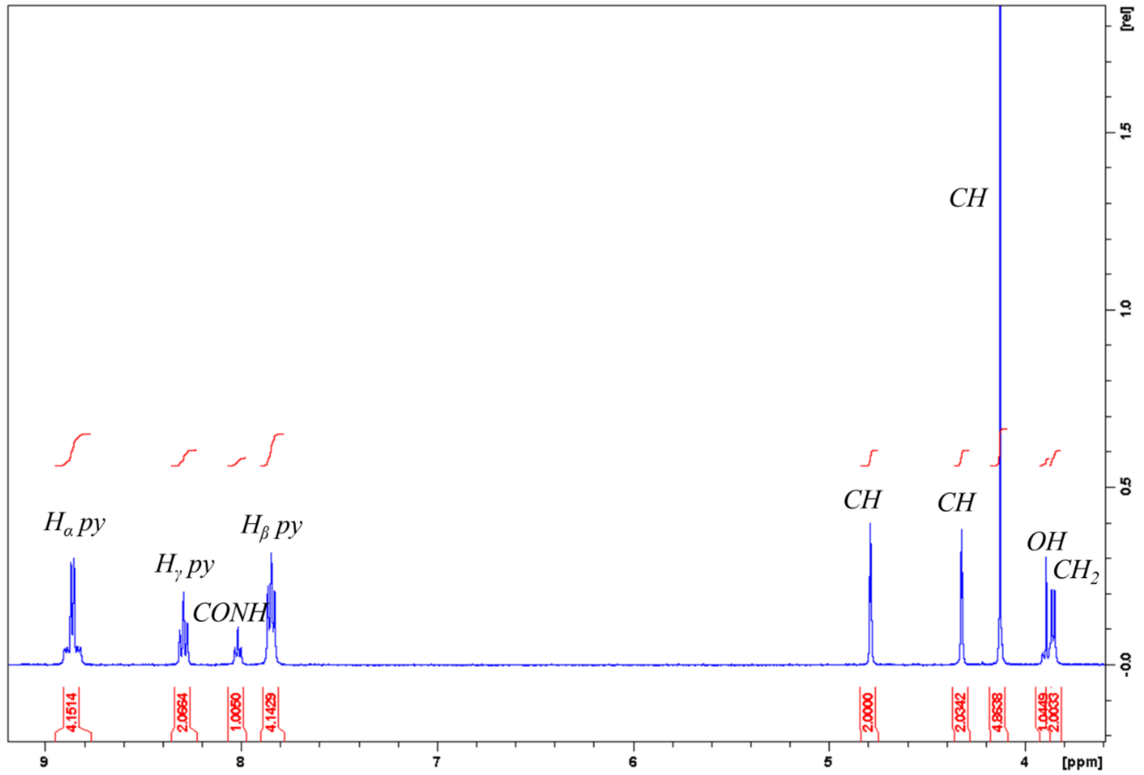

Figure S3. 400 Hz ^{1}H NMR of complex **Pt-Fe** in DMSO-d_6 at 298 K.

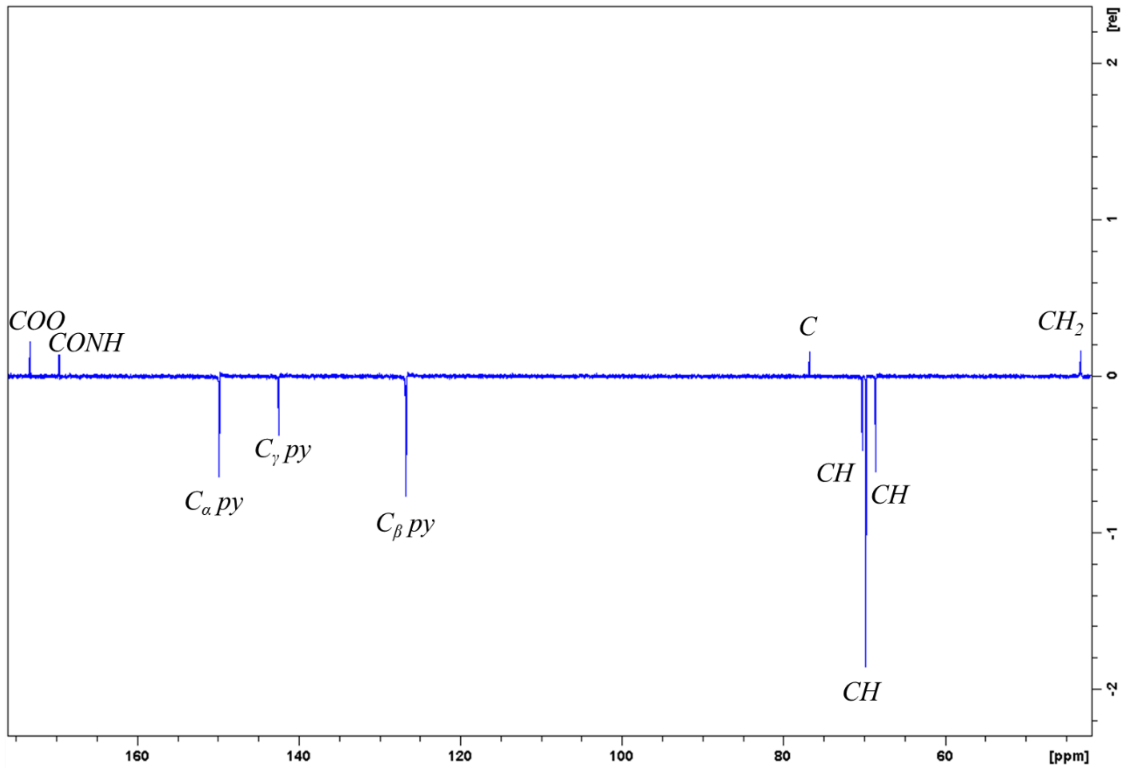

Figure S4. 125 Hz ^{13}C NMR ATP spectrum of complex **Pt-Fe** in DMSO-d_6 at 298 K.

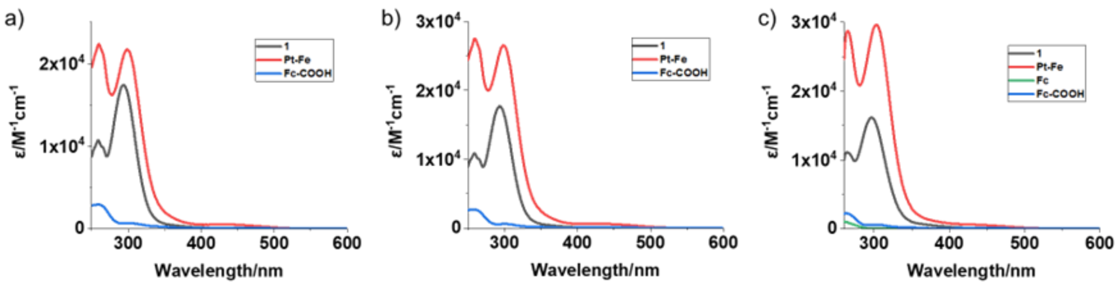

Figure S5. UV-vis spectra of complexes **1**, **Pt-Fe**, Fc, and Fc-COOH in a) H_2O, b) PBS, c) DMSO at 298 K.

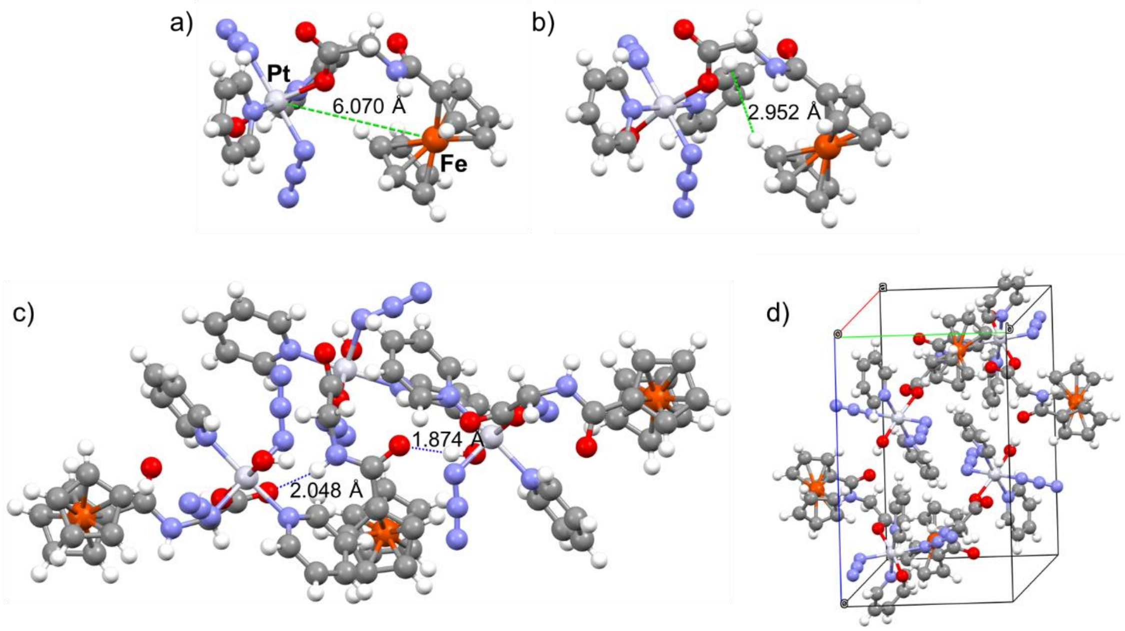

Figure S6. a) The intramolecular distance between Pt and Fe, b) the shortest distance between pyridine and ferrocene protons, c) hydrogen bonds, and d) crystal packing for **Pt-Fe**.

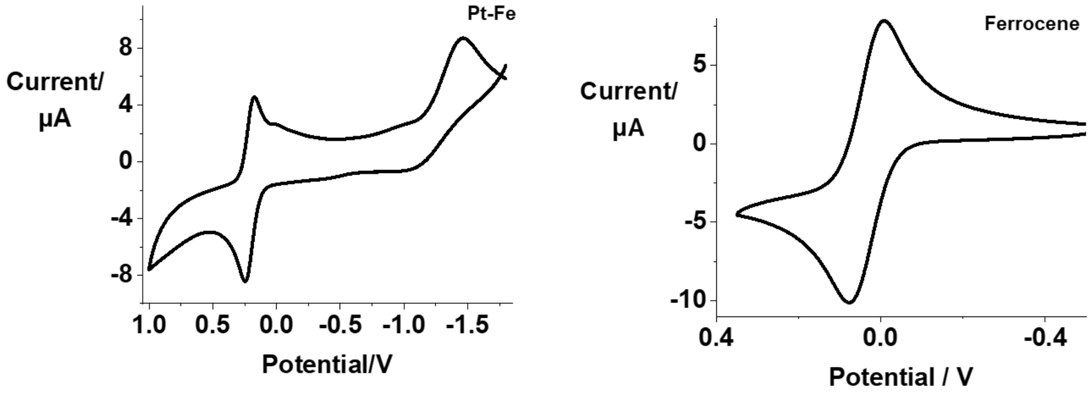

Figure S7. Cyclic voltammograms for complexes **Pt-Fe** and ferrocene (1 mM) in 0.1 M NBu_4PF_6-DMF (under N_2).

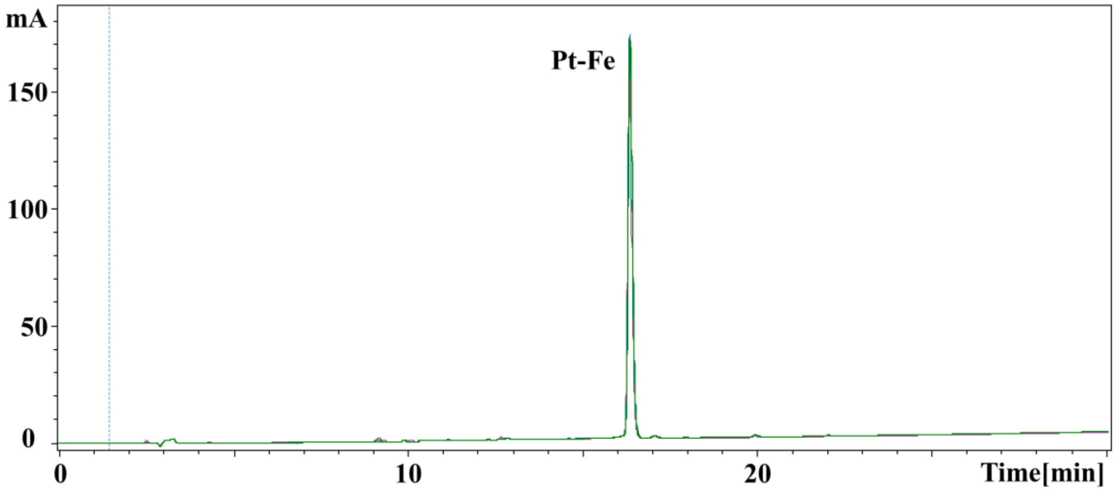

Figure S8. HPLC UV chromatogram for **Pt-Fe** (50 μM) in aqueous solution after incubation in the dark at 310 K for 0, 2 and 24 h.

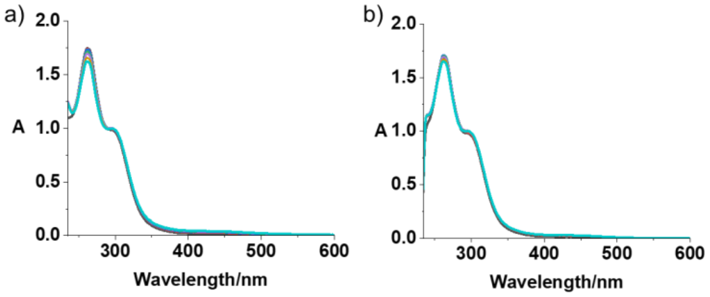

Figure S9. UV-vis spectral changes for **Pt-Fe** (50 μM) in a) air b) N_2-saturated PBS with 5% DMSO in the presence of 50 μM ascorbic acid in the dark at 310 K for 120 min.

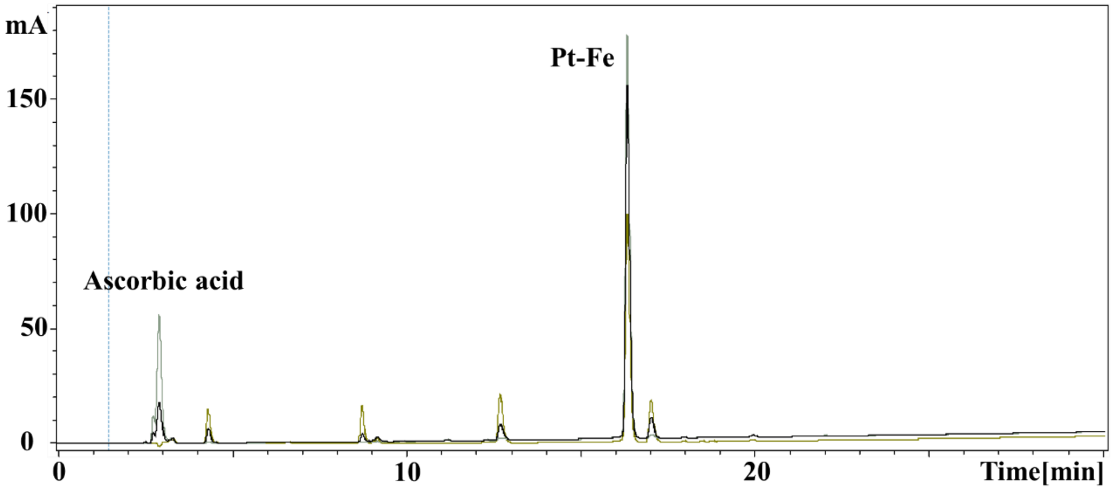

Figure S10. HPLC UV chromatogram for **Pt-Fe** (50 μM) in aqueous solution after incubation in the presence of 50 μM ascorbic acid in the dark at 310 K for 0, 2 and 24 h.

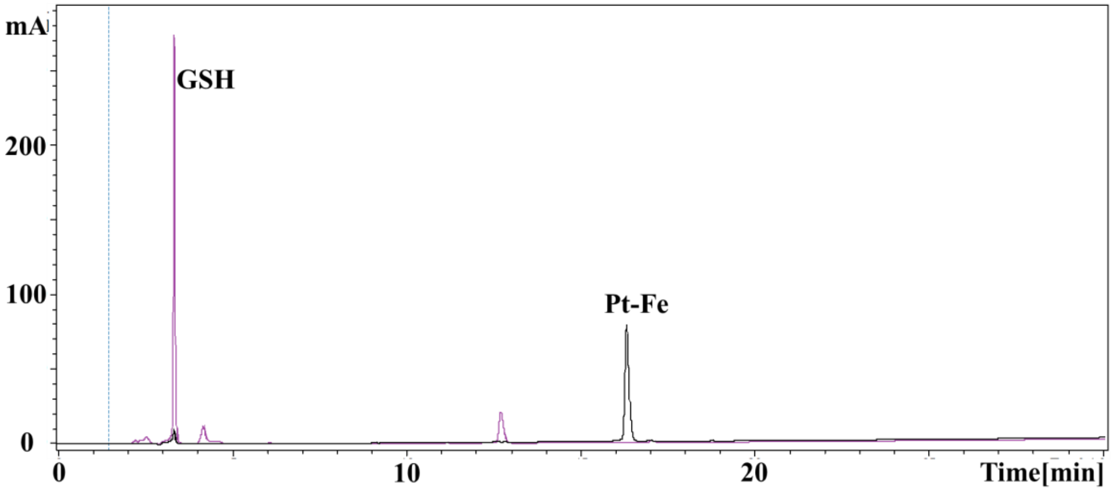

Figure S11. HPLC UV chromatogram for **Pt-Fe** (50 μM) in aqueous solution after incubation in the presence of 2 mM GSH in the dark at 310 K for 0 and 24 h.

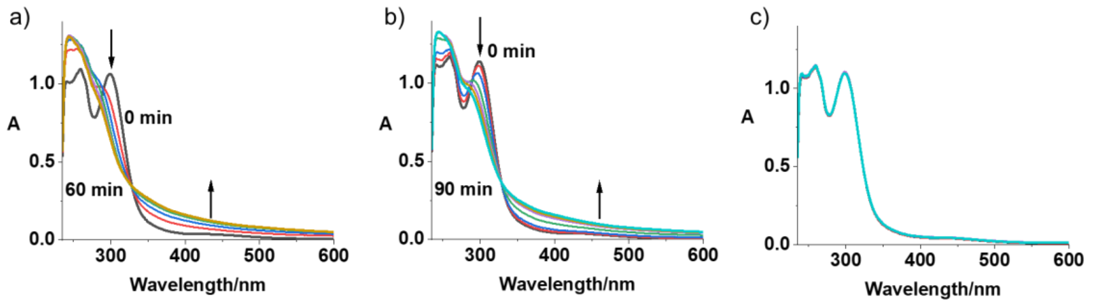

Figure S12. UV-vis spectral changes for **Pt-Fe** (50 μM, N_2-saturated phenol red-free RPMI-1640 with 5% DMSO) upon blue (a, 463 nm, 60 min), green (b, 517 nm, 90 min) or orange (c, 593 nm, 90 min) light irradiation.

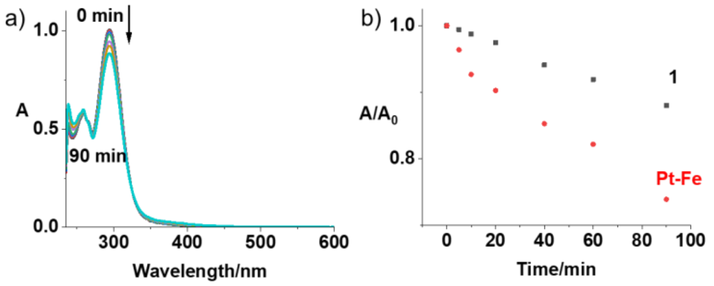

Figure S13. a) UV-vis spectral changes for **1** in phenol red-free cell culture medium RPMI-1640 with 5% DMSO with green light (517 nm); and b) time-dependent absorbance changes of **Pt-Fe** and **1** at 300 nm upon irradiation with green light (517 nm).

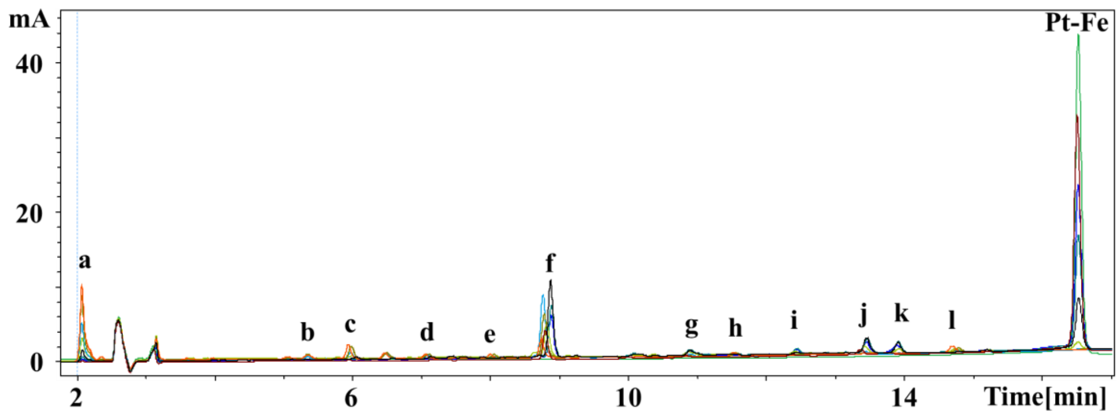

Figure S14. Photochemical decomposition of **Pt-Fe** determined by HPLC after 0 (green), 1, 2, 3, 5, 10, 15, 30 and 60 (orange) min irradiation with indigo light (420 nm), possible species **a**–**l** are listed in **Table S5**.

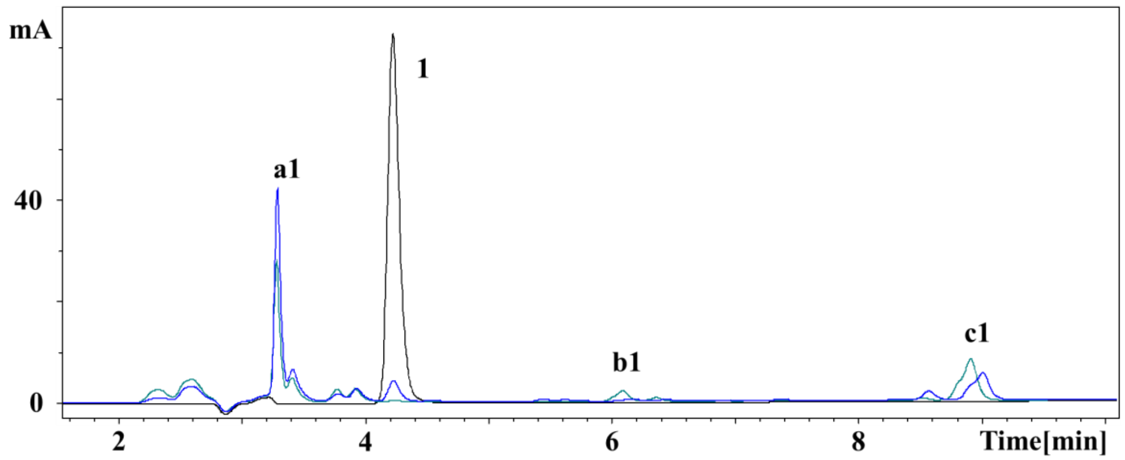

Figure S15. Photochemical decomposition of **1** determined by HPLC after 0 (black), 30 (blue) and 60 (green) min irradiation with indigo light (420 nm), possible species **a1**–**c1** are listed in **Table S6**.

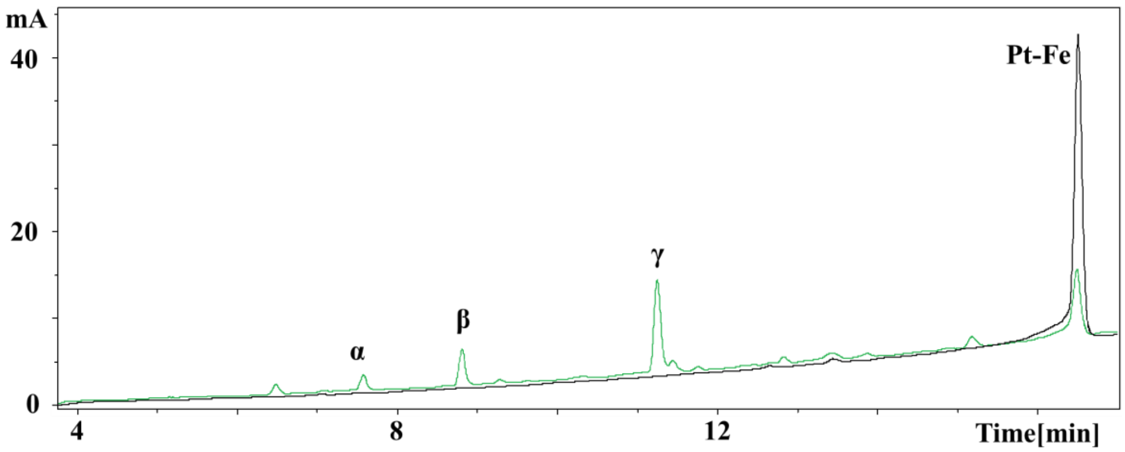

Figure S16. Photochemical decomposition of **Pt-Fe** determined by HPLC after 0 and 60 min irradiation with green light (517 nm), dark (—), green light (—), possible species **α**–**γ** are listed in **Table S7**.

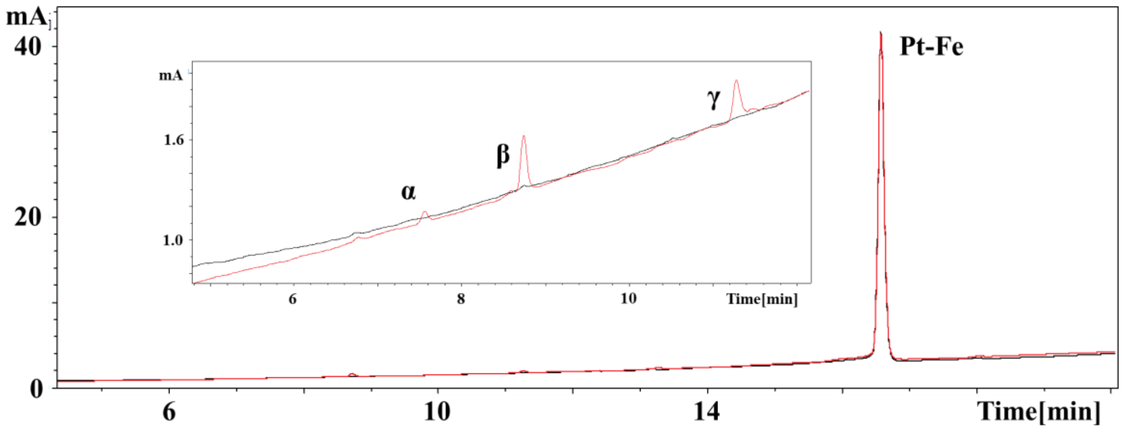

Figure S17. Photochemical decomposition of **Pt-Fe** determined by HPLC after 0 and 60 min irradiation with orange light (593 nm), dark (—), orange light (—), possible species **α**–**γ** are listed in **Table S7**.

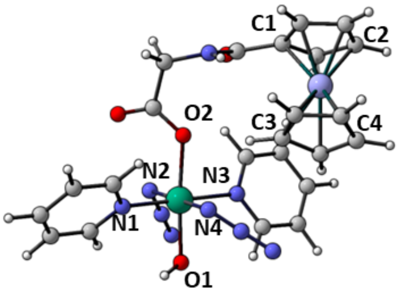

Pt–O1	2.011 (1.966)
Pt–O2	2.079 (2.062)
Pt–N1	2.070 (2.051)
Pt–N2	2.097 (2.042)
Pt–N3	2.087 (2.047)
Pt–N4	2.104 (2.059)
Fe-C1	2.027 (2.037)
Fe-C2	2.053 (2.059)
Fe-C3	2.042 (2.042)
Fe-C4	2.047 (2.045)
O1-Pt-O2	176.7 (170.63)

Figure S18. B3LYP optimised geometrical structure of **Pt-Fe**. Selected bond lengths (in Å) and angle (degrees) are compared with available experimental values (in parentheses).

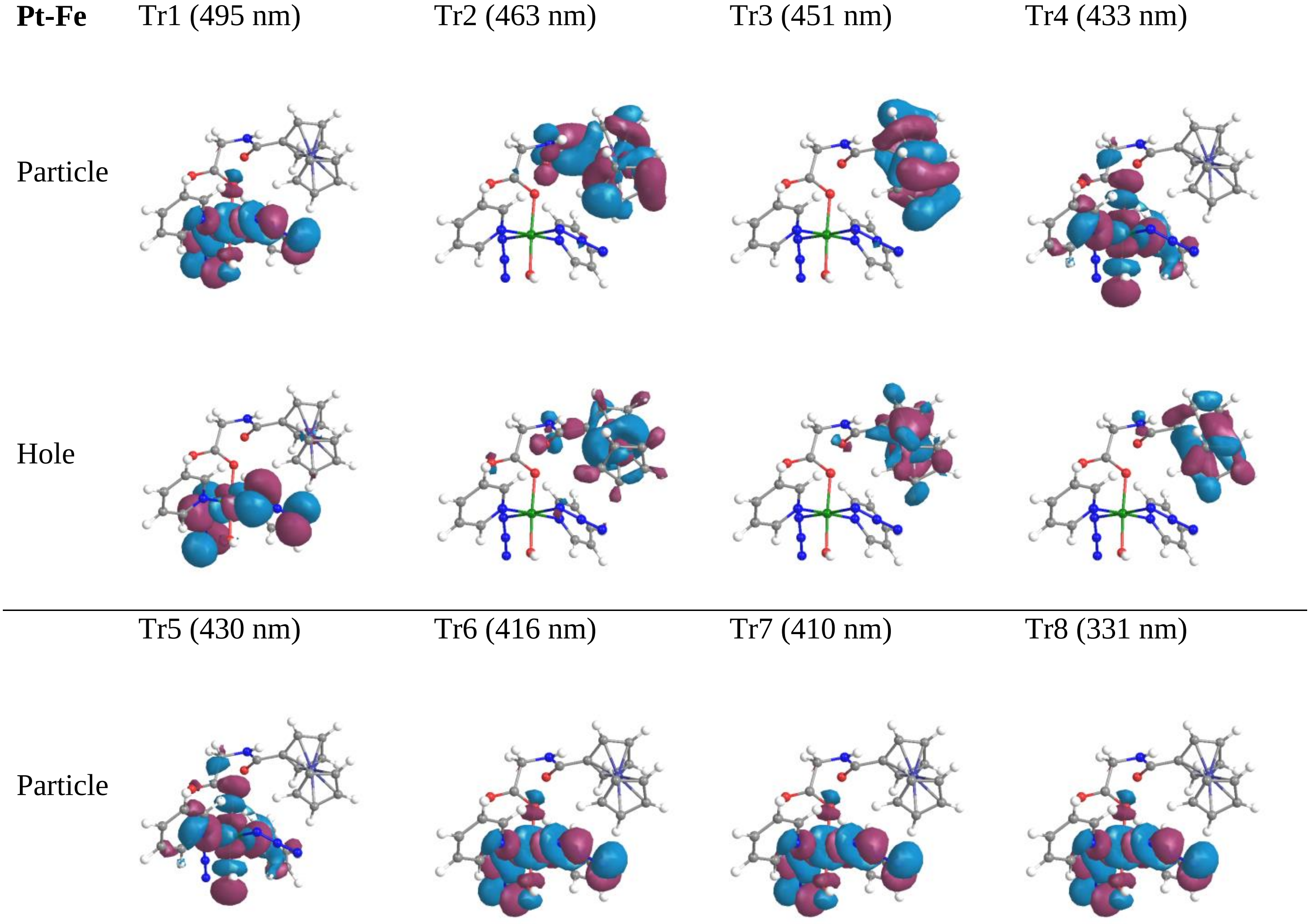

Hole

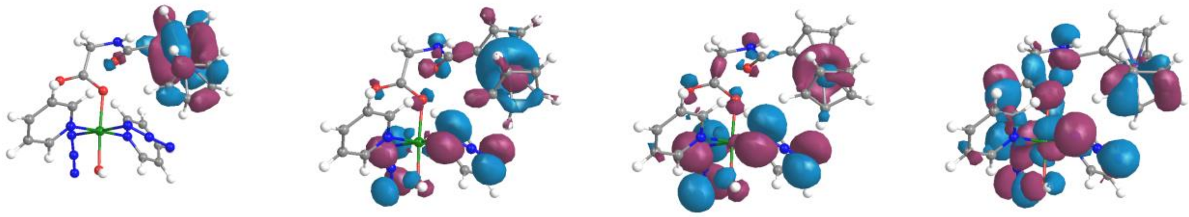

Tr9 (321 nm) Tr10 (297 nm) Tr11 (295 nm) Tr12 (293 nm)

Particle

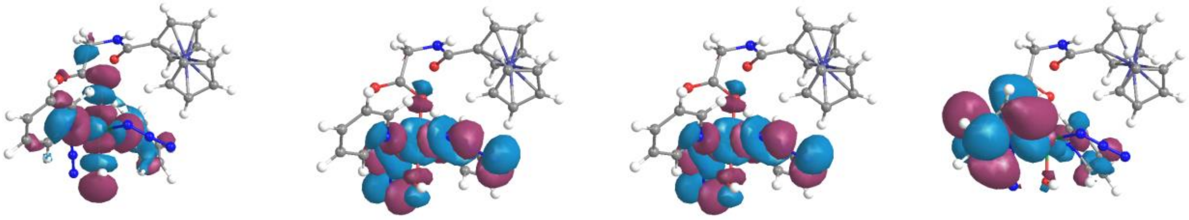

Hole

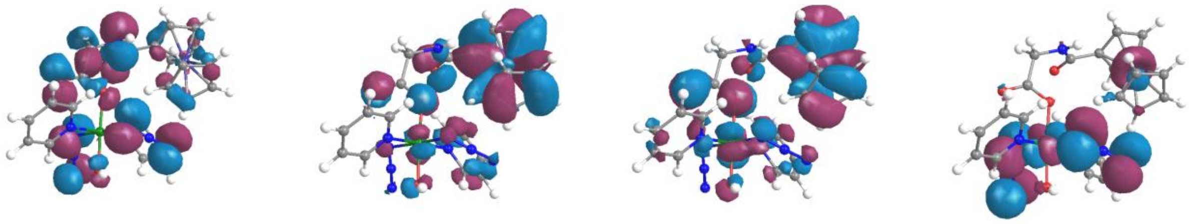

Tr13 (290 nm) Tr14 (278 nm) Tr15 (270 nm) Tr16 (257 nm)

Particle

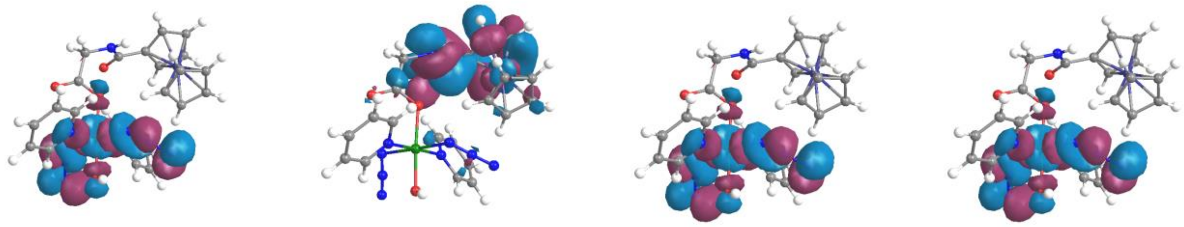

Hole

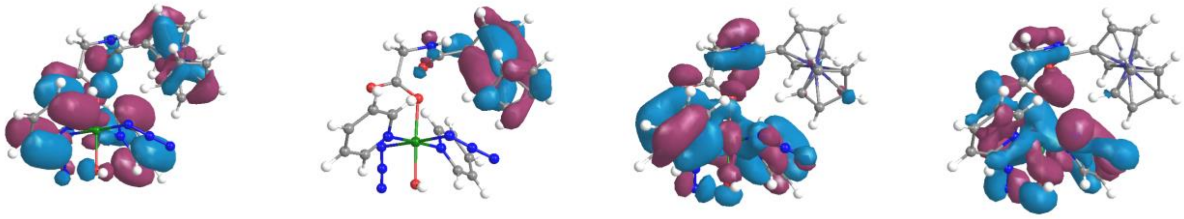

Tr17 (243 nm) Tr18 (235 nm) Tr19 (235 nm) Tr20 (234nm)

Particle

Hole

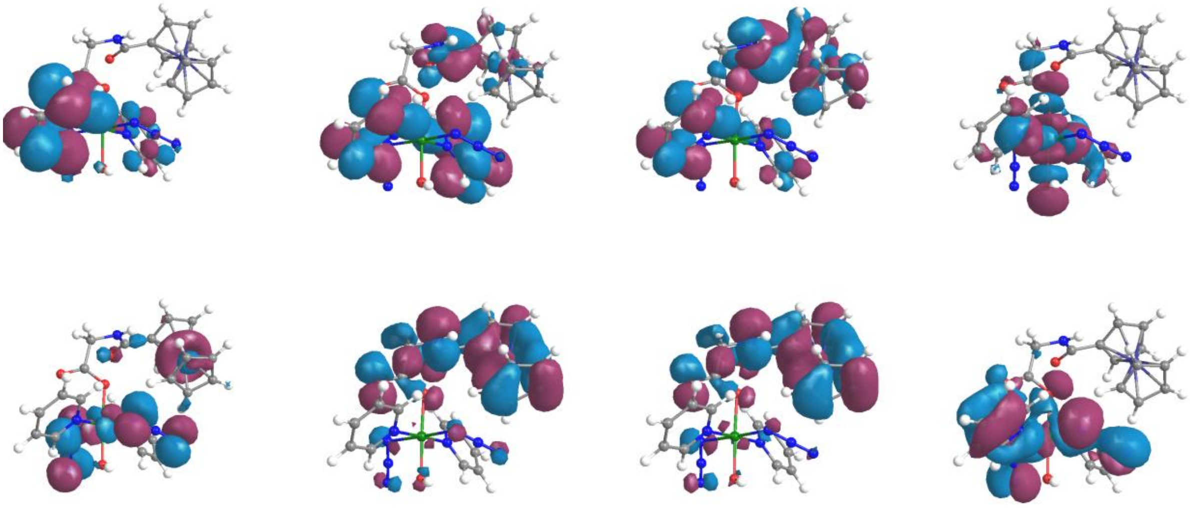

Tr21 (231 nm) Tr22 (228 nm)

Particle

Hole

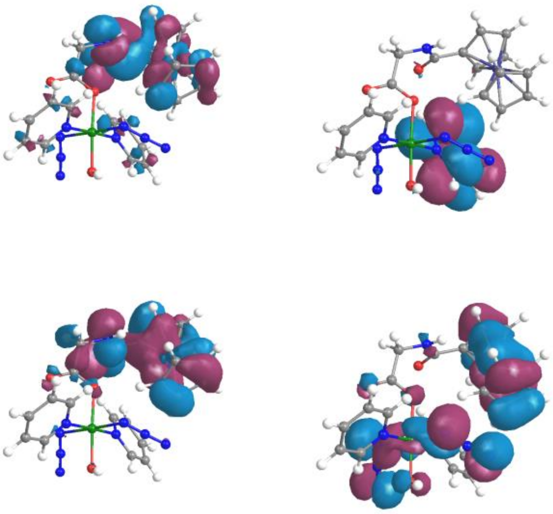

Figure S19. NTOs for transitions labelled 1-21 for **Pt-Fe**. Plots were generated with the Chemissian v4.67 software.[28]

**Pt-Fe**	T11 (457 nm)	T12 (434 nm)	T13 (431 nm)	T14 (420 nm)
Particle	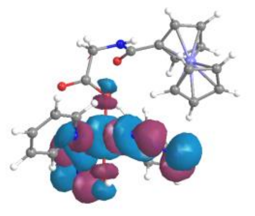	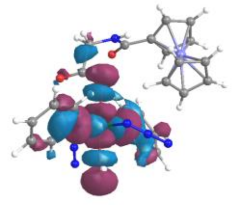	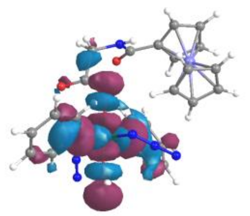	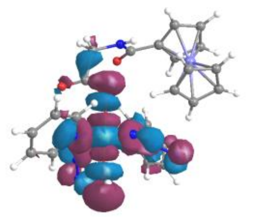
Hole	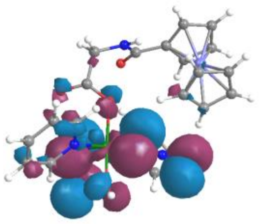	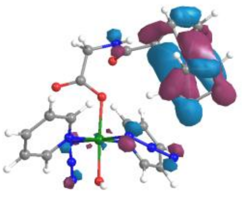	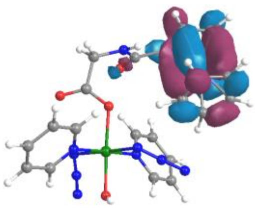	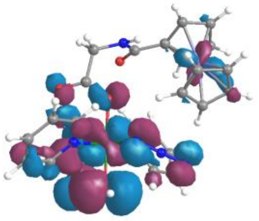
	T15 (414 nm)	T21 (352 nm)	T22 (350 nm)	T23 (344 nm)
Particle	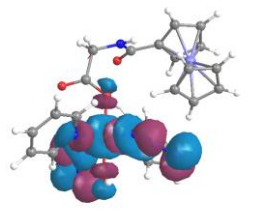	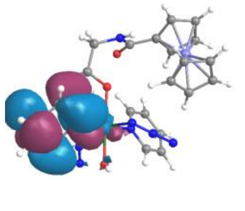	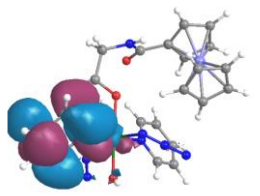	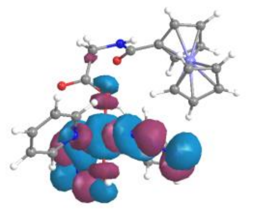
Hole	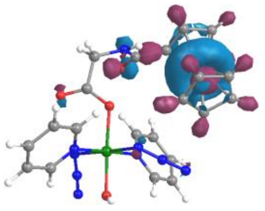	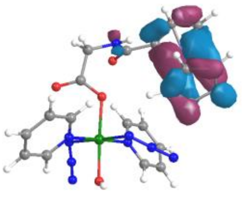	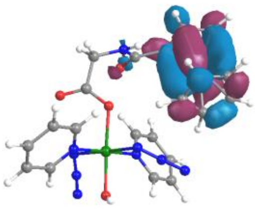	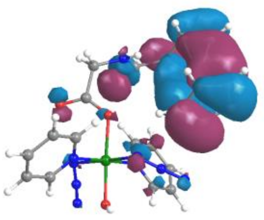
	T24 (343 nm)	T25 (341 nm)	T26 (338 nm)	T27 (332 nm)
Particle	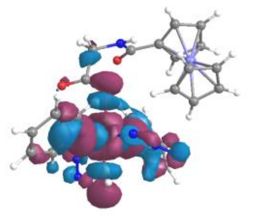	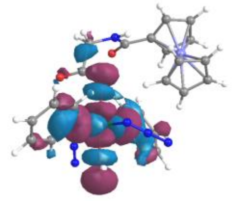	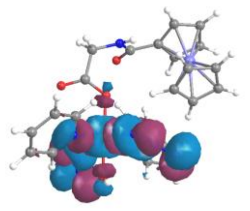	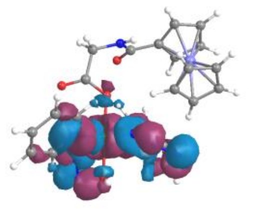

Hole

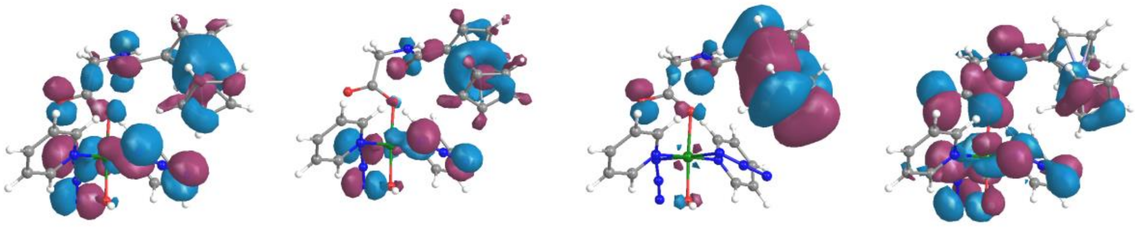

Figure S20. NTOs for **Pt-Fe** triplet states lying below the bright singlet state and the Tr7 transition. Plot were generated with the Chemissian v4.67 software.[10]

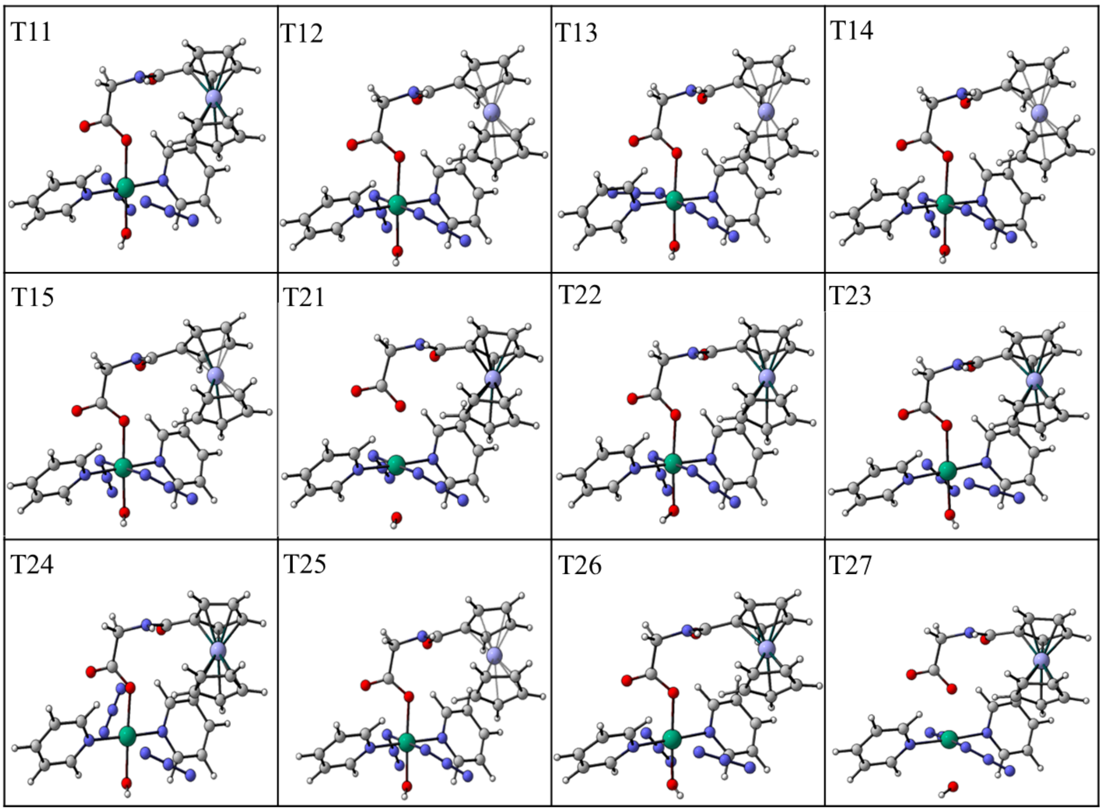

Figure S21. Optimised structures of excited triplet states of **Pt-Fe** obtained at the TD-DFT B3LYP/SDD/6-31G* level of theory.

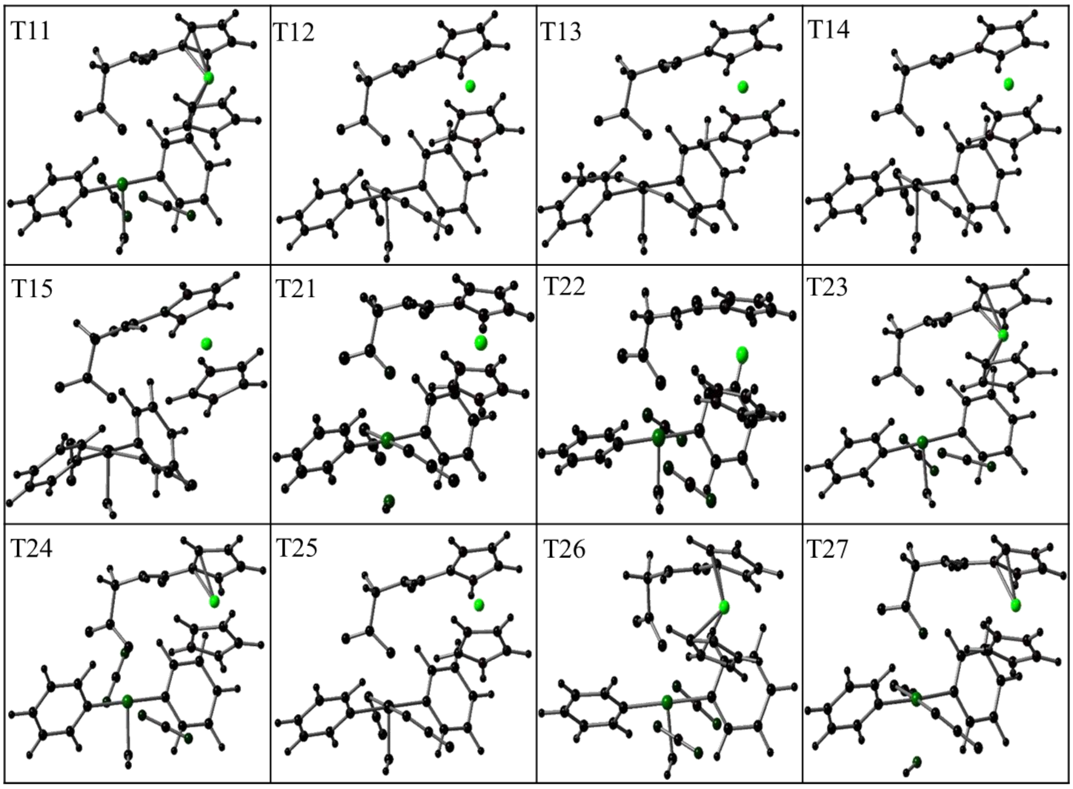

Figure S22. Spin density distributions of the optimised structures of excited triplet states of **Pt-Fe** obtained at the TD-DFT B3LYP/SDD/6-31G* level of theory. Green colour identifies the atoms where unpaired electrons are localised.

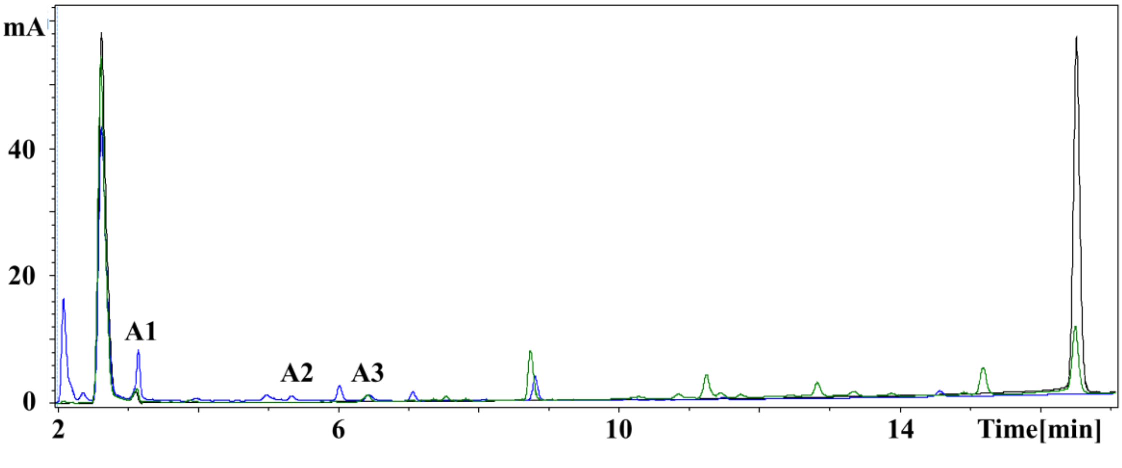

Figure S23. Photoreaction between **Pt-Fe** and 5′-GMP in aqueous solution at 298 K after 0 and 60 min irradiation monitored by HPLC, dark (—), blue light (—), green light (—).

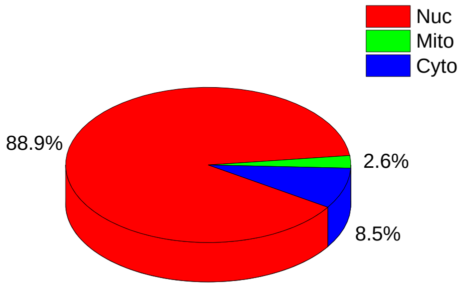

Figure S24. ICP-MS quantification of Pt distribution in A549 cells. Cells were treated with 10 μM **Pt-Fe** at 310 K for 1 h in the dark. Nuclei (Nuc), mitochondria (Mito), and cytoplasm excluding the mitochondrial component (Cyto), were isolated by using nuclear and mitochondrial isolation kits.

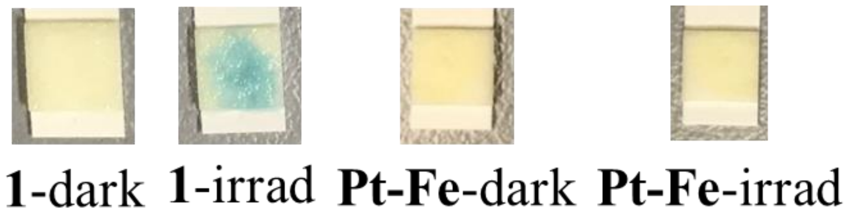

Figure S25. Colour change for aqueous solutions of complex **1** or **Pt-Fe** (5 mM) in the stained area on the paper zone of the Peroxide 25 Test Sticks (Quantofix®) in the absence or presence of irradiation.

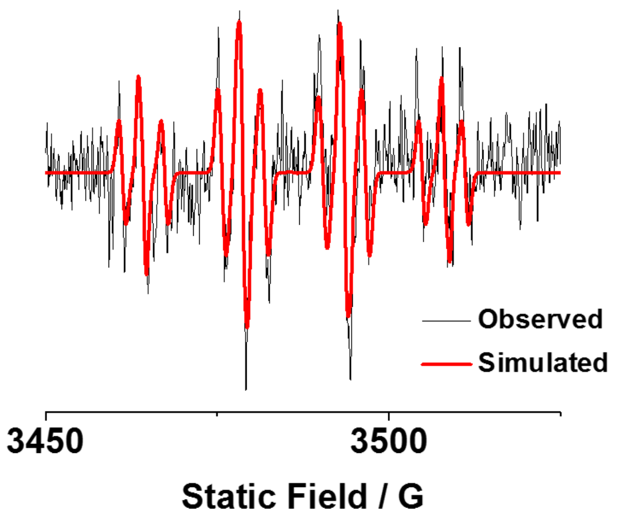

Figure S26. Observed (black) and simulated (red) EPR spectra of complex **Pt-Fe** (2.5 mM) in RPMI-1640 cell culture solution (containing 5% DMSO) showing the formation of DMPO−N_3• and DMPO−OH• adducts after irradiation (465 nm). The experimental trace is the accumulation of 200 scans (conversion time 5.12 ms, time constant 5.12 ms, and sweep time 10.49 s for each scan) with continuous irradiation (465 nm). Parameters used for simulation: DMPO−N_3• (g = 2.0054, $\boldsymbol{a}_{\mathbf{NO}}^{\mathbf{N}}$ = 1.46 mT, $\boldsymbol{a}_{\boldsymbol{\beta}}^{\mathbf{H}}$ = 1.46 mT, and $\boldsymbol{a}_{\mathbf{N\alpha}}^{\mathbf{N}}$ = 0.29 mT); DMPO−OH• (g = 2.0054, $\boldsymbol{a}_{\mathbf{NO}}^{\mathbf{N}}$ = 1.46 mT, and $\boldsymbol{a}_{\boldsymbol{\beta}}^{\mathbf{H}}$ = 1.46 mT).

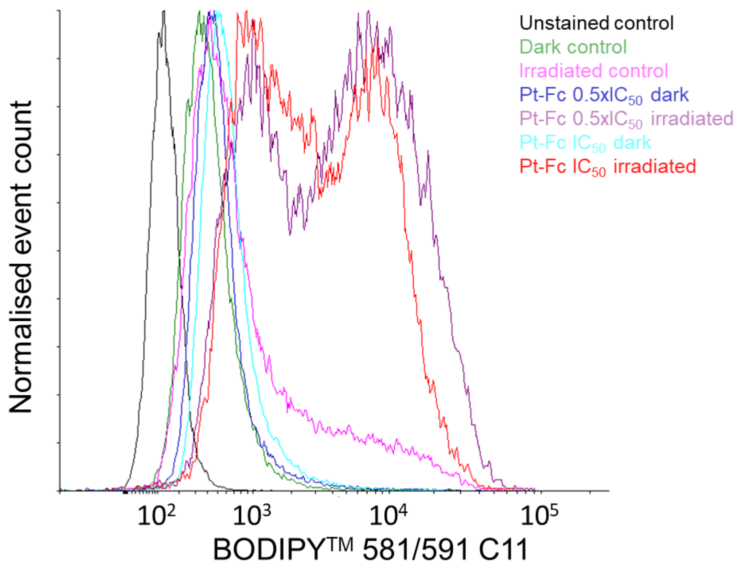

Figure S27. Lipid peroxidation assay of A549 cells stained by BODIPY™ 581/591 C11 ($\lambda_{ex}/\lambda_{em}$ = 488/500–560 nm) and analysed by flow cytometry. X-axis indicates the intensity of emission from oxidised BODIPY™ 581/591 C11.

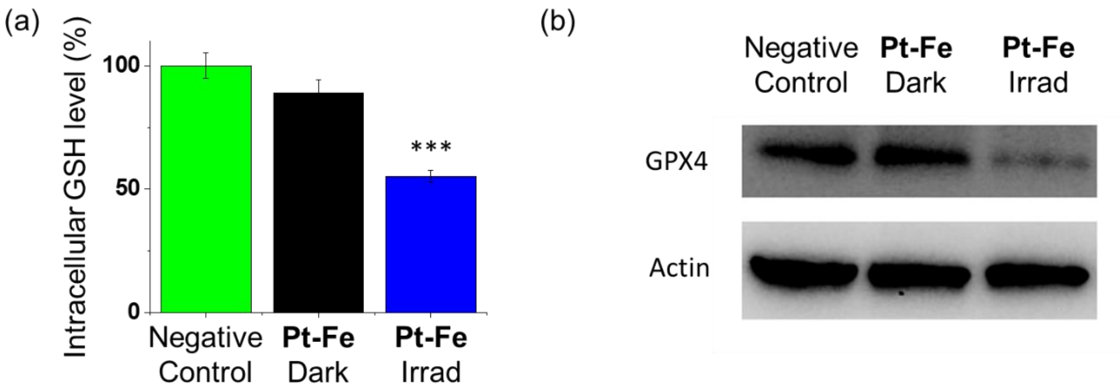

Figure S28. (a) Cellular GSH level determined in A549 cells after treatment with **Pt-Fe** (IC_{50}) in the dark or irradiated with blue light; (b) expression of GPX4 in A549 cells after treatment with **Pt-Fe** (IC_{50}) in the dark or irradiated with blue light. Actin was used as a reference for protein loading. Based on the negative control, the intensities of the GPX4 blots for the dark and irradiated cells were normalised as 1.07±0.08 and 0.32±0.14*, respectively. $^{*}p < 0.05$, $^{**}p < 0.01$, $^{***}p < 0.005$. All experiments were repeated twice independently.

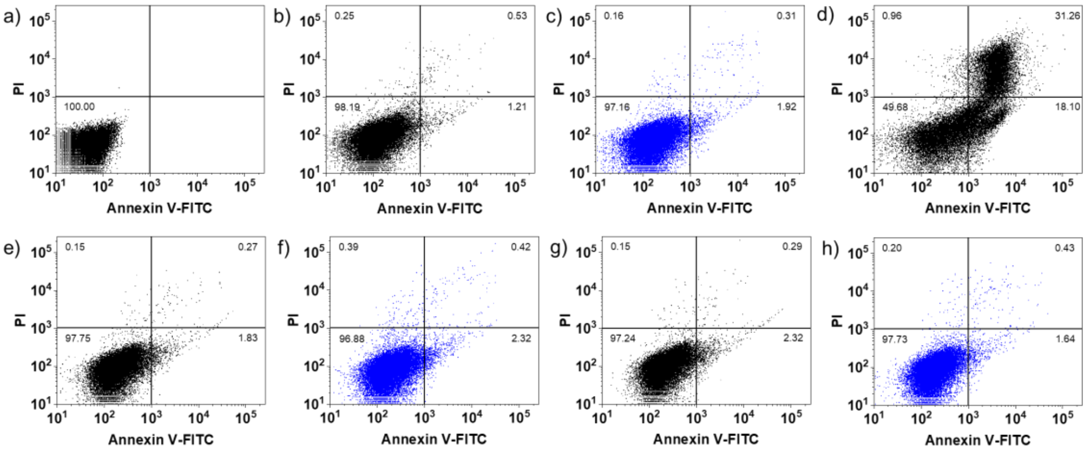

Figure S29. Cell apoptosis assay for A549 cells double stained by Annexin V-FITC/PI ($\lambda_{ex}/\lambda_{em}$ = 488/500–560 nm for Annexin V-FITC, $\lambda_{ex}/\lambda_{em}$ = 488/645–735 nm for PI) and analysed by flow cytometry. a) unstained A549 cells as negative control; b) untreated A549 cells in the dark; c) untreated A549 cells irradiated with blue light (465 nm); d) A549 cells treated with 50 μM cisplatin for 48 h in the dark; e) A549 cells treated with **Pt-Fe** (0.5×IC_{50}) in the dark and f) irradiated with blue light; g) A549 cells treated with **Pt-Fe** (IC_{50}) in the dark and h) irradiated with blue light. No apparent apoptosis is observed for cells treated with **Pt-Fe**.

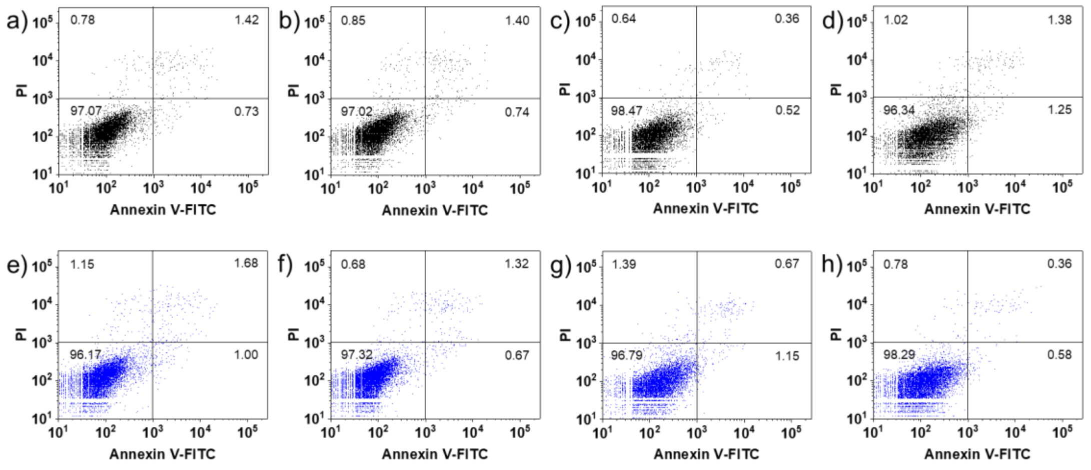

Figure S30. Cell apoptosis assay of A2780 and A2780cis cells under normoxia double stained by Annexin V-FITC/PI ($\lambda_{ex}/\lambda_{em}$ = 488/500–560 nm for Annexin V-FITC, $\lambda_{ex}/\lambda_{em}$ = 488/645–735 nm for PI) and analysed by flow cytometry. a) untreated A2780 cells in the dark; b) A2780 cells treated with **Pt-Fe** (IC_{50}) in the dark; c) untreated A2780cis cells in the dark; d) A2780cis cells treated with **Pt-Fe** (IC_{50}) in the dark; e) untreated A2780 cells irradiated with blue light (465 nm); f) A2780 cells treated with **Pt-Fe** (IC_{50}) irradiated with blue light; g) untreated A2780cis cells irradiated with blue light; h) A2780cis cells treated with **Pt-Fe** (IC_{50}) irradiated with blue light. No apparent apoptosis is observed for cells treated with **Pt-Fe**.

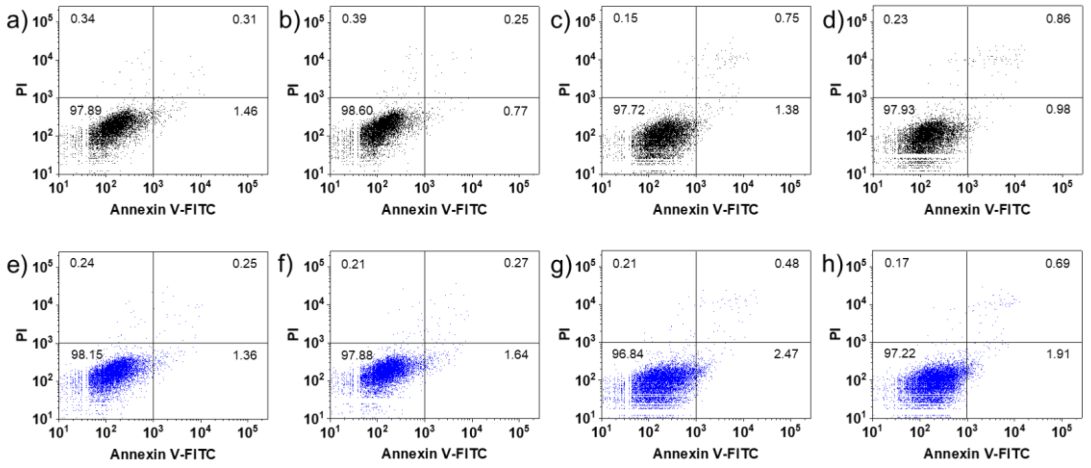

Figure S31. Cell apoptosis assay of A2780 and A2780cis cells under hypoxia double stained by Annexin V-FITC/PI ($\lambda_{ex}/\lambda_{em}$ = 488/500–560 nm for Annexin V-FITC, $\lambda_{ex}/\lambda_{em}$ = 488/645–735 nm for PI) and analysed by flow cytometry. a) untreated A2780 cells in the dark; b) A2780 cells treated with **Pt-Fe** (IC_{50}) in the dark; c) untreated A2780cis cells in the dark; d) A2780cis cells treated with **Pt-Fe** (IC_{50}) in the dark; e) untreated A2780 cells irradiated with blue light (465 nm); f) A2780 cells treated with **Pt-Fe** (IC_{50}) irradiated with blue light; g) untreated A2780cis cells irradiated with blue light; h) A2780cis cells treated with **Pt-Fe** (IC_{50}) irradiated with blue light. No apparent apoptosis is observed for cells treated with **Pt-Fe**.

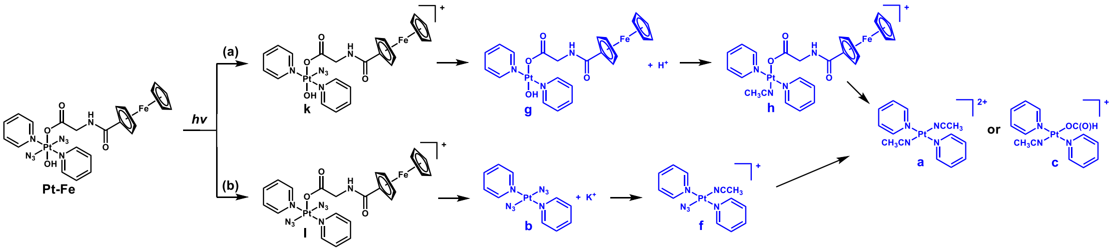

Figure S32. Possible photodecomposition pathways for **Pt-Fe** in aqueous solution on irradiation with indigo light (420 nm), as indicated by fragments observed in mass spectra (for details and labelling of species see **Table S5** and **Fig. S11**). Pt(IV) species in black, Pt(II) species in blue. CH_3CN and $HCOO^-$ are from the HPLC mobile phase.

References

1. O. V. Dolomanov, L. J. Bourhis, R. J. Gildea, J. A. K. Howard, H. Puschmann, *J. Appl. Crystallogr.*, 2009, **42**, 339−341.
2. G. M. Sheldrick, *Acta Crystallogr.*, Sect. A: Found. Adv., 2015, **71**, 3−8.
3. G. M. Sheldrick, *Acta Crystallogr., Sect. C: Struct. Chem.*, 2015, **71**, 3−8.
4. M. J. Frisch, G. W. Trucks, H. B. Schlegel, G. E. Scuseria, M. A. Robb, J. R. Cheeseman, G. Scalmani, V. Barone, G. A. Petersson, H. Nakatsuji, X. Li, M. Caricato, A.V. Marenich, J.Bloino, B. G. Janesko, R. Gomperts, B. Mennucci, H. P. Hratchian, J. V. Ortiz, A. F. Izmaylov, J. L. Sonnenberg, D. Williams-Young, F. Ding, F. Lipparini, F. Egidi, J. Goings, B. Peng, A. Petrone, T. Henderson, D. Ranasinghe, V. G. Zakrzewski, J. Gao, N. Rega, G. Zheng, W. Liang, M. Hada, M. Ehara, K. Toyota, R. Fukuda, J. Hasegawa, M. Ishida, T. Nakajima, Y. Honda, O. Kitao, H. Nakai, T. Vreven, K. Throssell, J. A. Montgomery Jr., J. E. Peralta, F. Ogliaro, M. J. Bearpark, J. J. Heyd, E. N. Brothers, K. N. Kudin, V. N. Staroverov, T. A. Keith, R. Kobayashi, J. Normand, K. Raghavachari, A. P. Rendell, J. C. Burant, S. S. Iyengar, J. Tomasi, M. Cossi, J. M. Millam, M. Klene, C. Adamo, R. Cammi, J. W. Ochterski, R. L. Martin, K. Morokuma, O. Farkas, J. B. Foresman, D. J. Fox, *GaussView 5.0. Wallingford, E.U.A.* 2016**.**
5. A. D. Becke, *The Journal of Chemical Physics*, 1993, **98**, 5648–5652.
6. C. Lee, W. Yang, R. G. Parr, *Phys Rev B*, 1988, **37**, 785–789.
7. S. Grimme, J. Antony, S. Ehrlich and H. Krieg, *J Chem Phys*, 2010, **132**, 154104.
8. A. V. Marenich, C. J. Cramer and D. G. Truhlar, *J. Phys. Chem. B*, 2009, **113**, 6378–6396.
9. D. Andrae, U. Häußermann, M. Dolg, H. Stoll and H. Preuß, *Theoret. Chim. Acta*, 1990, **77**, 123–141.
10. J. P. Perdew, J. A. Chevary, S. H. Vosko, K. A. Jackson, M. R. Pederson, D. J. Singh and C. Fiolhais, *Phys. Rev. B*, 1992, **46**, 6671–6687.
11. J. P. Perdew and Y. Wang, *Phys. Rev. B*, 1992, **45**, 13244–13249.
12. T. Yanai, D. P. Tew and N. C. Handy, *Chemical Physics Letters*, 2004, **393**, 51–57.
13. J. Tao, J. P. Perdew, V. N. Staroverov and G. E. Scuseria, *Phys. Rev. Lett.*, 2003, **91**, 146401.
14. M. Ernzerhof and G. E. Scuseria, *J. Chem. Phys.*, 1999, **110**, 5029–5036.
15. C. Adamo and V. Barone, *J. Chem. Phys.*, 1999, **110**, 6158–6170.

16. Y. Zhao, N. E. Schultz, D. G. Truhlar, *J. Chem. Phys.* 2005, **123**, 161103.
17. Y. Zhao and D. G. Truhlar, *Theor Chem Account*, 2008, **120**, 215–241.
18. Y. Zhao and D. G. Truhlar, *J. Chem. Phys.*, 2006, **125**, 194101.
19. R. Peverati and D. G. Truhlar, *J. Phys. Chem. Lett.*, 2011, **2**, 2810–2817.
20. R. Peverati and D. G. Truhlar, *Phys. Chem. Chem. Phys.*, 2012, **14**, 13171–13174.
21. F. Neese, *WIREs Computational Molecular Science*, 2018, **8**, e1327.
22. F. Neese, *Wiley Interdiscip. Rev. Comput. Mol. Sci.* 2012, **2**, 73–78.
23. D. Escudero and W. Thiel, *J. Chem. Phys.*, 2014, **140**, 194105.
24. D. Escudero, E. Heuser, R. J. Meier, M. Schäferling, W. Thiel and E. Holder, *Chem. Eur. J.*, 2013, **19**, 15639–15644.
25. C. M. Marian, WIREs *Comput. Mol. Sci*. 2012, **2**, 187–203.
26. V. Vichai, K. Kirtikara, *Nat. Protoc*., 2006, **1**, 1112−1116.
27. H. Shi, Q. Wang, V. Venkatesh, G. Feng, L. S. Young, I. Romero-Canelón, M. Zeng and P. J. Sadler, *Dalton Trans.*, 2019, **48**, 8560–8564.
28. L. Skripnikov, Chemissian 4.67, 2020, https://www.chemissian.com.
